# Evidence supported by Mendelian randomization: impact on inflammatory factors in knee osteoarthritis

**DOI:** 10.3389/fmed.2024.1382836

**Published:** 2024-05-28

**Authors:** Lilei Xu, Jiaqi Ma, Qing Yu, Kean Zhu, Xuewen Wu, Chuanlong Zhou, Xianming Lin

**Affiliations:** ^1^Third Clinical Medical College, Zhejiang Chinese Medical University, Hangzhou, China; ^2^Department of Acupuncture, Third Affiliated Hospital of Zhejiang Chinese Medical University, Hangzhou, China

**Keywords:** knee osteoarthritis, biomarkers, Mendelian randomization, GWAS, inflammation

## Abstract

**Background:**

Prior investigations have indicated associations between Knee Osteoarthritis (KOA) and certain inflammatory cytokines, such as the interleukin series and tumor necrosis factor-alpha (TNFα). To further elaborate on these findings, our investigation utilizes Mendelian randomization to explore the causal relationships between KOA and 91 inflammatory cytokines.

**Methods:**

This two-sample Mendelian randomization utilized genetic variations associated with KOA from a large, publicly accessible Genome-Wide Association Study (GWAS), comprising 2,227 cases and 454,121 controls of European descent. The genetic data for inflammatory cytokines were obtained from a GWAS summary involving 14,824 individuals of European ancestry. Causal relationships between exposures and outcomes were primarily investigated using the inverse variance weighted method. To enhance the robustness of the research results, other methods were combined to assist, such as weighted median, weighted model and so on. Multiple sensitivity analysis, including MR-Egger, MR-PRESSO and leave one out, was also carried out. These different analytical methods are used to enhance the validity and reliability of the final results.

**Results:**

The results of Mendelian randomization indicated that Adenosine Deaminase (ADA), Fibroblast Growth Factor 5(FGF5), and Hepatocyte growth factor (HFG) proteins are protective factors for KOA (IVW_ADA_: OR = 0.862, 95% CI: 0.771–0.963, *p* = 0.008; IVW_FGF5_: OR = 0.850, 95% CI: 0.764–0.946, *p* = 0.003; IVW_HFG_: OR = 0.798, 95% CI: 0.642–0.991, *p* = 0.042), while Tumor necrosis factor (TNFα), Colony-stimulating factor 1(CSF1), and Tumor necrosis factor ligand superfamily member 12(TWEAK) proteins are risk factors for KOA. (IVW_TNFα_: OR = 1.319, 95% CI: 1.067–1.631, *p* = 0.011; IVW_CSF1_: OR = 1.389, 95% CI: 1.125–1.714, *p* = 0.002; IVW_TWEAK_: OR = 1.206, 95% CI: 1.016–1.431, *p* = 0.032).

**Conclusion:**

The six proteins identified in this study demonstrate a close association with the onset of KOA, offering valuable insights for future therapeutic interventions. These findings contribute to the growing understanding of KOA at the microscopic protein level, paving the way for potential targeted therapeutic approaches.

## 1 Introduction

Knee osteoarthritis (KOA) is a common degenerative joint disease characterized by subchondral bone remodeling (1), meniscal degeneration (2), and inflammation of the infrapatellar fat pad and synovium (3), significantly impacting people’s health and quality of life. Studies indicate that over 80% of KOA patients are over 60 years old, and the condition significantly contributes to annual hospital admissions in developed countries (4). The onset of KOA is associated with factors such as aging (5, 6), obesity (7), diabetes (8), joint trauma (6, 9), and overuse, which lead to cartilage damage, inflammation, and a loss of the knee joint’s homeostasis. Symptoms typically include knee pain (10), swelling (11), and crepitus (11). The development of this pathological condition is closely tied to the regulation of intrinsic mechanisms, and only by understanding these mechanisms can we precisely and effectively halt the further progression of KOA.

In recent years, the research in the field of KOA has been increasingly in-depth. Current studies focus on cytokines, particularly those crucial to inflammation (12, 13), as well as the mechanisms of gene expression (14, 15) and signal transduction pathways (16–18). The TNF family and MMP enzyme series have been frequently reported to be associated with KOA (13, 19–22). While there is no clear consensus on the pathogenesis of KOA, there is widespread support for inflammatory changes playing a key role in its development. KOA is thought to result from an imbalance between anti-inflammatory and pro-inflammatory factors (6, 23–25). Therefore, this study employs Mendelian randomization analysis to comprehensively explore the causal relationships between various inflammatory factors and KOA, aiming to provide a reference for the future treatment of KOA.

Mendelian randomization (MR) serves as a potent analytical tool employed to infer the causal impact of an exposure on an outcome by leveraging genetic variations within non-experimental datasets (26). The random allocation of alleles during meiosis equips MR with the unique capability to address both conventional confounding variables and reverse causation, thereby furnishing more robust evidence for causal inference (27). The use of a two-sample MR analysis enhances researchers’ ability to scrutinize the relationships between instruments and exposures, as well as instruments and outcomes, across distinct population samples, thereby amplifying the versatility and efficacy of the analysis (28). In this study, we meticulously extracted authentic genetic variants from the summary data of a genome-wide association study (GWAS) comprising 91 inflammatory cytokines, with the aim of exploring their intricate correlations with KOA.

## 2 Materials and methods

### 2.1 MR assumptions

**Figure 1:**
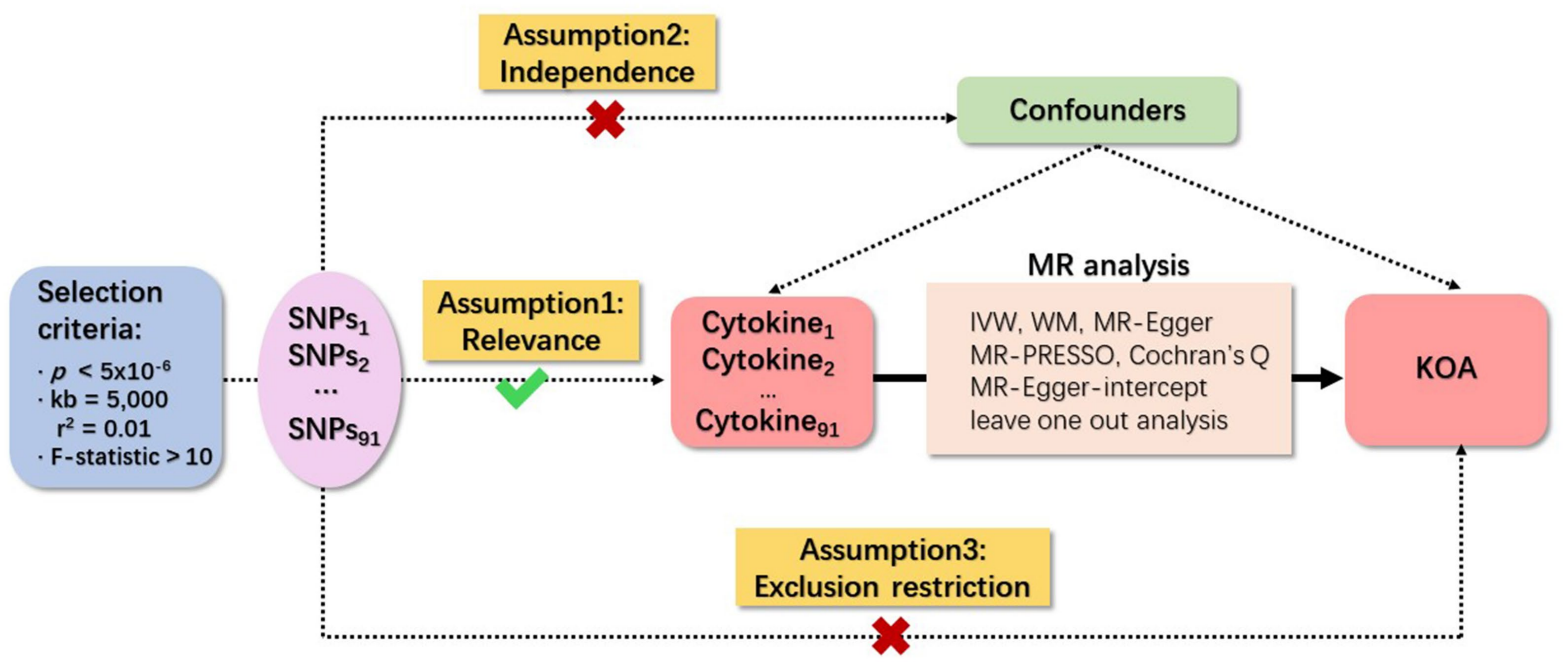
Schematic representation of study design in Mendelian randomization (MR) analysis. Significant instrumental variables were chosen for 91 inflammatory cytokines and KOA, facilitating an examination of the potential causal relationships between each protein and the occurrence of KOA. This flowchart illustrates the three basic assumptions of MR analysis, namely, dependence, independence, and exclusion restrictions.

Three fundamental assumptions underpin MR analyses, specifically relevance, independence, and exclusion restriction (29). The foundational premise is that the selected genetic variants demonstrate an association with the risk factor (relevance), remain unaffected by any confounding factors in the risk factor–outcome association (independence), and exert no influence on the outcome through pathways other than the targeted risk factor (exclusion restriction). In this study, two GWAS were employed to identify genetically significant single nucleotide polymorphisms (SNPs) associated with 91 inflammatory cytokines and KOA. An overview of the study design is illustrated in Figure 1.

### 2.2 Instrumental variable selection

Initially, a genome-wide significance threshold of *p* < 5 × 10^−6^ was established to identify strongly associated SNPs with inflammatory cytokines and KOA. Subsequently, to mitigate the impact of linkage disequilibrium, we performed clumping on these SNPs (kb = 5,000, *r*^2^ = 0.01). Palindromic SNPs were excluded from the analysis due to uncertainty regarding their alignment in the same direction for exposure and outcome in the GWAS of systemic inflammatory regulators. In the third step, the R2 value of each SNP was utilized to calculate the proportion of variance in exposure, and the F-statistic was employed to estimate the instrument strength, thereby preventing potential biases associated with weak instruments.

### 2.3 Data source

The dataset encompassing GWAS for 91 circulating inflammatory cytokines was obtained from the investigation conducted by Zhao et al. (30). In their research publication, the authors conducted protein quantitative trait loci (pQTL) mapping in a cohort of 14,824 individuals of European ancestry. The primary objective was to identify genetic variants linked to circulating inflammatory proteins by summarizing pQTL information within ±1 megabase surrounding 91 candidate genes associated with these proteins. Additionally, the GWAS summary statistics for KOA are accessible for download from the study by Jiang et al. (31). This dataset includes 2,227 cases of European ancestry with KOA and 454,121 controls of European ancestry. Importantly, there is no overlap in the selection of populations between the exposure group and the outcome group.

### 2.4 Statistical analysis

The Inverse Variance-Weighted analysis (IVW), a classical method in MR, mandates strict adherence to the fundamental assumptions of instrumental variables for all SNPs. The IVW method yields consistent estimates of causal effects with robust testing power when these instrumental variable assumptions are met. Therefore, the results of the IVW method were taken as the main results in our study. Despite its widespread use in practical applications, the IVW method’s assumption that all genetic variations serve as valid instrumental variables may face practical challenges (32). Consequently, this study incorporated supplementary MR methods based on distinct assumptions. The Weighted Median (WM) requires that 50% or more of SNPs function as valid instrumental variables to ensure the stability of effect values. The core idea behind WM is to utilize the median of Wald estimates from all SNPs as the ultimate MR effect value (33). This effectively mitigates the impact of outlier SNPs on results, enhancing result robustness. In situations where significant heterogeneity is observed in IVW estimation results, median-based methods can offer a valuable supplementary approach. In contrast to IVW, the MR-Egger method modifies the conventional requirement for the regression line to pass through the origin. This is achieved by introducing an intercept term in weighted linear regression to examine and correct overall horizontal pleiotropy (34). MR-Egger regression relaxes the exclusivity assumption of instrumental variables in MR methods. MR-Egger can generate consistent estimates of causal effects only when the direct effects of SNPs on outcomes are independent of the associations between SNPs and exposures (34). However, utilizing the same SNPs for IVW analysis when the intercept term is non-zero or statistically insignificant may introduce bias into results. Compared to other methods, MR-Egger exhibits lower testing power, potentially resulting in wider confidence intervals. Moreover, the method’s assumptions require that SNPs exhibit the same horizontal pleiotropy, a condition challenging to fully satisfy in practical applications.

Sensitivity analysis encompasses heterogeneity testing and pleiotropy testing, primarily investigated from three perspectives. (a) Heterogeneity testing aims to evaluate disparities among various instrumental variables (IVs). The Q-test method is applied to assess heterogeneity in both the MR-IVW model and MR-Egger model, conducting tests among instrumental variables using distinct statistical analysis methods. A *p*-value exceeding 0.05 indicates the absence of heterogeneity. In cases where heterogeneity is identified among instrumental variables, a random effects model (REM) based on the IVW method is employed to evaluate the impact of exposure on the outcome. (b) Pleiotropy testing primarily scrutinizes whether multiple IVs display horizontal pleiotropy, i.e., whether SNPs functioning as IVs are associated with other unrelated variables. The MR-Egger method is commonly utilized for assessing horizontal pleiotropy, where the intercept term indicates its presence if significantly different from zero (*p* < 0.05) (34). Additionally, the MR-PRESSO is utilized to identify and eliminate outlier SNPs, correcting horizontal pleiotropy and providing more robust causal estimates between exposure and outcome (35). (c) Leave-one-out sensitivity testing involves systematically removing each SNP locus using the leave-one-out method. MR analysis is then conducted using the remaining SNP loci to assess whether a specific SNP locus introduces bias into the results. Analyses were implemented by the package TwoSampleMR (version 0.4.25) and MR-PRESSO (version 1.0) in R (version 4.3.1).

## 3 Result

### 3.1 The selection results of instrumental variables

In adherence to the specified criteria for instrumental variable selection, we conducted a meticulous screening of the dataset encompassing diverse proteins to identify instrumental variables meeting the necessary conditions. Here, we present the outcome data solely for positively associated proteins. Taking the ADA protein as an illustrative case, we identified 21 SNPs from the GWAS dataset that exhibited close associations (*p* < 5 × 10^−6^), demonstrated no linkage disequilibrium, and overlapped with the dataset for KOA. We excluded one palindrome SNP (rs2620728). Using the PhenoScanner database,^1^ we searched for secondary phenotypes of the selected SNPs and excluded the potential confounding SNP (rs1608554). The F-statistic values for the remaining SNPs ranged from 20.91 to 1180.03, confirming the absence of weak instrumental variables in the MR analysis. Simultaneously, MR-PRESSO analysis identified no outliers. Ultimately, 19 SNPs were selected as instrumental variables for evaluating the association between ADA protein and KOA. Comprehensive information regarding SNPs for other positively associated proteins is presented in Table 1.

**Table 1:** Summary of protein causally associated with knee osteoarthritis.

Data	Proteins	Initial / Final SNPs	MR-PRESSO P-Global	*F*	Confounding SNPs	Palindrome sequence
GCST90274759	ADA (Adenosine Deaminase)	21/19	0.632	20.91–1180.03	rs1608554	rs2620728
GCST90274776	CSF1 (Colony-stimulating factor 1)	20/17	0.617	20.88–203.64	rs116443177 rs2523992	NA
GCST90274790	FGF5 (Fibroblast Growth Factor 5)	35/31	0.658	20.85–1233.21	NA	rs10961630
GCST90274793	HGF (Hepatocyte growth factor)	23/19	0.745	20.89–86.58	rs851612	NA
GCST90274839	TNF (Tumor necrosis factor)	18/18	0.561	20.86–27.16	NA	NA
GCST90274846	TWEAK (Tumor necrosis factor ligand superfamily member 12)	34/23	0.458	20.86–27.16	rs11738159 rs13107325 rs2738752 rs579459 rs74351250	rs73133996

### 3.2 Outcomes of Mendelian randomization

The IVW method unveiled a potential inverse association between genetically determined elevated levels of ADA protein (corresponding to a one-standard-deviation increase) and a 13.8% reduction in the odds of developing KOA (OR = 0.862, 95% CI: 0.771–0.963, *p* = 0.008). This finding was corroborated by results from the weighted median method (OR = 0.866, 95% CI: 0.758–0.991, *p* = 0.037). Although MR-Egger analysis did not identify a statistically significant association, it indicated a consistent trend (OR = 0.875, 95% CI: 0.759–1.009, *p* = 0.083). Similar causal associations were observed for FGF5 *p*rotein and KOA (IVW: OR = 0.850, 95% CI: 0.764–0.946, *p* = 0.003; weighted median: OR = 0.814, 95% CI: 0.709–0.934, *p* = 0.003). The HGF protein was identified as a potential protective factor for KOA (IVW: OR = 0.798, 95% CI: 0.642–0.991, *p* = 0.042; weighted median: OR = 0.786, 95% CI: 0.576–1.072, *p* = 0.129).

**Figure 2:**
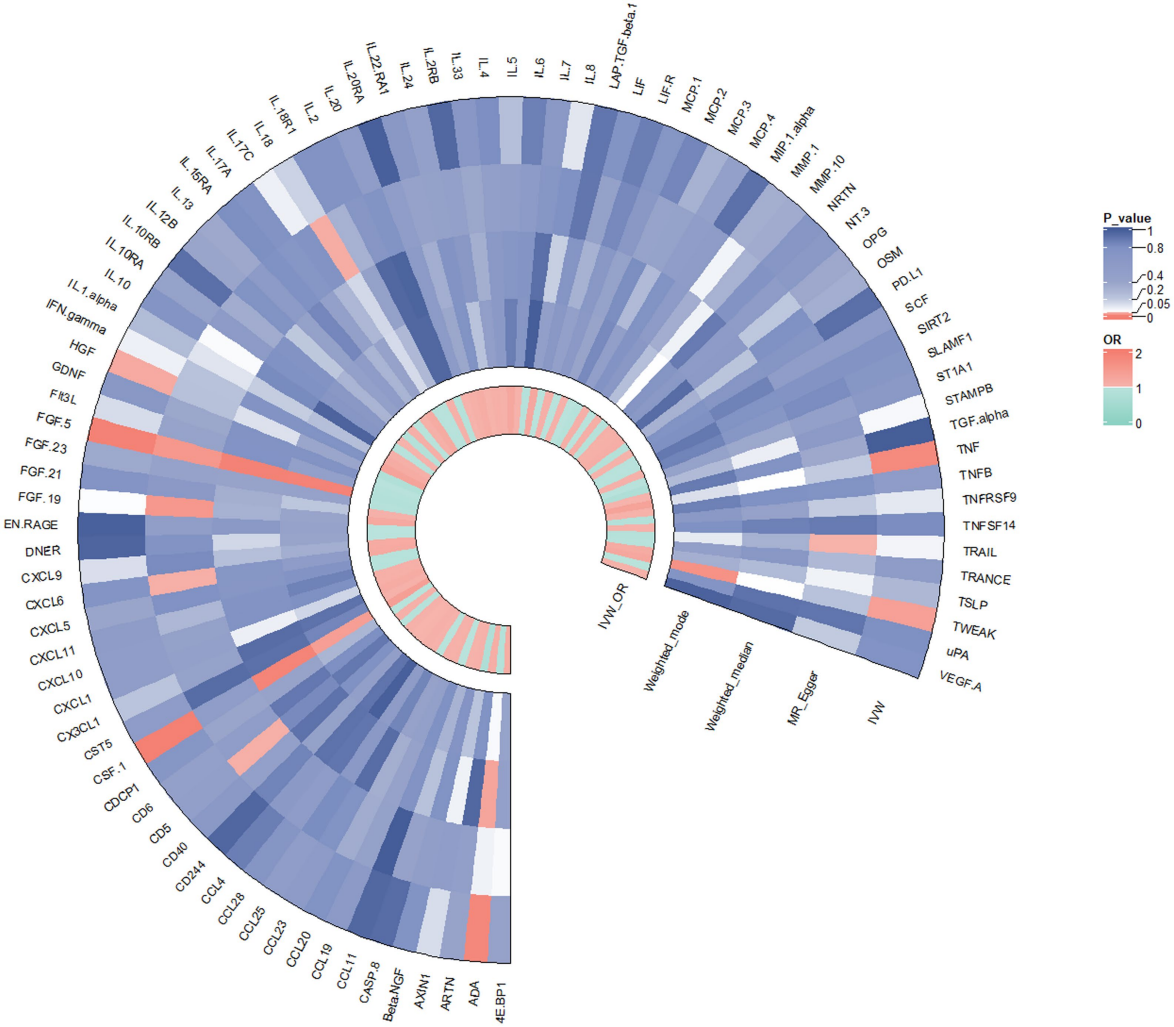
Result of the two-sample MR analysis of 91 and KOA: ADA (IVW: OR = 0.862, 95% CI: 0.771–0.963, *p* = 0.008; WM: OR = 0.866, 95% CI: 0.758–0.991, *p* = 0.037). FGF5 (IVW: OR = 0.850, 95% CI: 0.764–0.946, *p* = 0.003; WM: OR = 0.814, 95% CI: 0.709–0.934, *p* = 0.003). The HGF (IVW: OR = 0.798, 95% CI: 0.642–0.991, *p* = 0.042; WM: OR = 0.786, 95% CI: 0.576–1.072, *p* = 0.129).TNFα (IVW: OR = 1.319, 95% CI: 1.067–1.631, *p* = 0.011; WM: OR = 1.356, 95% CI: 0.995–1.848, *p* = 0.054), CSF1 (IVW: OR = 1.389, 95% CI: 1.125–1.714, *p* = 0.002; WM: OR = 1.544, 95% CI: 1.135–2.099, *p* = 0.006), TWEAK (IVW: OR = 1.206, 95% CI: 1.016–1.431, *p* = 0.032; WM: OR = 1.267, 95% CI: 0.997–1.610, *p* = 0.053).CD5 (MR Egger: OR = 0.621, 95% CI: 0.400–0.964,*p* = 0.046), CXCL6 (MR Egger: OR = 1.452, 95% CI: 1.205–1.749, *p* = 0.046), FGF19 (MR Egger:OR = 1.780, 95% CI: 1.126–2.815, *p* = 0.023), IL18 (MR Egger:OR = 0.878, 95% CI: 0.777–0.991,*p* = 0.044),TRAIL (MR Egger:OR = 0.808, 95% CI: 0.661–0.988, *p* = 0.049).

**Figure 3:**
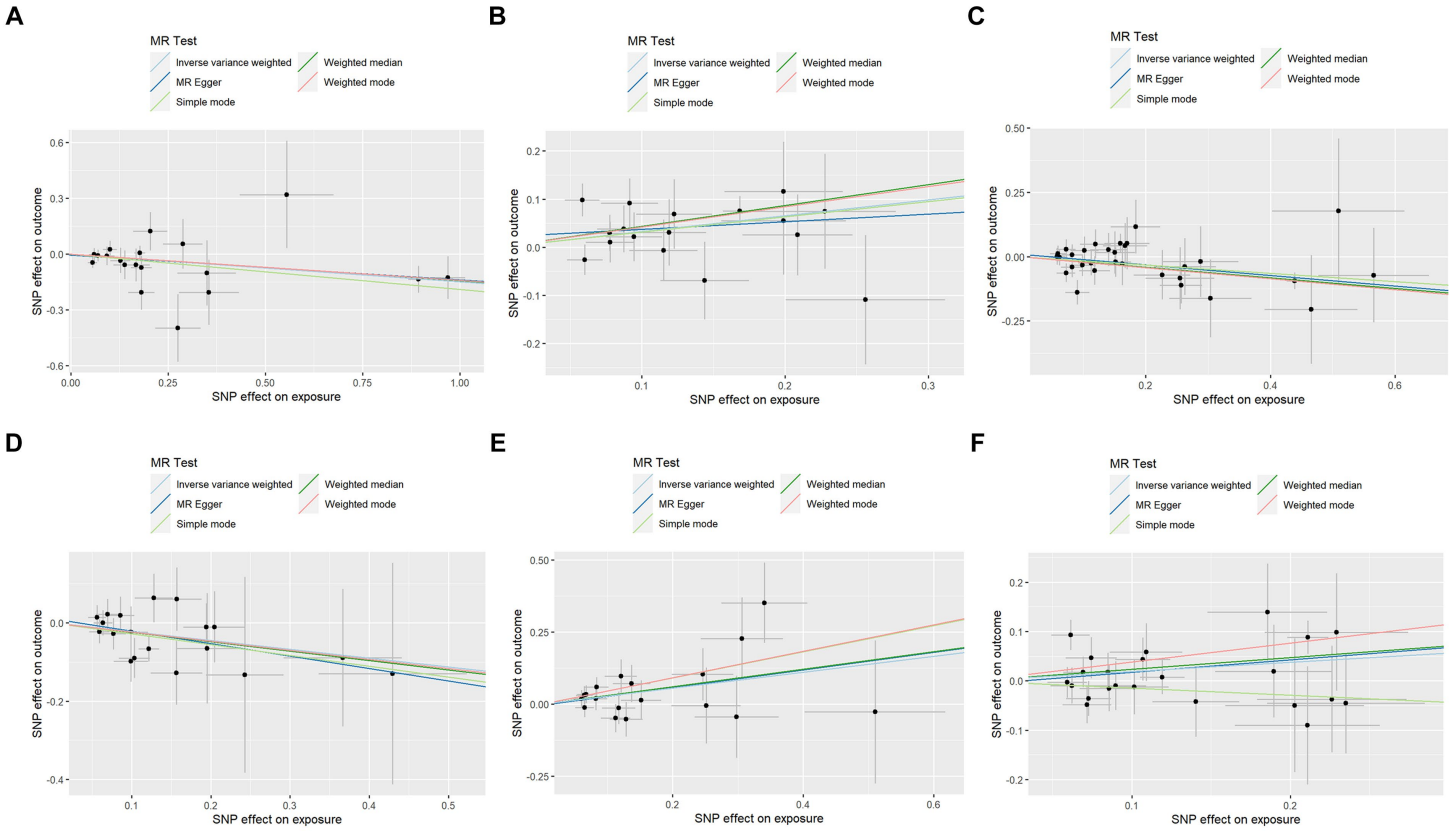
Scatter plots of Mendelian randomization analyses between KOA and 91 inflammatory cytokines. Individual inverse variance (IV) associations with KOA risk are displayed versus individual IV associations with cytokines in black dots. The 95%CI of the odds ratio for each IV is shown by the vertical and horizontal lines. The slope of the lines represents the estimated causal effect of the MR methods. **(A–F)**: ADA, CSF1, FGF5, HGF, TNFα, and TWEAK.

In contrast to the aforementioned proteins, our study revealed a significant increase in the risk of KOA associated with TNF (IVW: OR = 1.319, 95% CI: 1.067–1.631, *p* = 0.011; weighted median: OR = 1.356, 95% CI: 0.995–1.848, *p* = 0.054), CSF1 (IVW: OR = 1.389, 95% CI: 1.125–1.714, *p* = 0.002; weighted median: OR = 1.544, 95% CI: 1.135–2.099, *p* = 0.006), and TWEAK (IVW: OR = 1.206, 95% CI: 1.016–1.431, *p* = 0.032; weighted median: OR = 1.267, 95% CI: 0.997–1.610, *p* = 0.053). These proteins may serve as potential risk factors for the development of KOA. Results from the MR analysis of all inflammatory factors are depicted in Figure 2. The scatter plots of Mendelian randomization analyses for the aforementioned six proteins are exhibited in Figure 3.

### 3.3 Quality assurance

**Figure 4:**
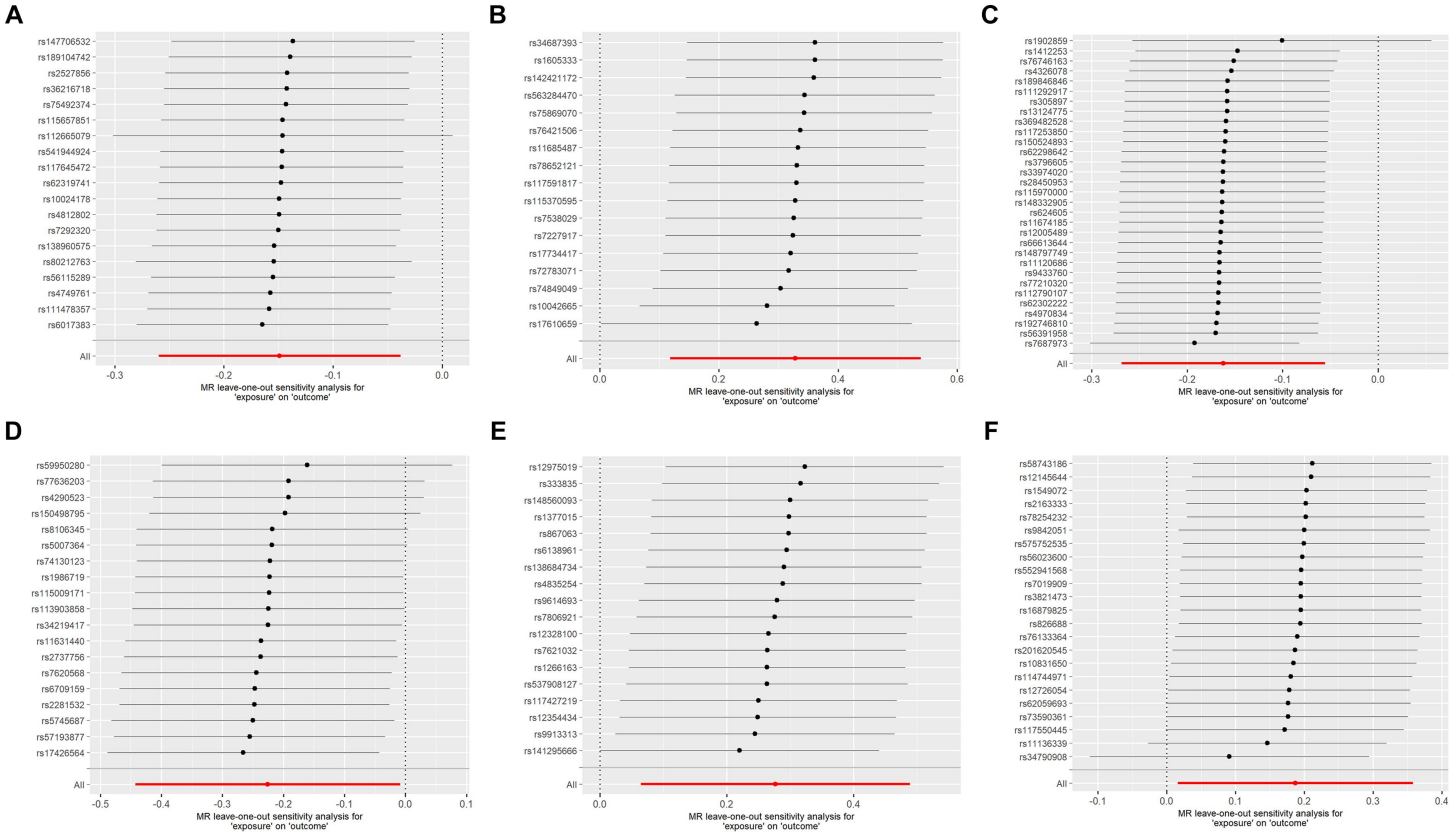
Analysis diagram of leave-one-out method: **(A–F)**: ADA, CSF1, FGF5, HGF, TNFα, and TWEAK.

To assess the robustness of our findings, sensitivity analyses were performed using MR-Egger regression and Cochran’s Q test. The results demonstrate the absence of heterogeneity and pleiotropy in this study, as presented in Table 2. Furthermore, leave-one-out analysis indicates the consistent nature of the results even after systematically excluding individual SNPs, as illustrated in the figure. This consistency aligns with the outcomes of the earlier MR-PRESSO analysis. Collectively, these methodologies offer compelling evidence supporting the robustness of the study results. The diagram of leave-one-out analysis method for those six aforementioned proteins is illustrated in Figure 4.

**Table 2:** Pleiotropy and heterogeneity test for 91 inflammatory cytokines on knee osteoarthritis.

Proteins	Pleiotropy Test			Heterogeneity Test Cochran’ Q			
	MR-Egger-intercept	MR-Egger_P	MR-PRESSO_P	MR Egger_Q	MR Egger_P	IVW_Q	IVW_ P
ADA	−0.006	0.740	0.632	16.988	0.455	17.102	0.516
CSF1	0.021	0.477	0.617	14.338	0.500	14.870	0.534
FGF5	0.010	0.519	0.658	25.721	0.640	26.147	0.668
HGF	0.010	0.694	0.745	13.224	0.721	13.384	0.768
TNF	−0.003	0.885	0.561	15.528	0.486	15.550	0.556
TWEAK	−0.008	0.720	0.458	21.955	0.402	22.093	0.454

## 4 Discussion

Recent studies have demonstrated a close association between the occurrence and progression of KOA and inflammatory cytokines (23). Therefore, in this two-sample MR analysis, we screened 91 inflammatory factors for potential causal associations with KOA, aiming to comprehensively identify factors closely related to the disease. The results indicate that ADA, FGF5, and HGF may serve as potential protective factors against KOA, while TNF, CSF1, and TWEAK may be considered as risk factors for KOA. Following this, we will endeavor to elucidate the potential mechanisms through which each factor may operate within the context of KOA.

Studies have conducted proteomic analysis on synovial fluid proteins in patients with psoriatic arthritis (PsA) and observed significant changes in the expression of ADA protein levels in synovial fluid with the progression of arthritis, suggesting a potential involvement of ADA protein in the immune processes of arthritis (36). A multi-omics study of serum ADA activity in rheumatoid arthritis patients found elevated levels of TNFα, IFN γ, and IL-10 in patients with hyperactive ADA, exacerbating inflammation, promoting osteoclast differentiation, and negatively impacting bone metabolism (37, 38). However, there is also research supporting the viewpoint that ADA protein acts as a protective factor in KOA. ADA is a key enzyme in purine metabolism, converting adenosine to inosine through its deaminase activity (39). In a mouse model of osteoarthritis, Mistry et al. observed that MC615 chondrocytes treated with adenosine exhibited significant apoptosis (40), suggesting cytotoxicity due to excessive intracellular adenosine levels (41). This apoptotic phenomenon was similarly observed in equine chondrocytes (42). Our study results align with the latter perspective, indicating ADA as a protective factor in KOA. However, the underlying mechanisms require further in-depth investigation for clarification.

Macrophage Colony-Stimulating Factor (M-CSF), also known as CSF1, is a member of the hematopoietic colony-stimulating factor (CSF) family (43). Under normal physiological conditions, M-CSF binds to the CSF1R receptor on the surface of macrophages, facilitating the differentiation of monocytes into mature macrophages and playing a crucial role in immune modulation (43) and tissue repair (44). Basic research has uncovered that synovial fibroblasts in patients with rheumatoid arthritis and osteoarthritis actively produce M-CSF, leading to the differentiation of synovial macrophages into osteoclasts (45). Research has found that TNF-α acts through M-CSF expressed by bone marrow stromal cells and stimulates the expression of the M-CSF gene (46, 47). In animal models, the exacerbation of collagen-induced arthritis (CIA) severity is observed upon the exogenous administration of CSF1. Conversely, the knockout or neutralization of CSF1 demonstrates a distinct therapeutic effect (48–50). Notably, Giordano et al. reported a significant upregulation of CSF1 expression in the serum of KOA patients (51). Linear regression analysis further revealed a positive correlation between CSF1 levels and pain intensity (52). These findings not only position CSF1 as a contributory risk factor in the pathogenesis of KOA but also suggest that reducing CSF1 levels holds promise as a potential therapeutic target for this debilitating condition.

Research indicates that synovial-like fibroblast cells (FLS) from osteoarthritis patients secrete more growth factors, including Fibroblast Growth Factor 5 (FGF5), upon exposure to pro-inflammatory stimuli (53). Clase et al. propose that FGF5, as a mitogenic stimulus for mesenchymal fibroblasts, promotes the proliferation of these fibroblasts, contributing to the formation of connective tissues such as the synovium (54). The findings of our study suggest that FGF5 may serve as a potential protective factor in KOA. Therefore, we hypothesize that FGF5 might delay the progression of osteoarthritis by participating in the repair of the synovium, and further investigations are warranted to validate this assumption.

Hepatocyte growth factor (HGF) is a potent mitogen that plays a key role in the growth and differentiation of various tissues (55, 56). Its anti-inflammatory activity has been reported in several disease animal models and across multiple organ systems (57). Research indicates that HGF significantly induces FLS to secrete chemokine MCP-1, promoting the migration of monocytes and macrophages to sites of inflammation or injury (58). These cells then secrete TGF-β1 and BMP-2, contributing to the formation of osteophytes (58). Adding HGF to *in vitro* cultures of primary chondrocytes from rabbits and rats leads to a substantial increase in proteoglycan synthesis and cell proliferation rates, demonstrating its role in supporting articular cartilage repair (59). Mohammad A. J. and colleagues (60) found that HGF works in conjunction with TGF-β and IDO, immune-regulatory enzymes, to enhance the immunosuppressive properties of human mesenchymal stem cells (MSCs). This results in a reduction in the infiltration of inflammatory cells and supports tissue repair and regeneration. This is consistent with the findings of our study, suggesting that HGF is a protective factor in KOA. HGF may potentially delay disease progression by participating in the regulation and repair of articular cartilage homeostasis.

Tumor Necrosis Factor-α (TNFα) is a common inflammatory factor and a crucial participant in osteoarthritis. Terkeltaub R’s team (61) reported that their studies on primary chondrocytes from humans and mice revealed that TNFα can induce the production of matrix metalloproteinases (MMPs), prostaglandin E2 (PGE2), and nitric oxide (NO), leading to a decrease in chondrocytes and joint cartilage destruction. The infrapatellar fat pad can also produce TNF-α, and this autocrine effect plays an important role in the pathological changes seen in KOA (62). Studies have shown a significant increase in TNF-α expression in tissues from OA patients compared to normal subjects, and elevated levels of TNF-α in joint fluid may accelerate the progression of OA (12). A meta-analysis on TNF-α gene polymorphism and the risk of KOA indicated a close association between TNF-α expression levels and genetic polymorphism. Patients carrying alleles such as TNF-α-G308A exhibited higher TNF-α levels and a heightened risk of developing osteoarthritis (63). Clinical research indicates that targeting TNFα with adalimumab (64) and natural extracts such as curcumin (65) and Aflapin (66) can provide safe and effective relief from pain and other symptoms associated with KOA. This aligns with the results of our study: TNFα is a risk factor for KOA. Strategies to reduce TNF expression levels may be a potential avenue for the treatment of this condition.

Tumor necrosis factor ligand superfamily member 12, also known as tumor necrosis factor-like weak inducer of apoptosis (TWEAK), is associated with inflammation and plays a role in immune regulation, cell apoptosis, and tissue recovery and reconstruction before the onset of inflammation (67). Research has revealed that upon binding to its receptor Fn14, TWEAK induces the generation of matrix metalloproteinase-1 (MMP1) (68, 69). While MMP-1 maintains low expression in normal cells and promotes the regeneration of healthy cartilage, pathological conditions can lead to its overexpression. This excess expression results in the degradation of type I, II, and III fibers in the extracellular matrix, facilitating cartilage destruction and exacerbating the progression of OA (70). Additionally, TWEAK is believed to inhibit cartilage and bone formation, further intensifying the likelihood of cartilage degradation (71). Hwang et al. assessed TWEAK levels in joint fluid from patients with different stages of OA (72). They highlighted that high levels of TWEAK are linked to pro-inflammatory responses and accelerated cartilage destruction, particularly during the early stages of the disease. This interaction may be facilitated by the relationship between TWEAK and MMP1. Therefore, as a relevant risk factor for KOA, the expression levels of TWEAK can serve as an early screening marker for this condition.

In this investigation, we utilized Mendelian randomization to evaluate the causal association between KOA and 91 inflammatory cytokines. Regarding the interactions among the few factors mentioned in the article, we conducted further analysis to explore potential mediating effects. The results continue to support our original conclusions. (Refer to the Supplementary material for details) However, several limitations need consideration. Firstly, when selecting an SNP filtering threshold, a stringent threshold may lead to an insufficient number of SNPs available for analysis, resulting in inaccurate estimates of causal effects. Therefore, we opted for a more lenient threshold, which increased the number of SNPs included in the analysis within a certain range. However, this may introduce weak instrumental variables and increase interference from confounding factors and horizontal pleiotropy. To address this, we filtered out weak instrumental variables based on the *F*-value calculation and conducted sensitivity tests to exclude horizontal pleiotropy. We adopted a relatively strict confounder removal standard, though there may still be omissions, especially when SNPs are associated with unreported confounding factors. Secondly, our survey data originated from two extensive GWAS, and the absence of specific demographic information and clinical records for study patients hindered subgroup analysis. Thirdly, caution is warranted regarding potential racial bias, as the subjects exclusively belonged to European descent, limiting the generalizability of results to other ethnicities. Further studies are crucial to validate our findings and explore their applicability to clinical diagnostic procedures and treatment options.

## Data availability statement

The original contributions presented in the study are included in the article/Supplementary materials, further inquiries can be directed to the corresponding authors.

## Ethics statement

Ethical approval was not required for the study involving humans in accordance with the local legislation and institutional requirements. Written informed consent to participate in this study was not required from the participants or the participants' legal guardians/next of kin in accordance with the national legislation and the institutional requirements.

## Author contributions

LX: Writing – original draft, Writing – review & editing. JM: Software, Writing – review & editing. QY: Writing – review & editing. KZ: Writing – review & editing. XW: Writing – review & editing. CZ: Funding acquisition, Writing – review & editing, Writing – original draft. XL: Funding acquisition, Writing – review & editing, Writing – original draft.

## Footnotes

^1^ http://www.phenoscanner.medschl.cam.ac.uk/upload/
