# Supplementary figures and images for: Evidence supported by Mendelian randomization: impact on inflammatory factors in knee osteoarthritis
Source: Front Med (Lausanne). 2024 May 28;11:1382836. doi: 10.3389/fmed.2024.1382836 (PMC11165061; doi:10.3389/fmed.2024.1382836)

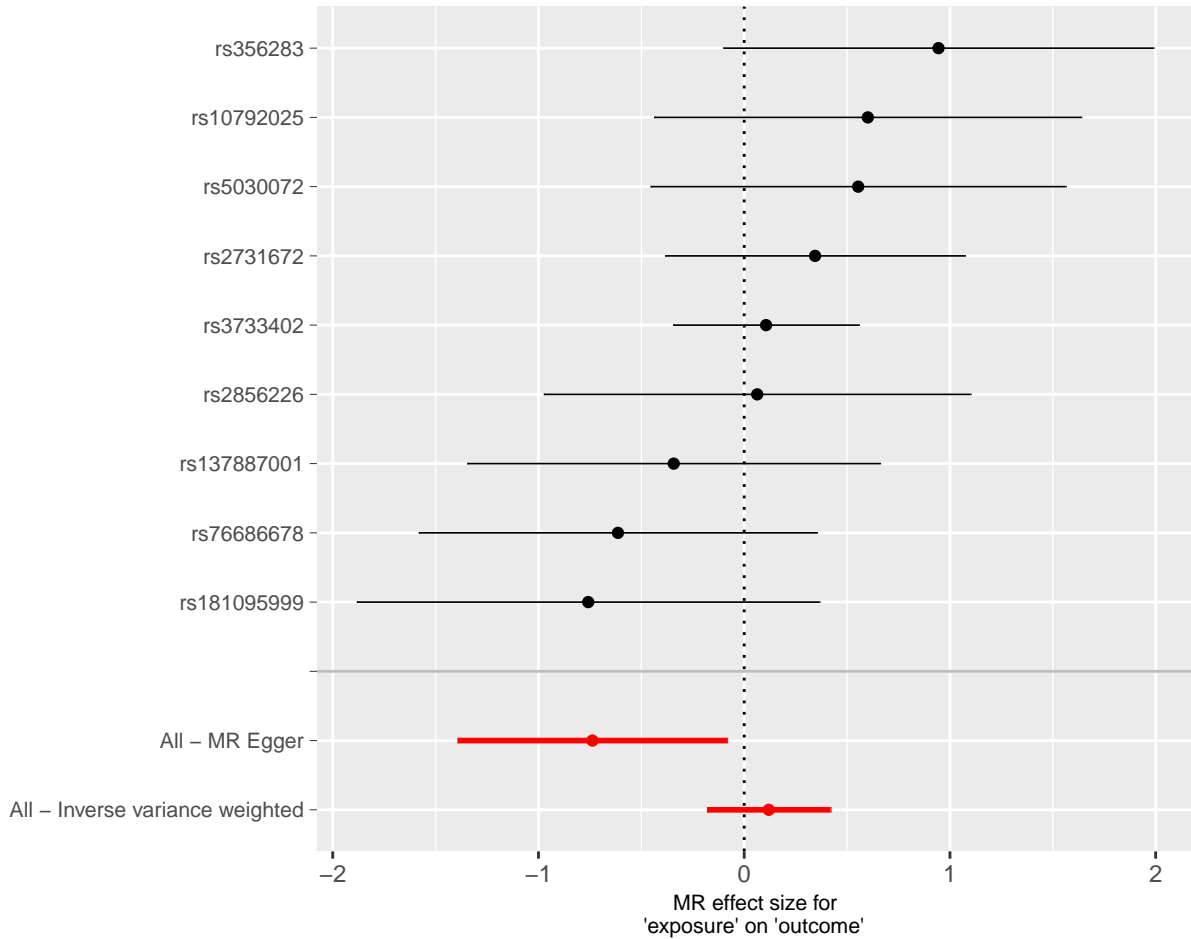

Supplement: Supplementary file 1 [file Data_Sheet_1.ZIP › data/GCST90274758_5e6/758.forest.pdf]

# MR Method

- Inverse variance weighted
- MR Egger

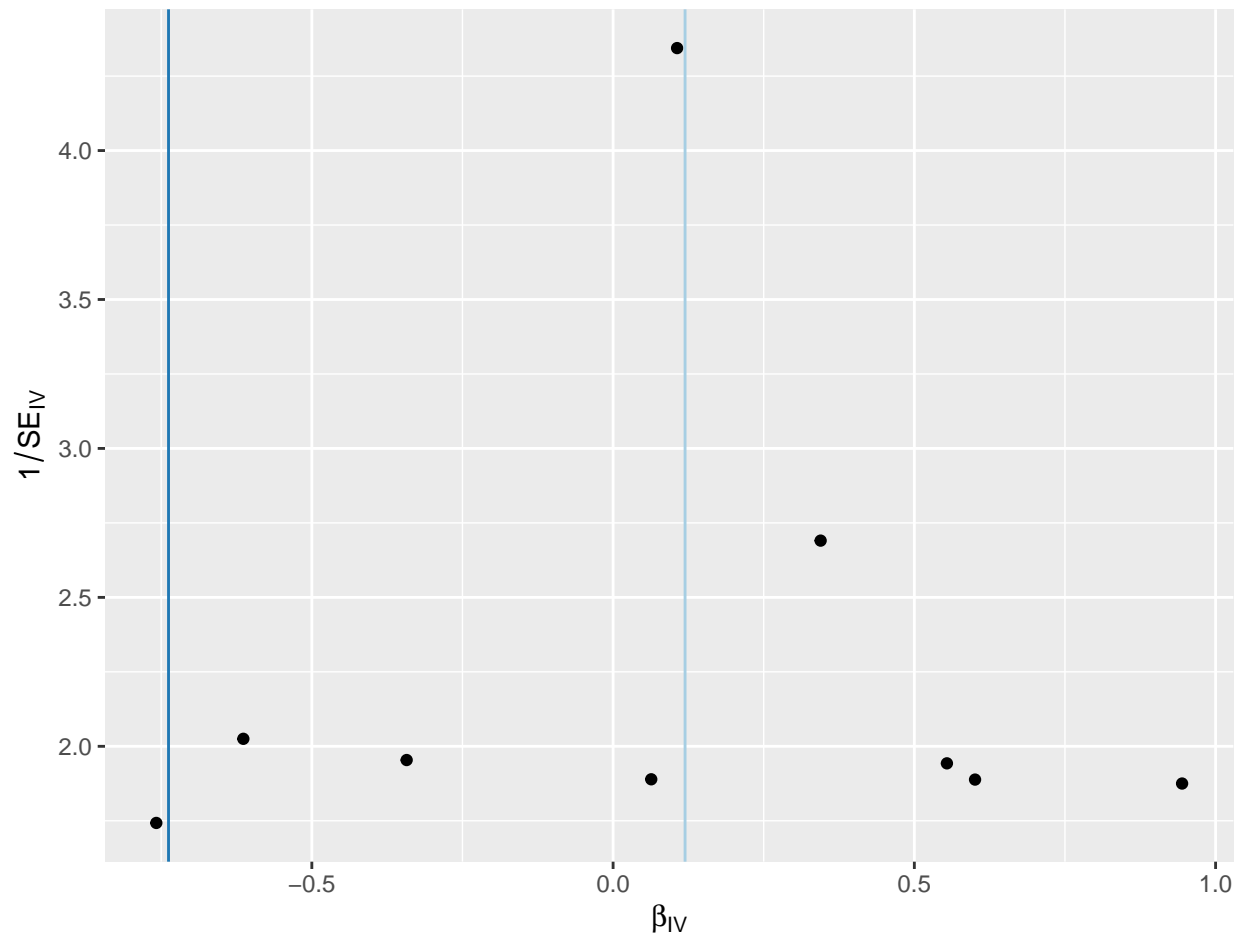

Supplement: Supplementary file 1 [file Data_Sheet_1.ZIP › data/GCST90274758_5e6/758.funnel_plot.pdf]

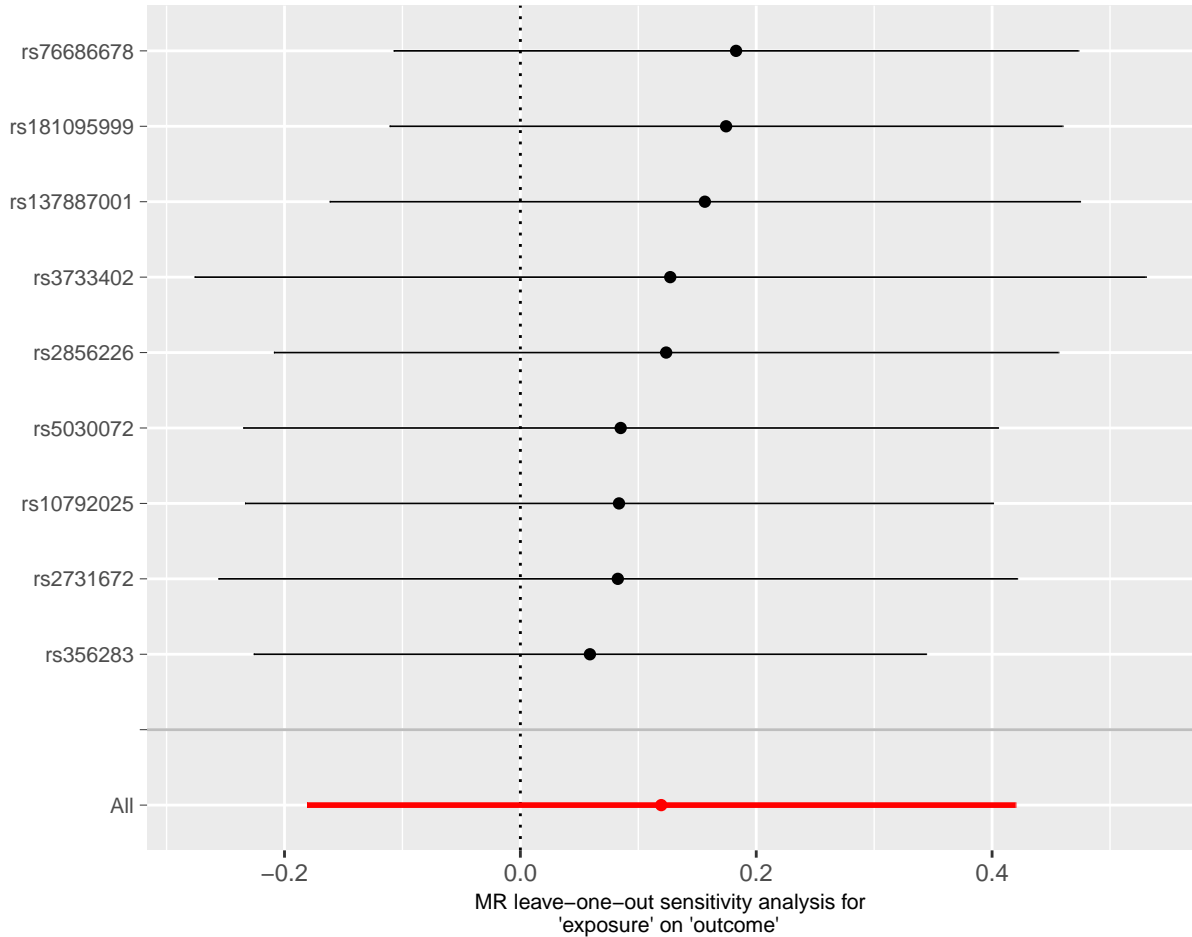

Supplement: Supplementary file 1 [file Data_Sheet_1.ZIP › data/GCST90274758_5e6/758.leaveoneout.pdf]

# MR Test

- Inverse variance weighted
- MR Egger
- Simple mode
- Weighted median
- Weighted mode

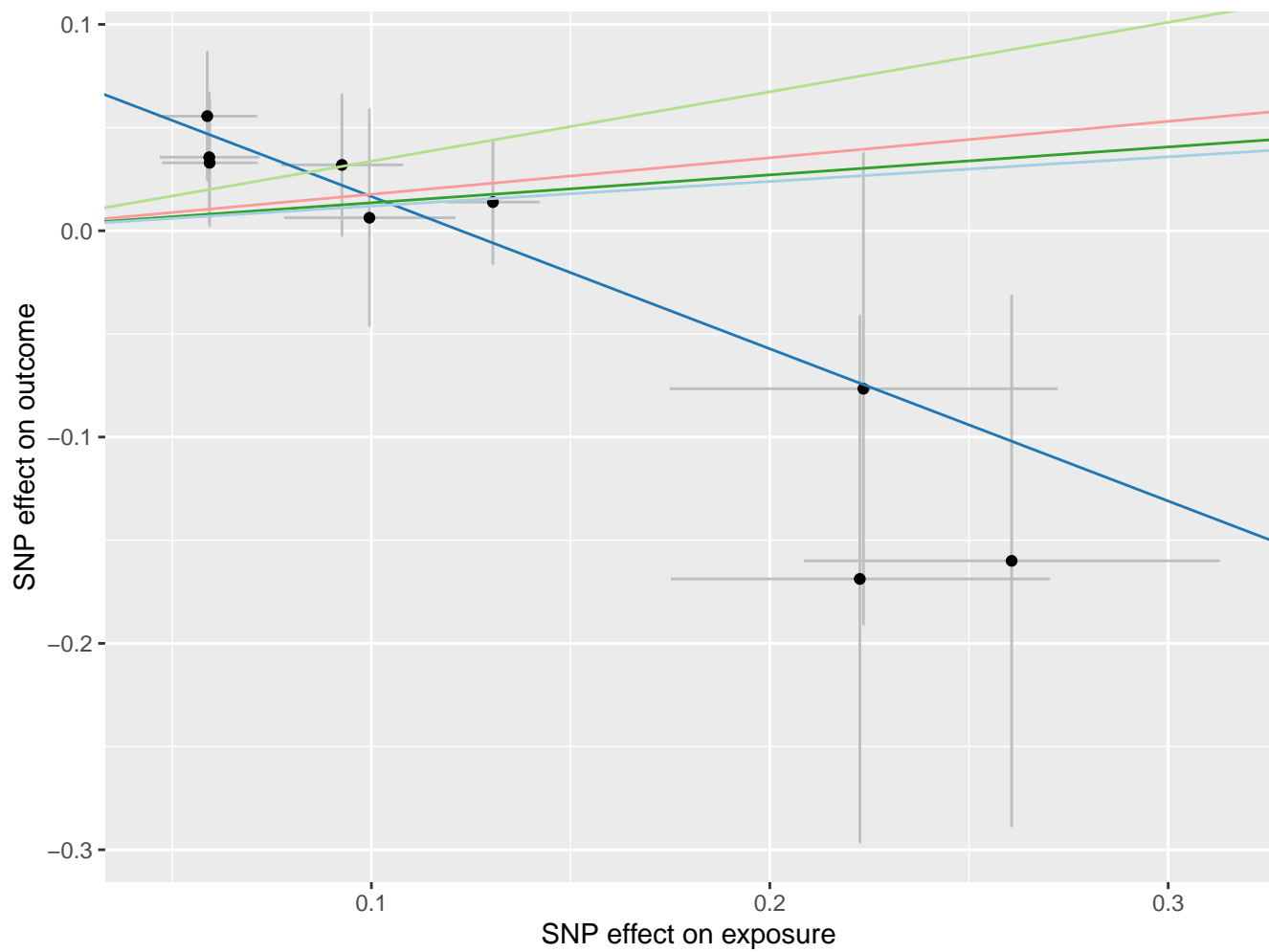

Supplement: Supplementary file 1 [file Data_Sheet_1.ZIP › data/GCST90274758_5e6/758.scatter_plot.pdf]

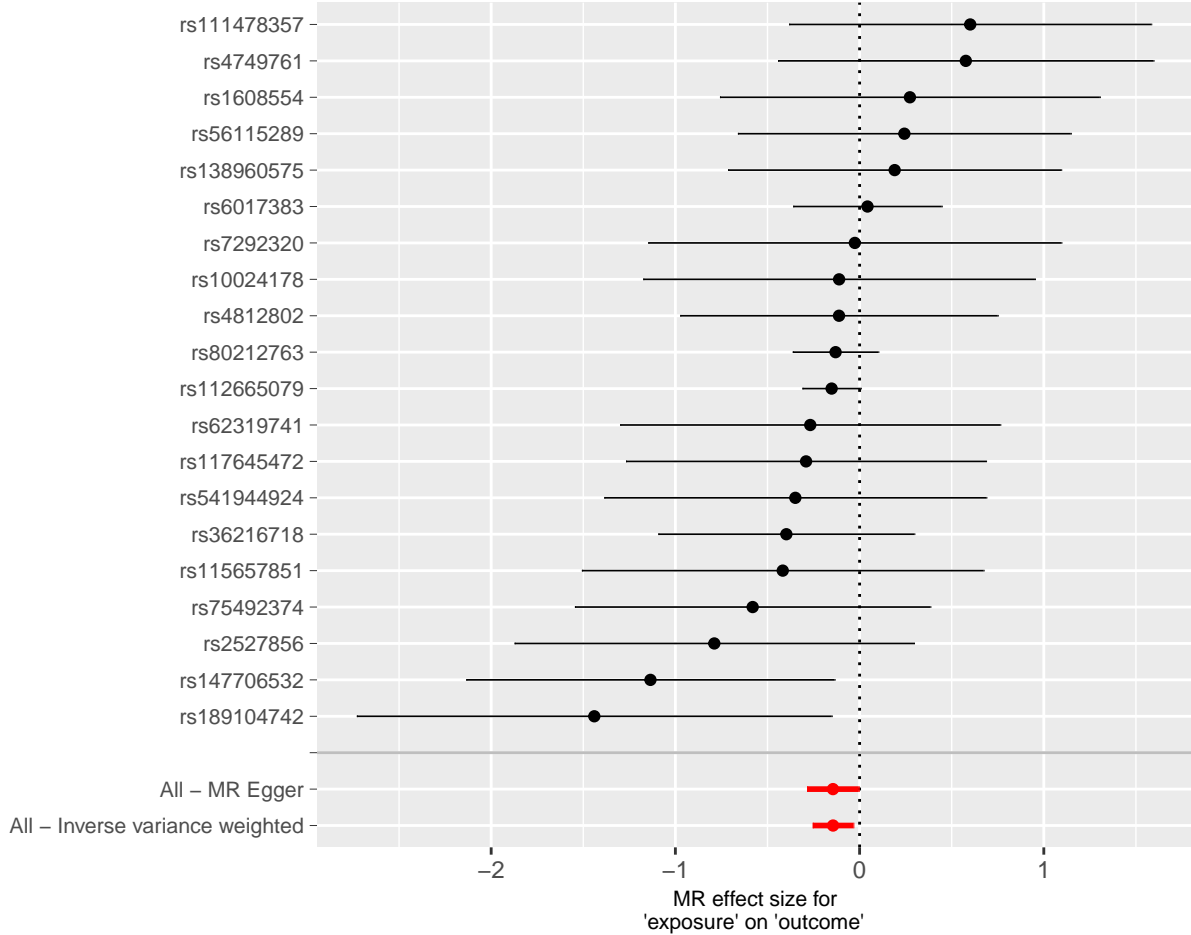

Supplement: Supplementary file 1 [file Data_Sheet_1.ZIP › data/GCST90274759_5e6/759.forest.pdf]

# MR Method

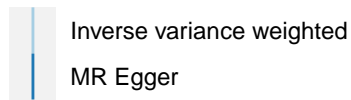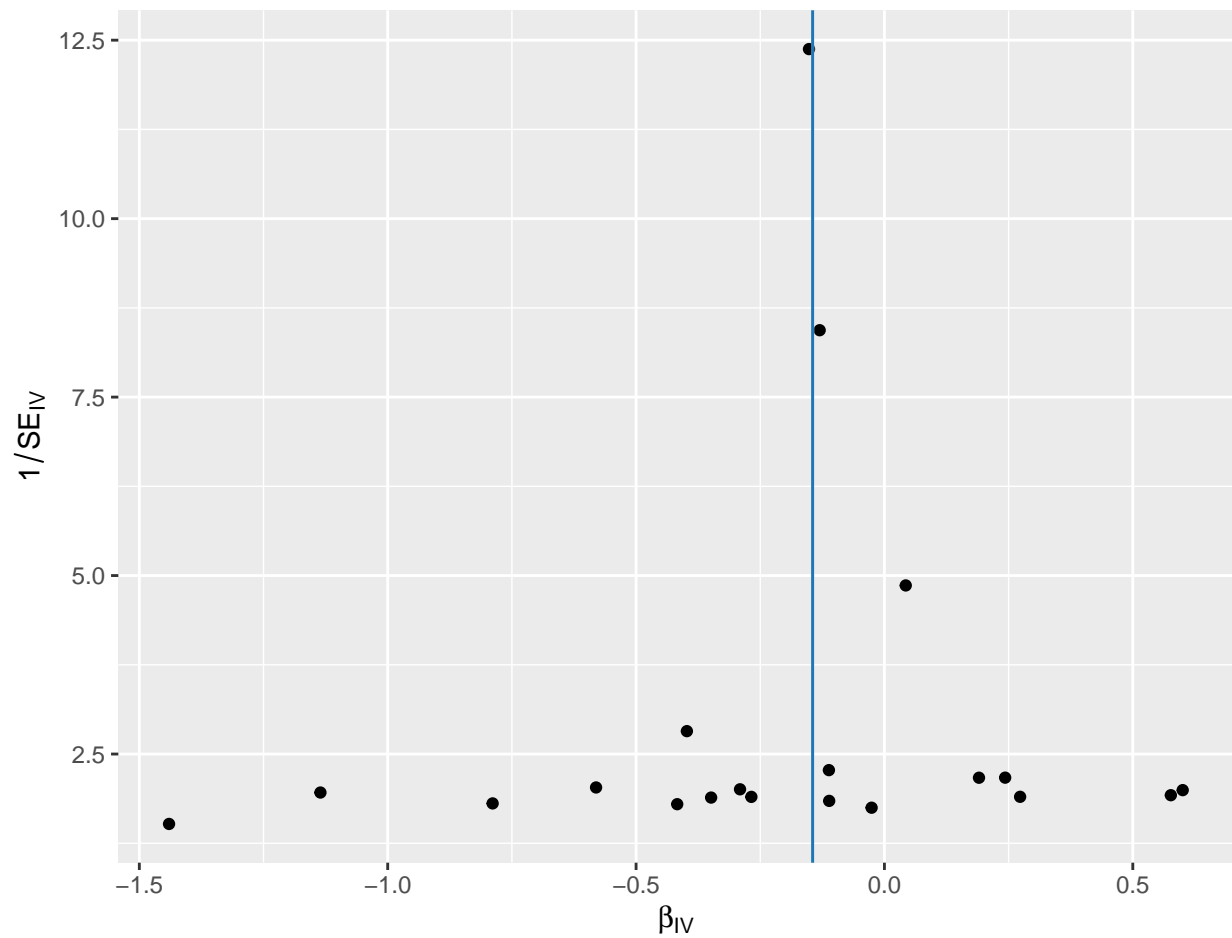

Supplement: Supplementary file 1 [file Data_Sheet_1.ZIP › data/GCST90274759_5e6/759.funnel_plot.pdf]

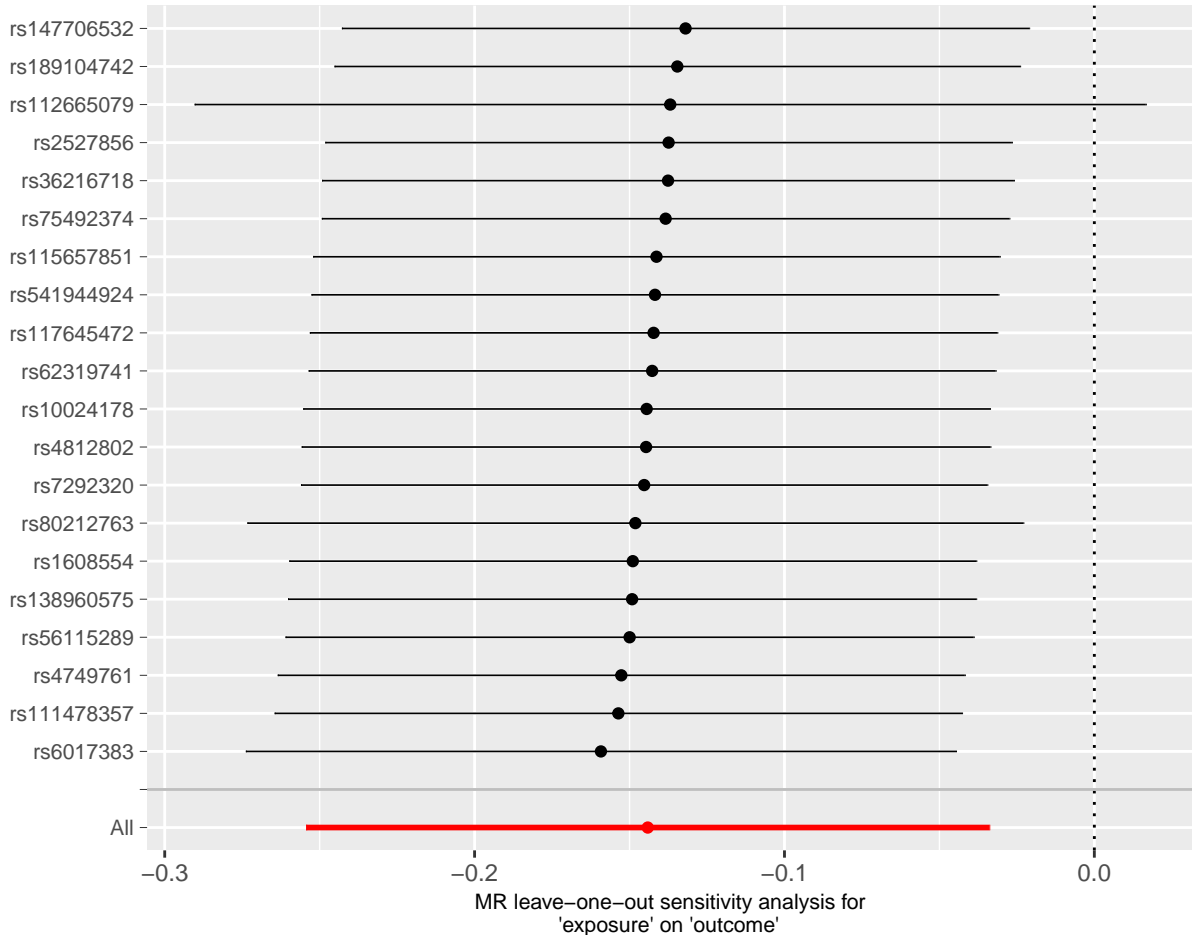

Supplement: Supplementary file 1 [file Data_Sheet_1.ZIP › data/GCST90274759_5e6/759.leaveoneout.pdf]

# MR Test

- Inverse variance weighted
- MR Egger
- Simple mode
- Weighted median
- Weighted mode

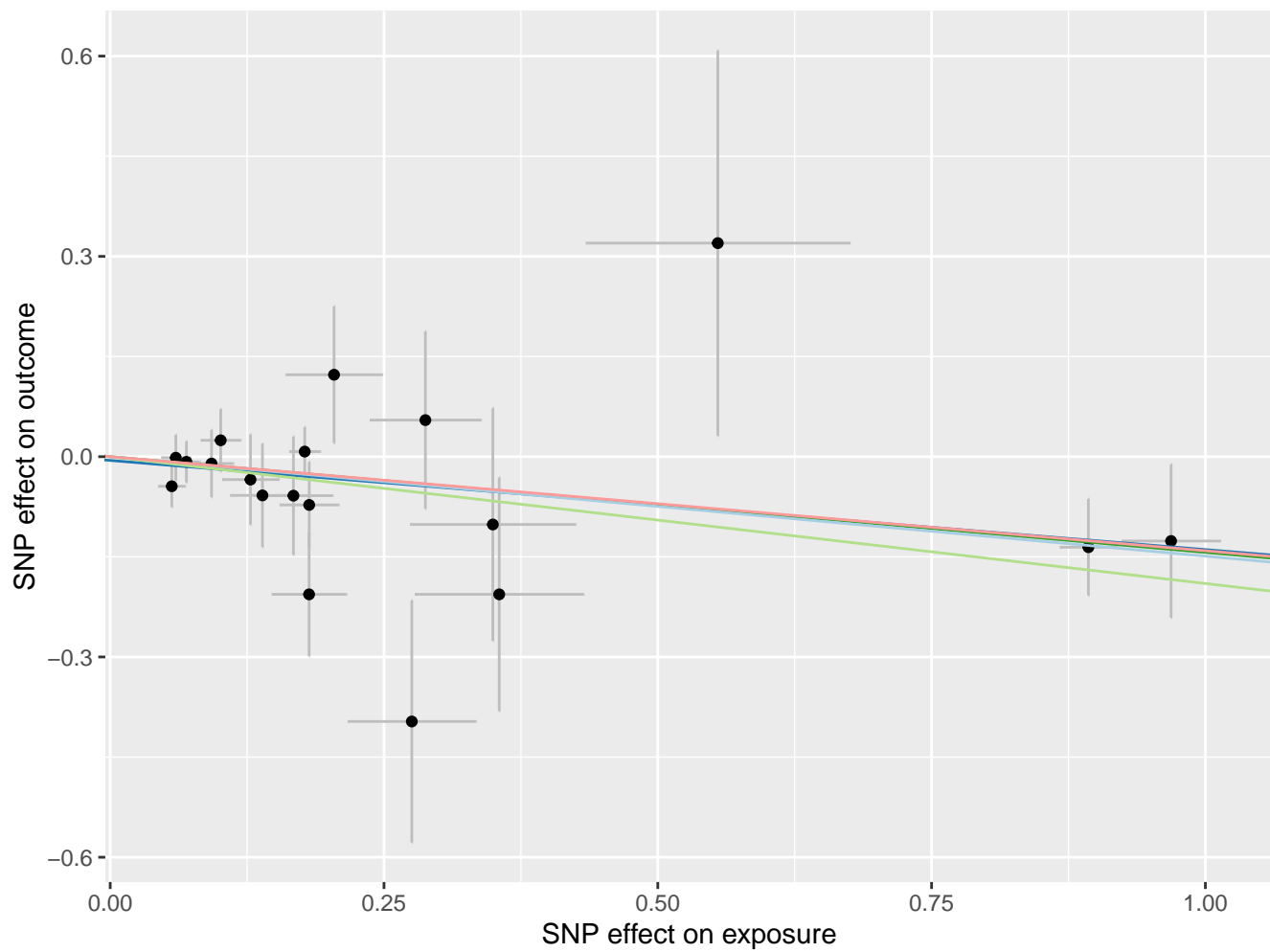

Supplement: Supplementary file 1 [file Data_Sheet_1.ZIP › data/GCST90274759_5e6/759.scatter_plot.pdf]

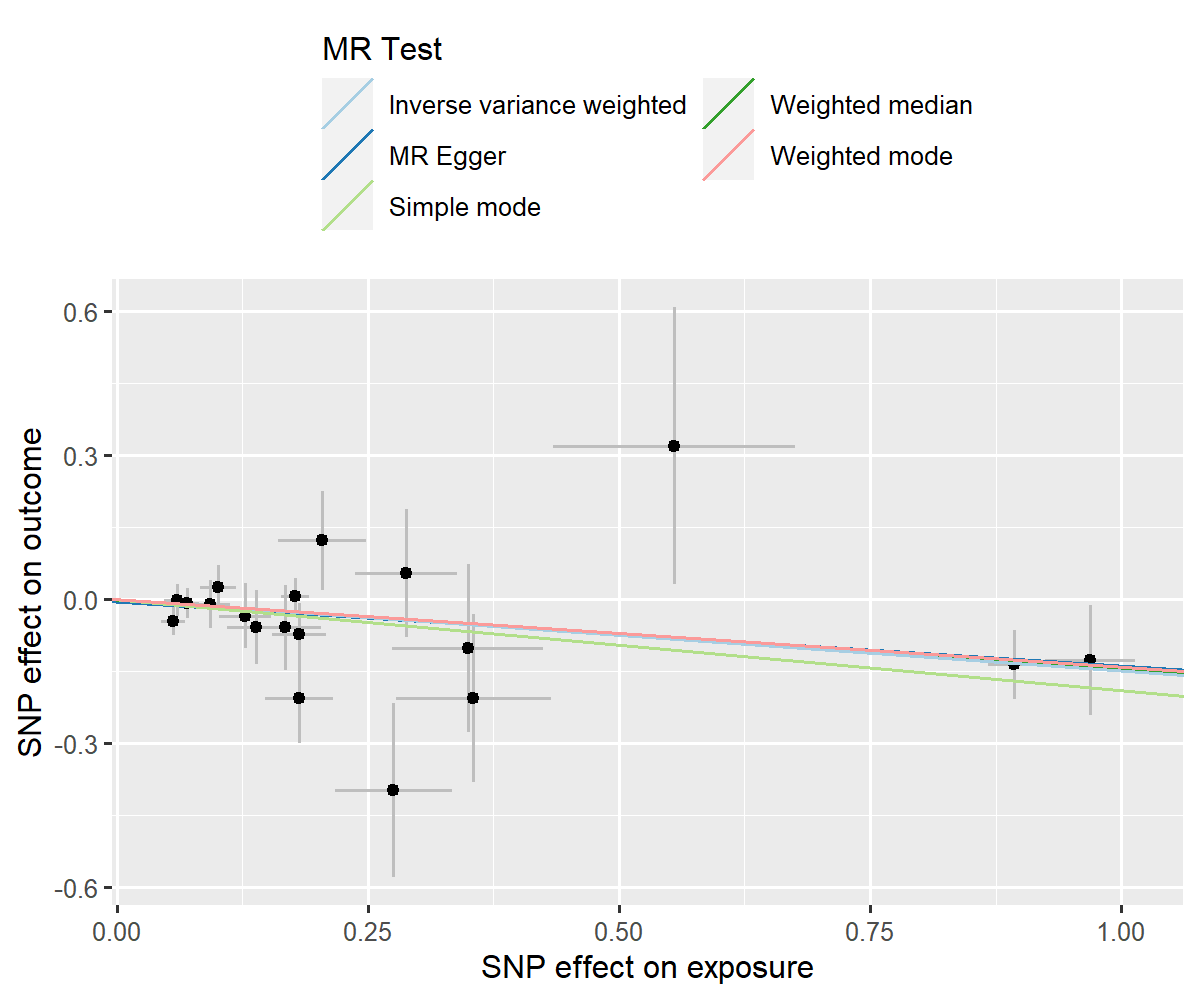

Supplement: Supplementary file 1 [file Data_Sheet_1.ZIP › data/GCST90274759_5e6/759.scatter_plot.png]

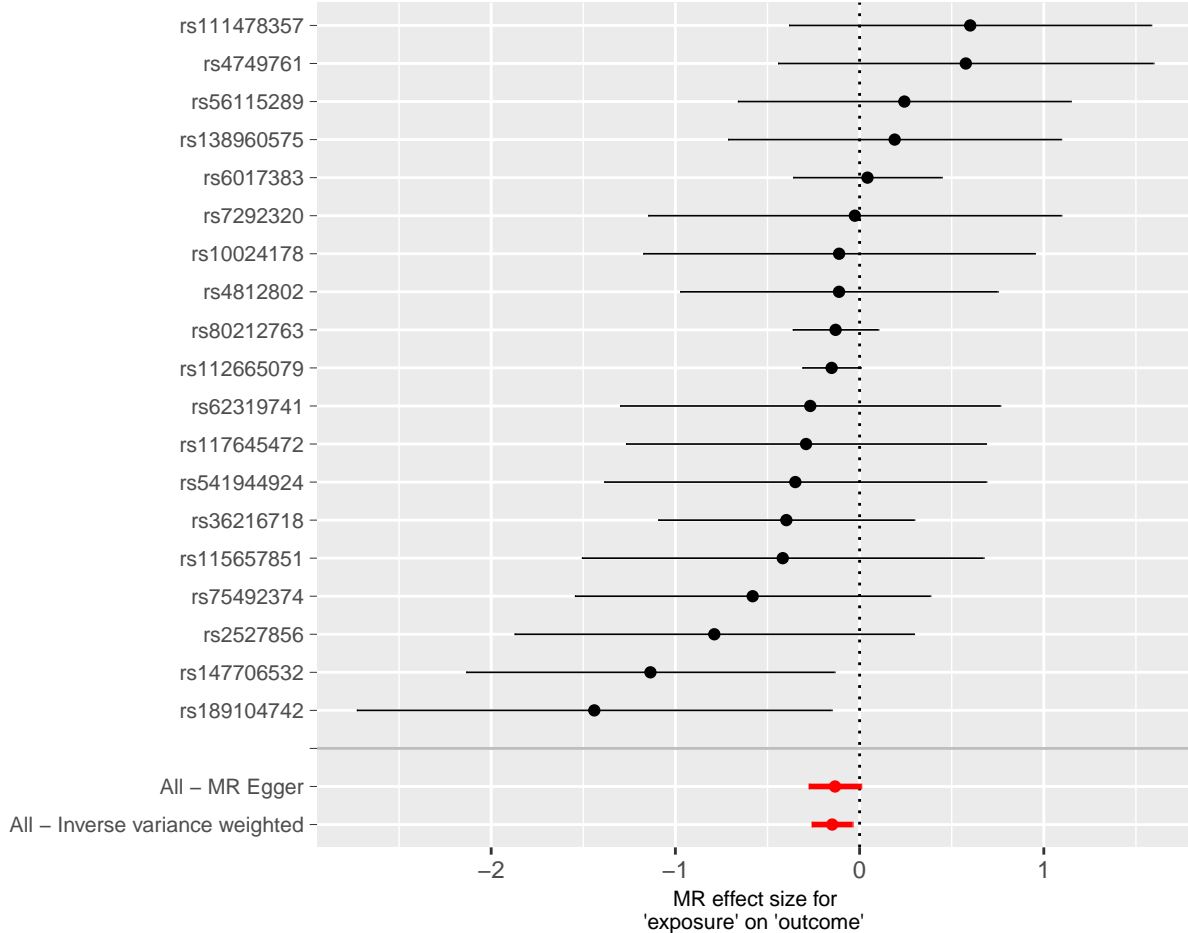

Supplement: Supplementary file 1 [file Data_Sheet_1.ZIP › data/GCST90274759_5e6/again/759.forest.pdf]

# MR Method

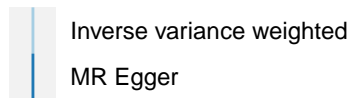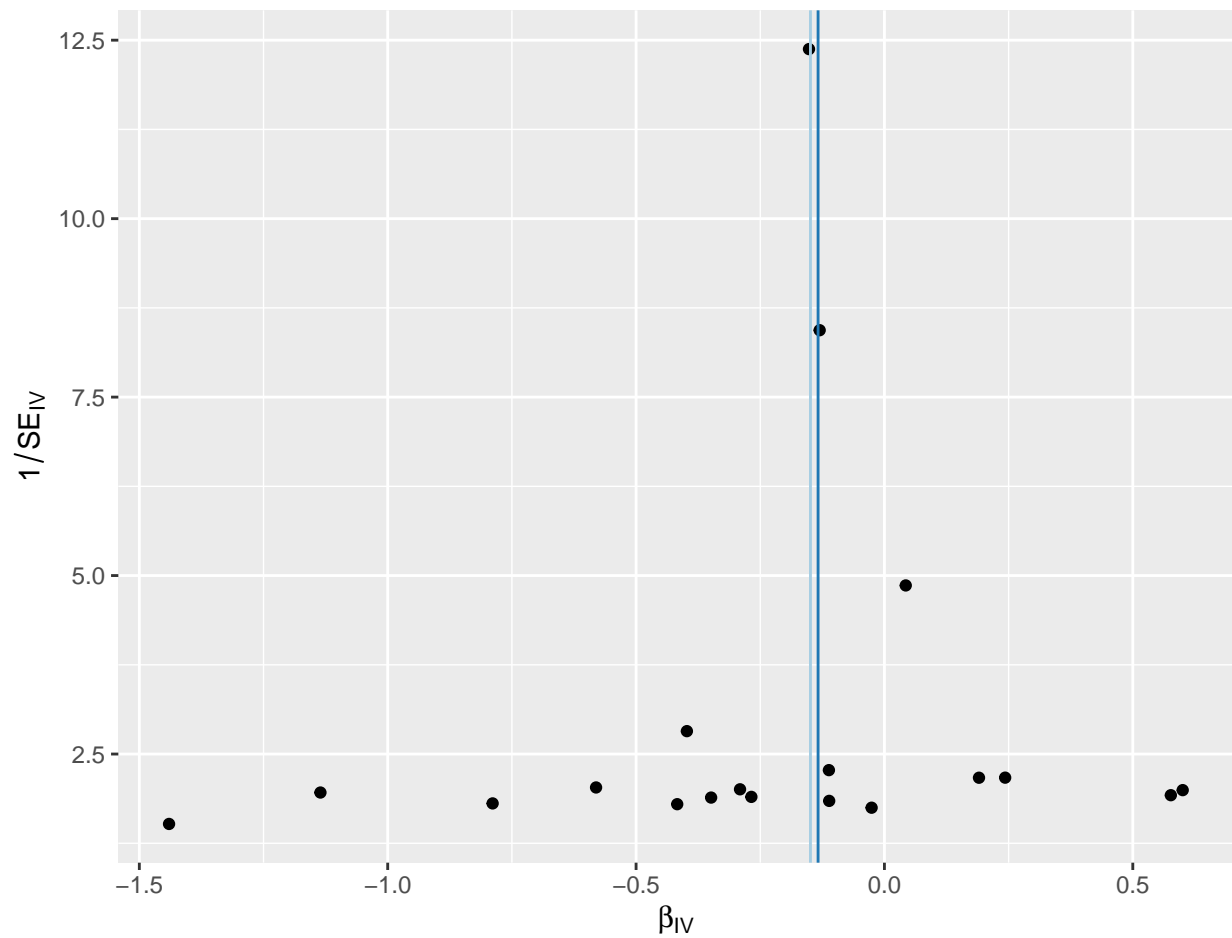

Supplement: Supplementary file 1 [file Data_Sheet_1.ZIP › data/GCST90274759_5e6/again/759.funnel_plot.pdf]

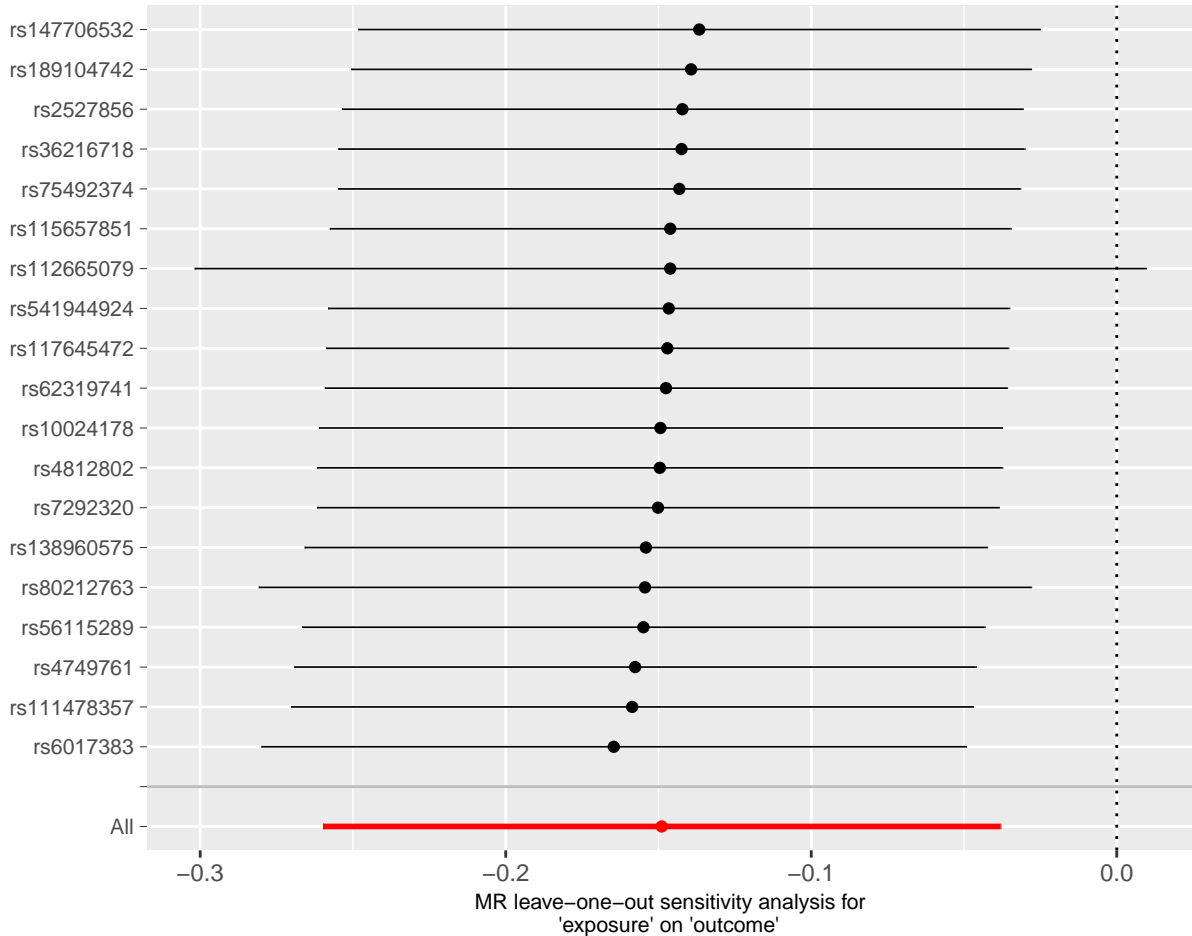

Supplement: Supplementary file 1 [file Data_Sheet_1.ZIP › data/GCST90274759_5e6/again/759.leaveoneout.pdf]

# MR Test

- Inverse variance weighted
- MR Egger
- Simple mode
- Weighted median
- Weighted mode

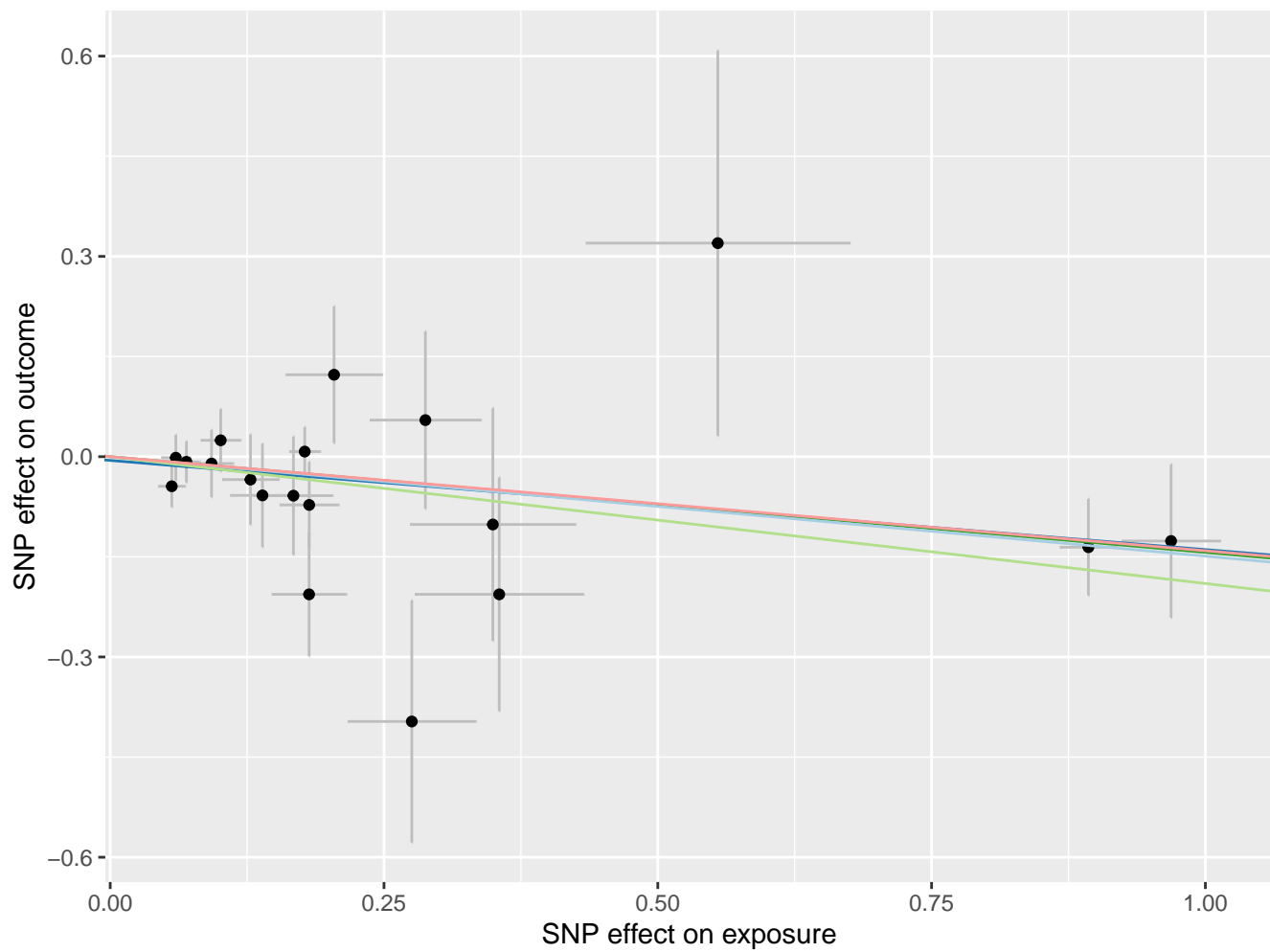

Supplement: Supplementary file 1 [file Data_Sheet_1.ZIP › data/GCST90274759_5e6/again/759.scatter_plot.pdf]

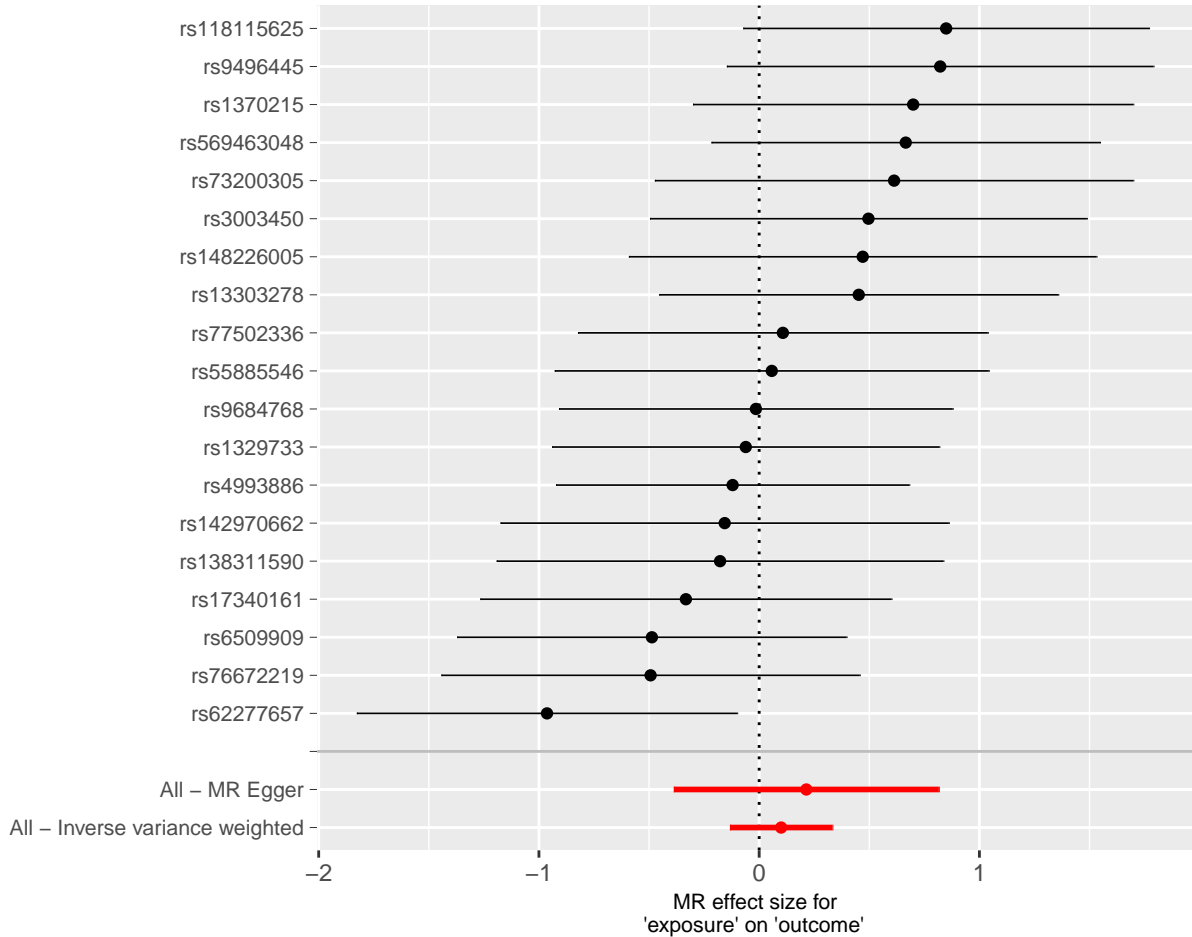

Supplement: Supplementary file 1 [file Data_Sheet_1.ZIP › data/GCST90274760_5e6/760.forest.pdf]

# MR Method

- Inverse variance weighted
- MR Egger

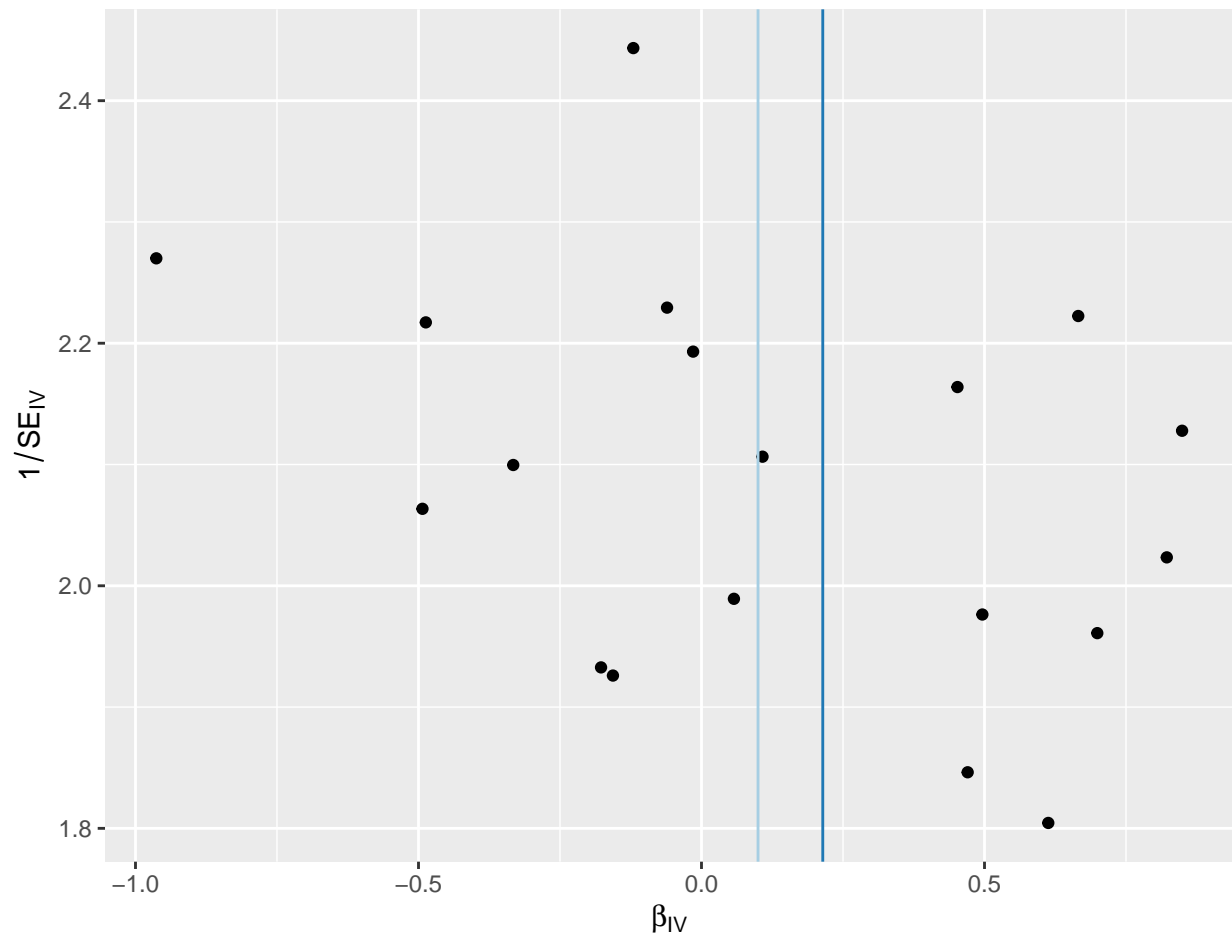

Supplement: Supplementary file 1 [file Data_Sheet_1.ZIP › data/GCST90274760_5e6/760.funnel_plot.pdf]

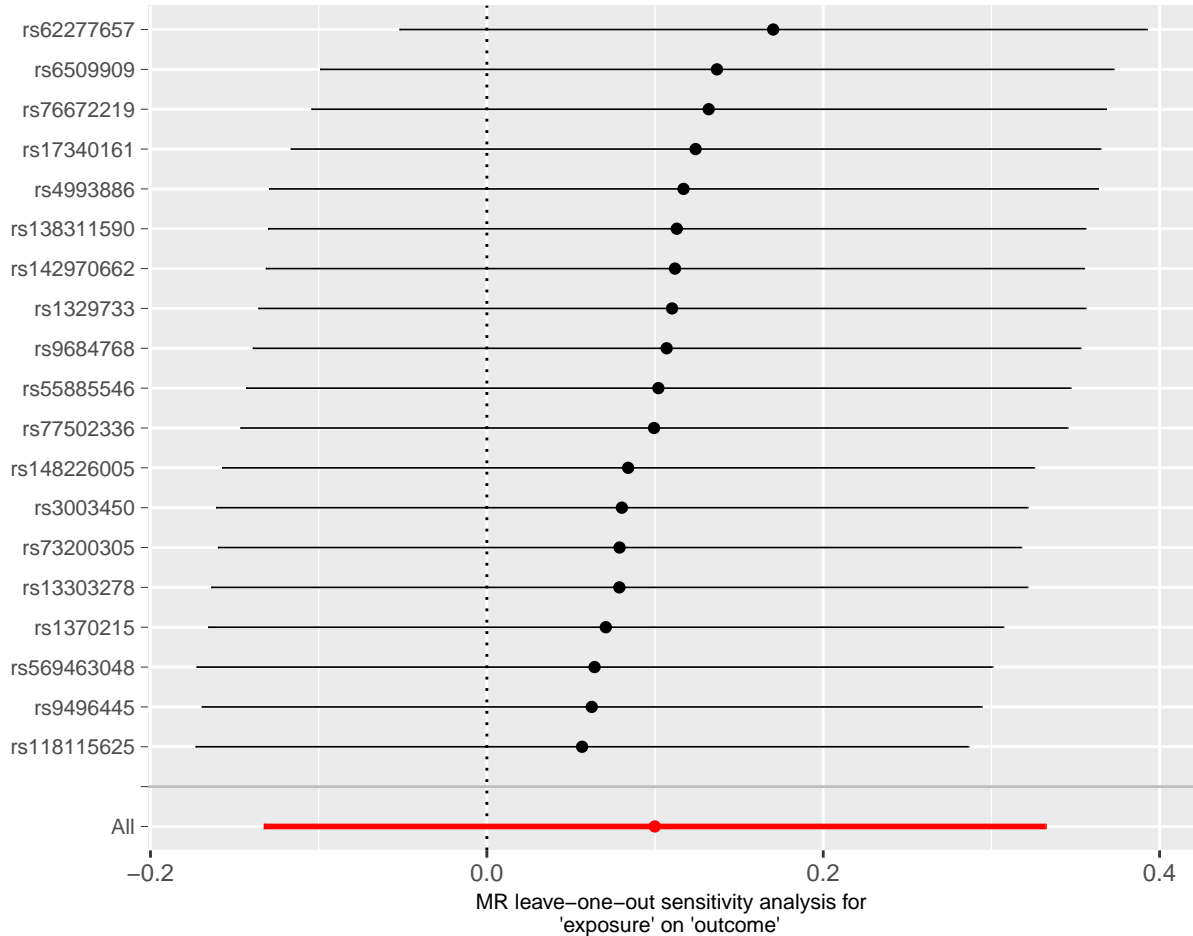

Supplement: Supplementary file 1 [file Data_Sheet_1.ZIP › data/GCST90274760_5e6/760.leaveoneout.pdf]

# MR Test

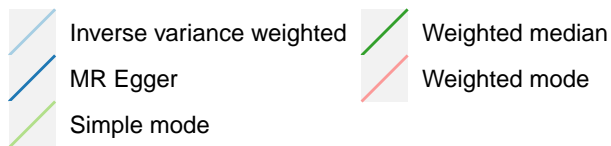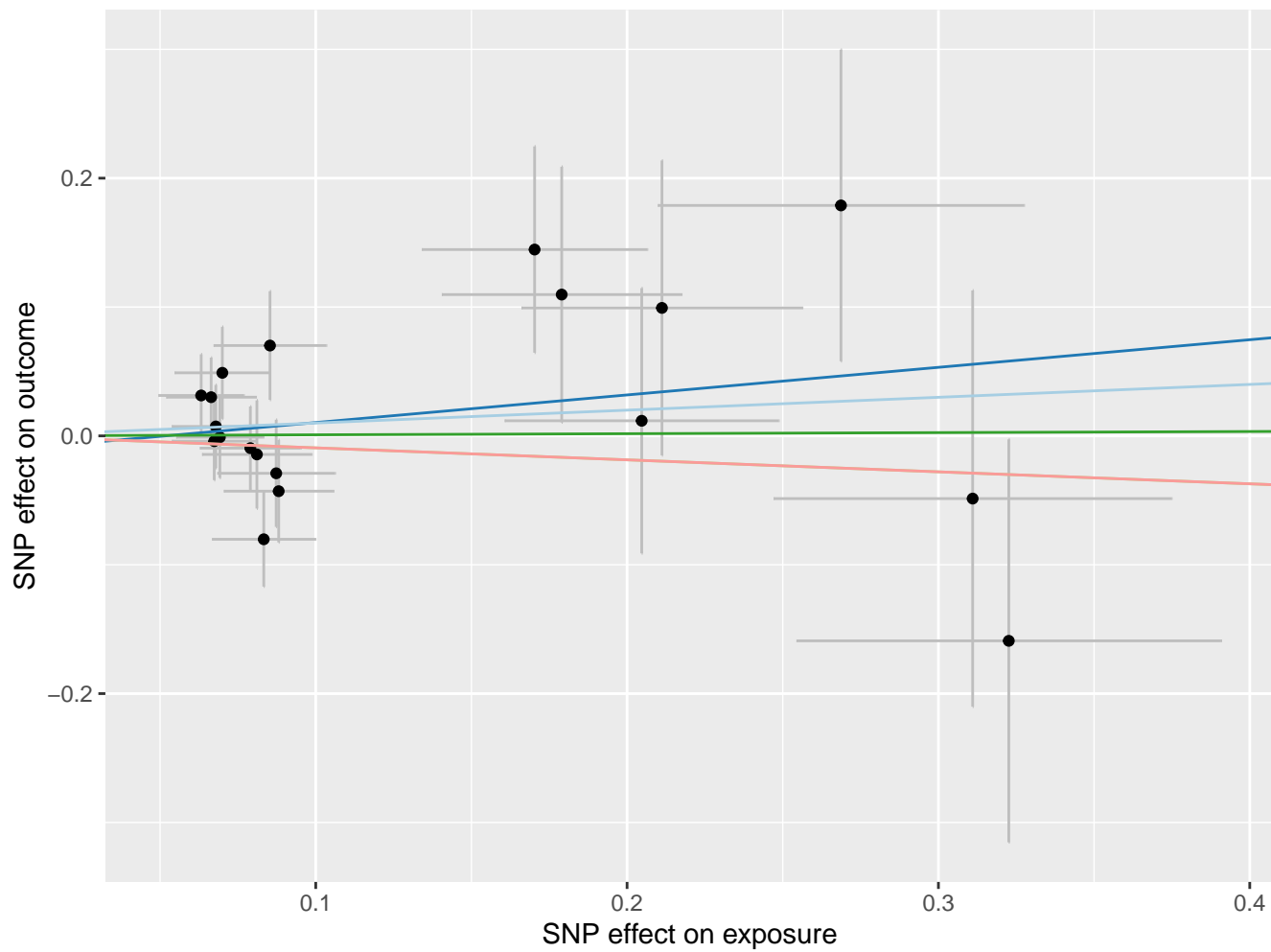

Supplement: Supplementary file 1 [file Data_Sheet_1.ZIP › data/GCST90274760_5e6/760.scatter_plot.pdf]

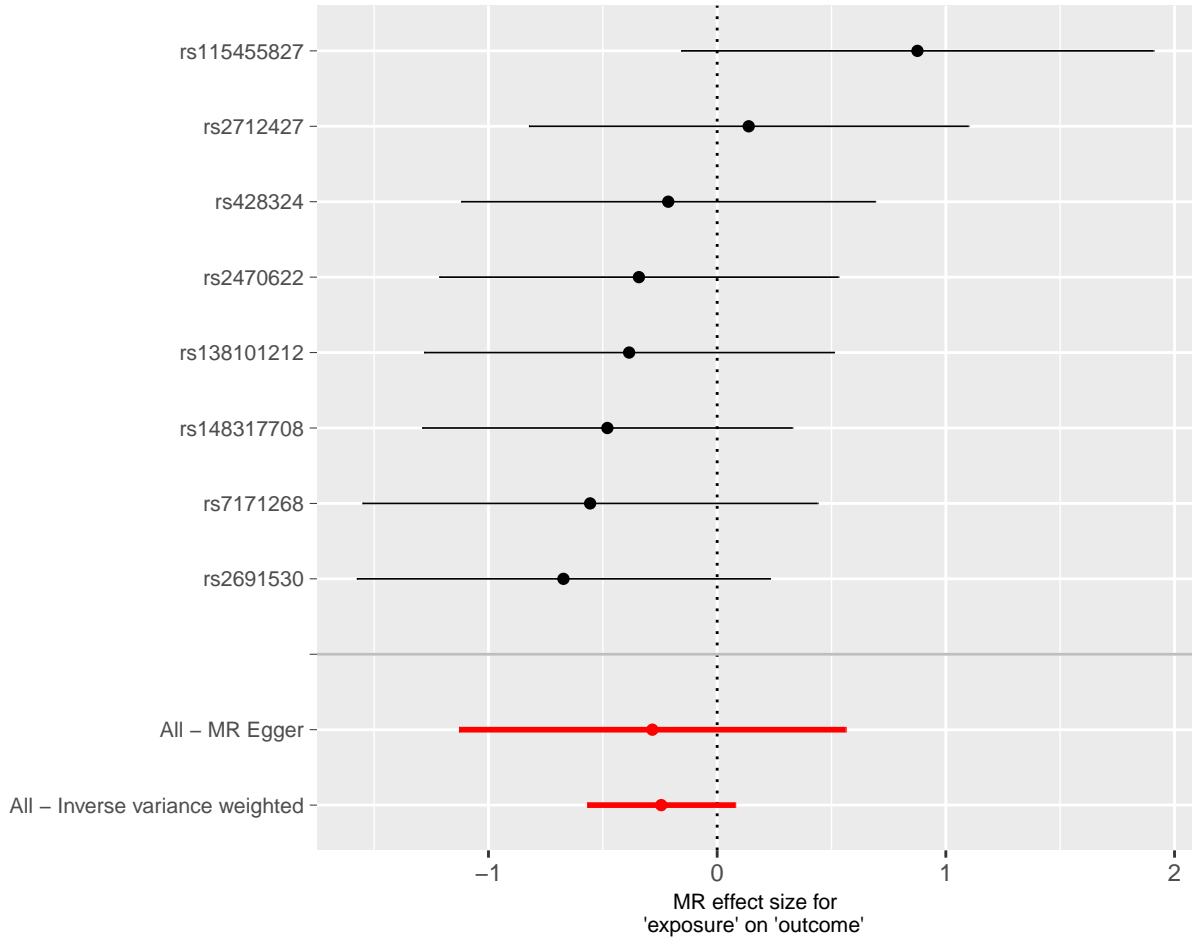

Supplement: Supplementary file 1 [file Data_Sheet_1.ZIP › data/GCST90274761_5e6/761.forest.pdf]

# MR Method

- Inverse variance weighted
- MR Egger

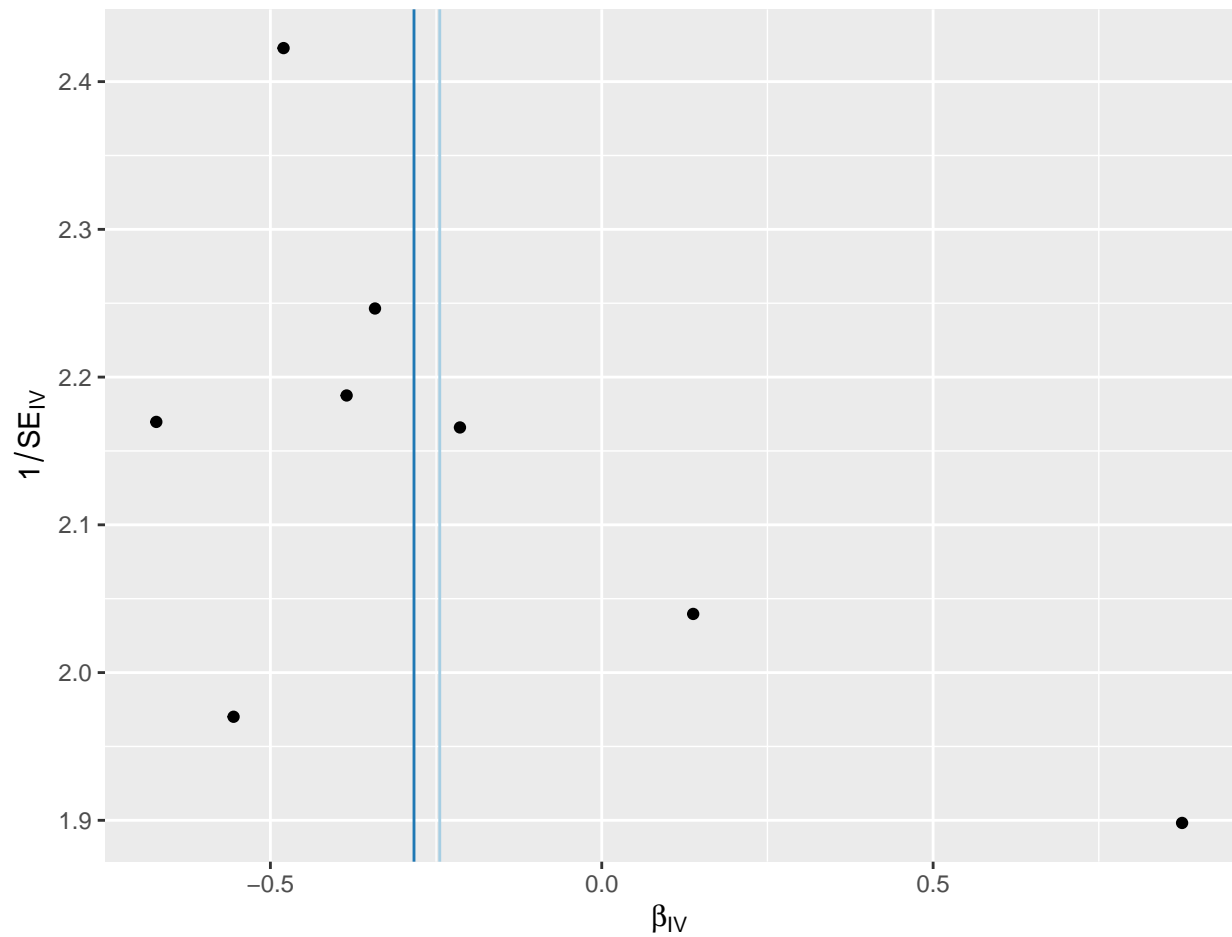

Supplement: Supplementary file 1 [file Data_Sheet_1.ZIP › data/GCST90274761_5e6/761.funnel_plot.pdf]

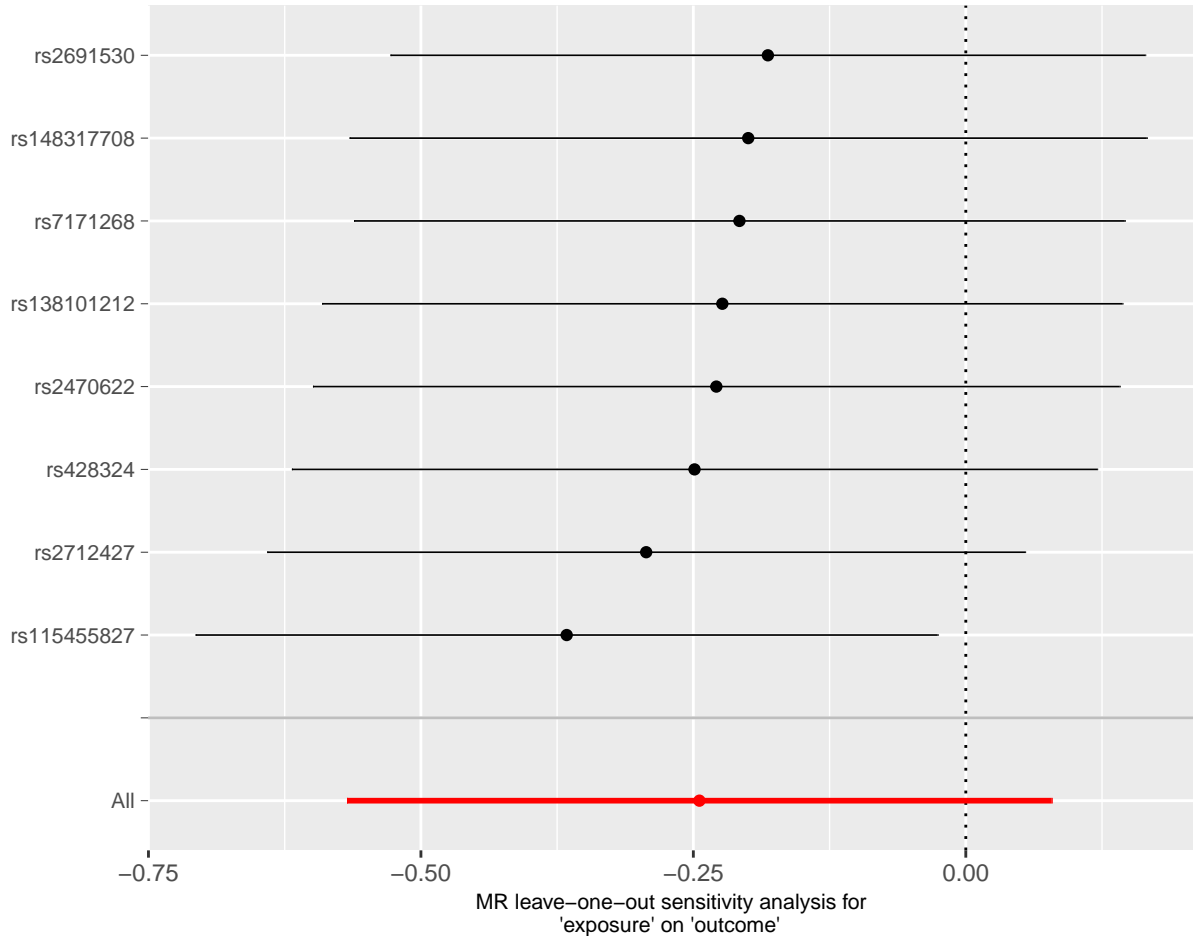

Supplement: Supplementary file 1 [file Data_Sheet_1.ZIP › data/GCST90274761_5e6/761.leaveoneout.pdf]

# MR Test

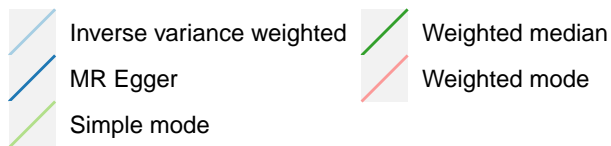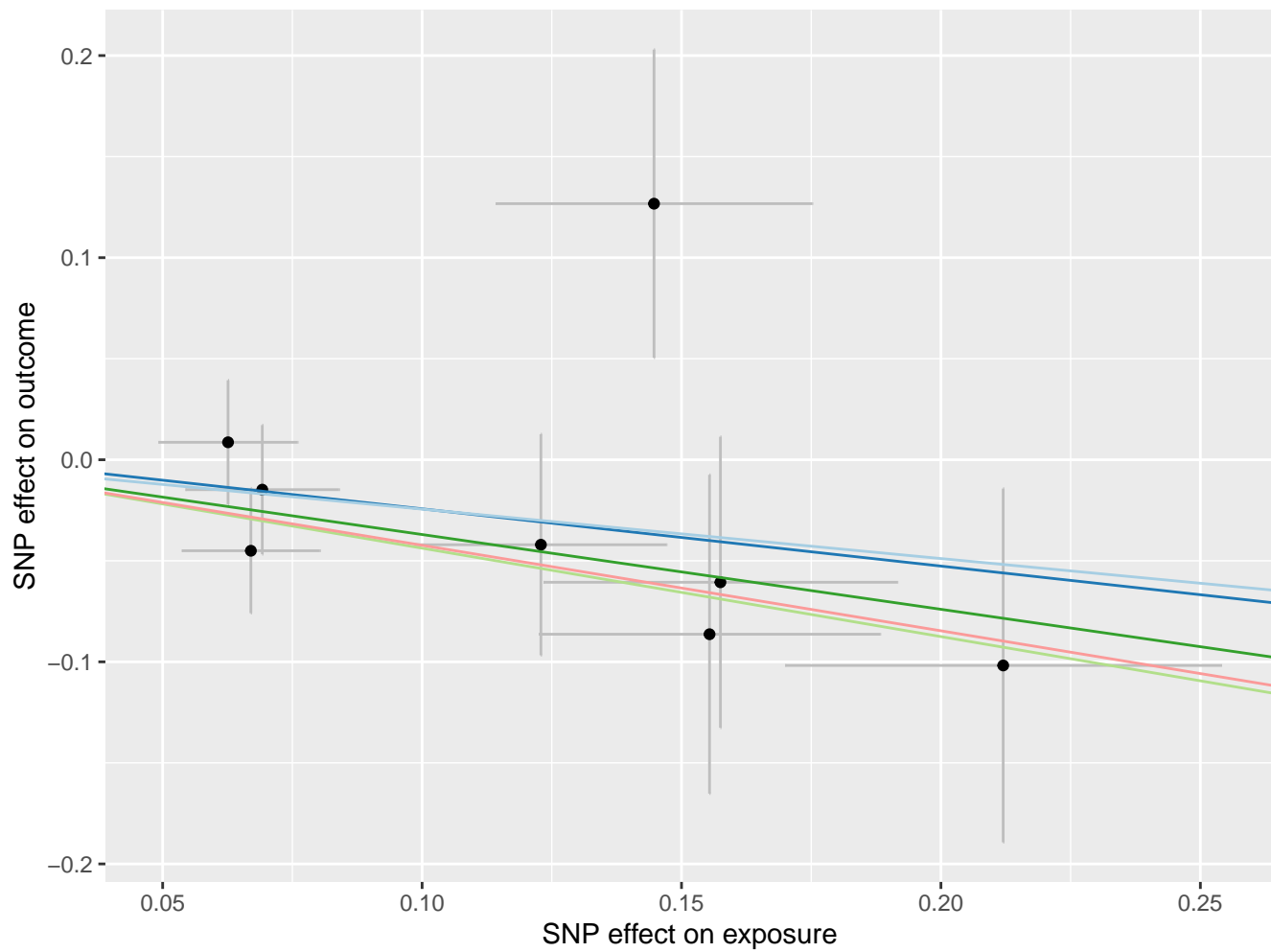

Supplement: Supplementary file 1 [file Data_Sheet_1.ZIP › data/GCST90274761_5e6/761.scatter_plot.pdf]

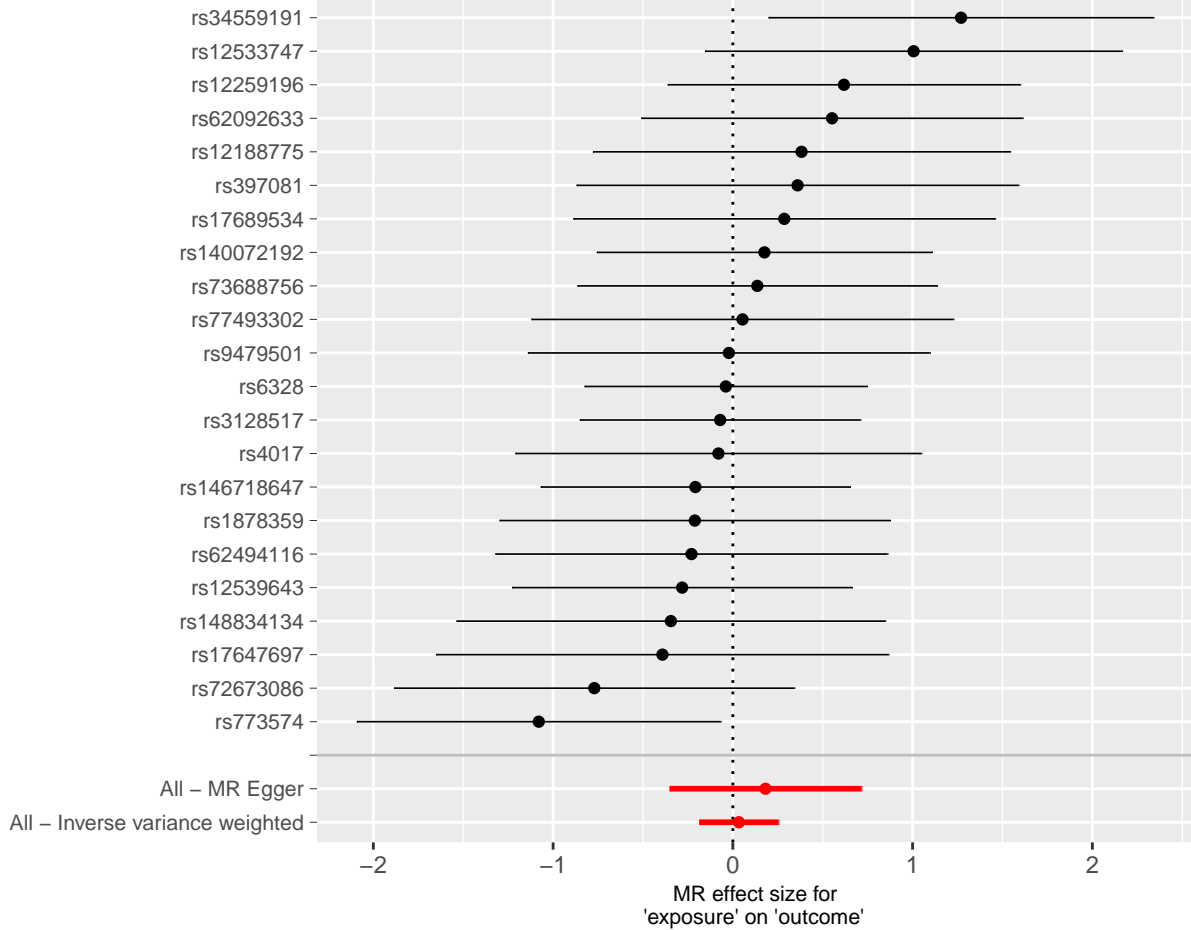

Supplement: Supplementary file 1 [file Data_Sheet_1.ZIP › data/GCST90274762_5e6/762.forest.pdf]

# MR Method

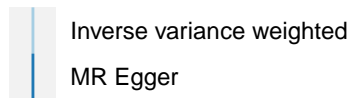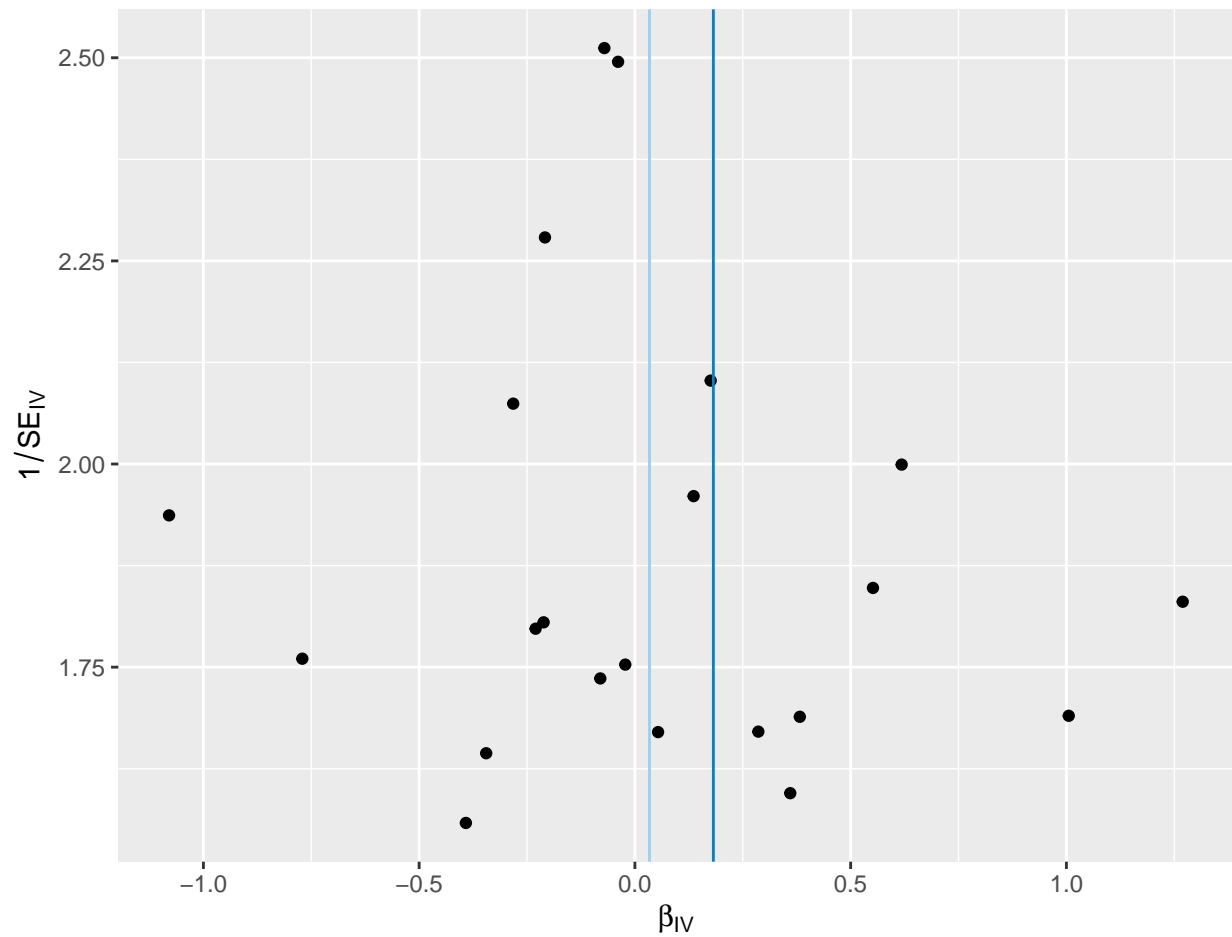

Supplement: Supplementary file 1 [file Data_Sheet_1.ZIP › data/GCST90274762_5e6/762.funnel_plot.pdf]

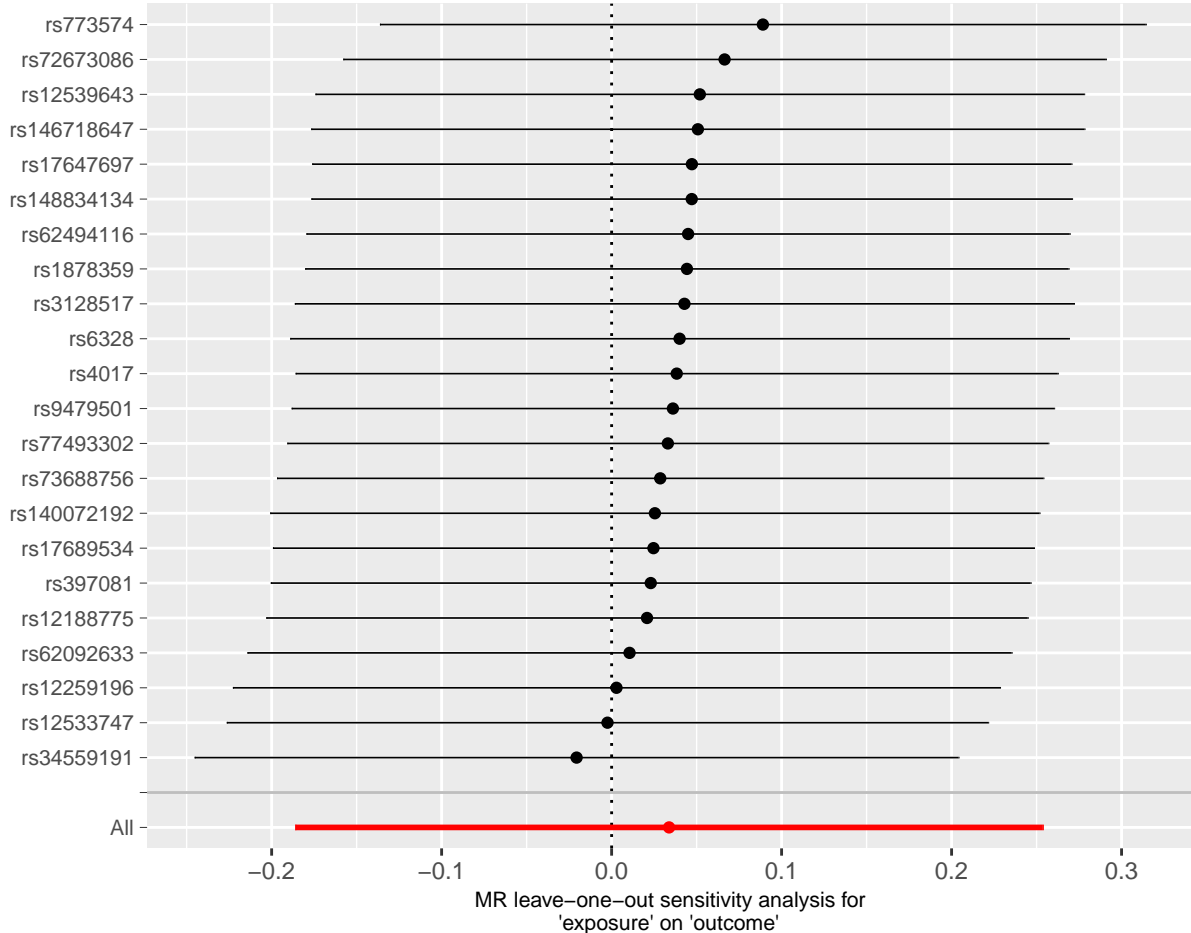

Supplement: Supplementary file 1 [file Data_Sheet_1.ZIP › data/GCST90274762_5e6/762.leaveoneout.pdf]

# MR Test

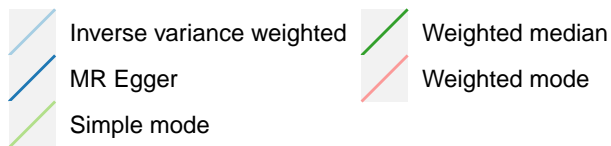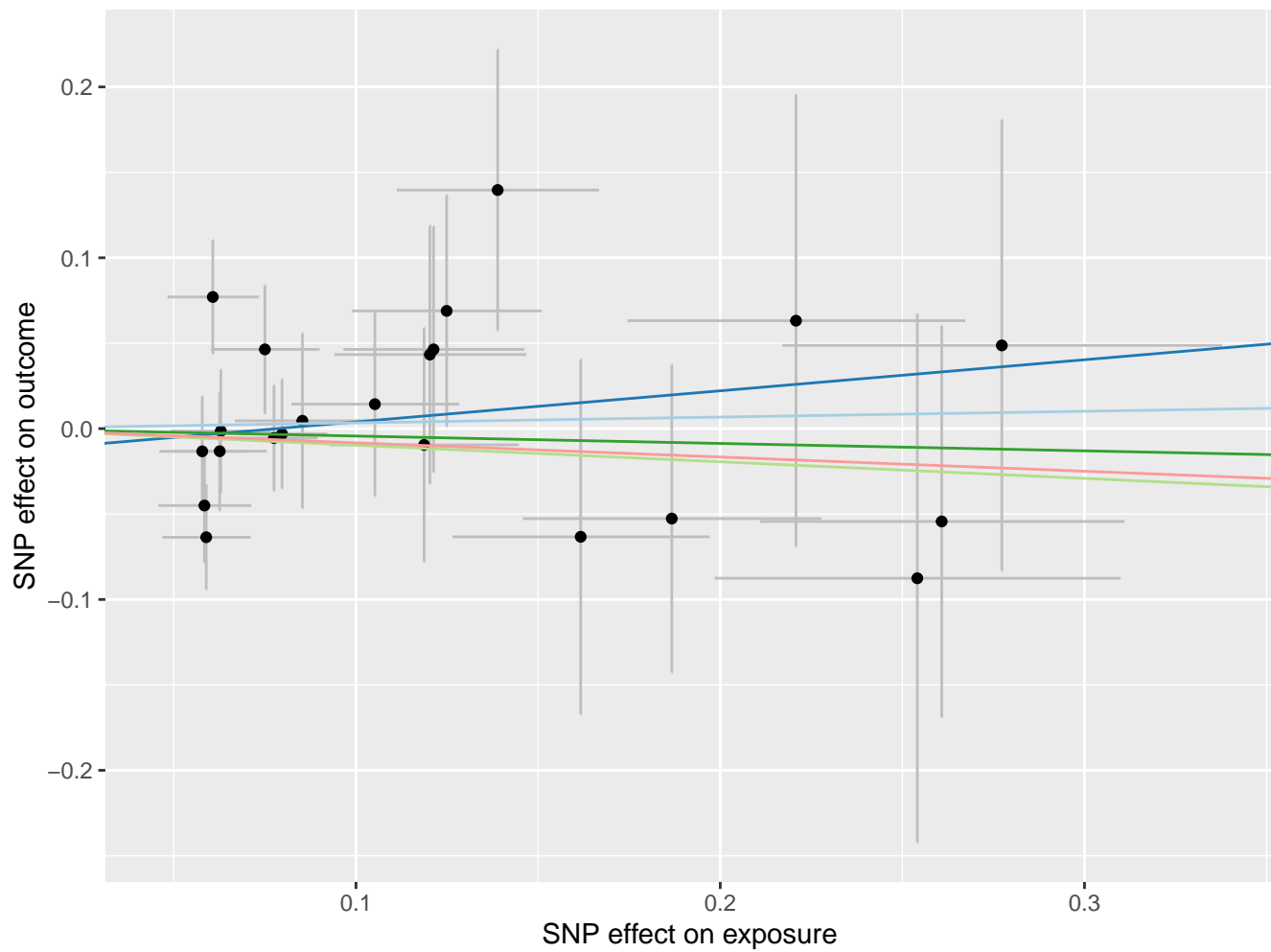

Supplement: Supplementary file 1 [file Data_Sheet_1.ZIP › data/GCST90274762_5e6/762.scatter_plot.pdf]

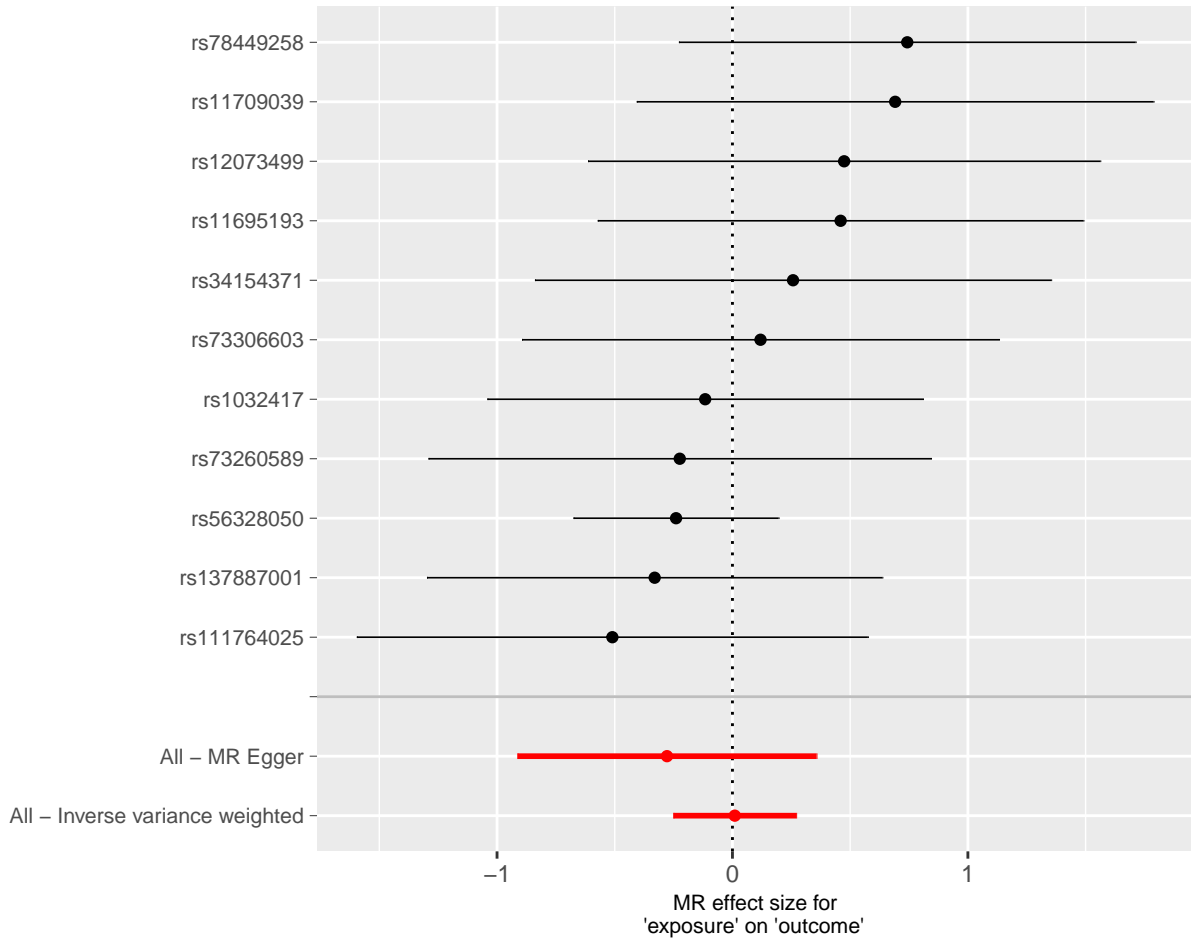

Supplement: Supplementary file 1 [file Data_Sheet_1.ZIP › data/GCST90274763_5e6/763.forest.pdf]

# MR Method

- Inverse variance weighted
- MR Egger

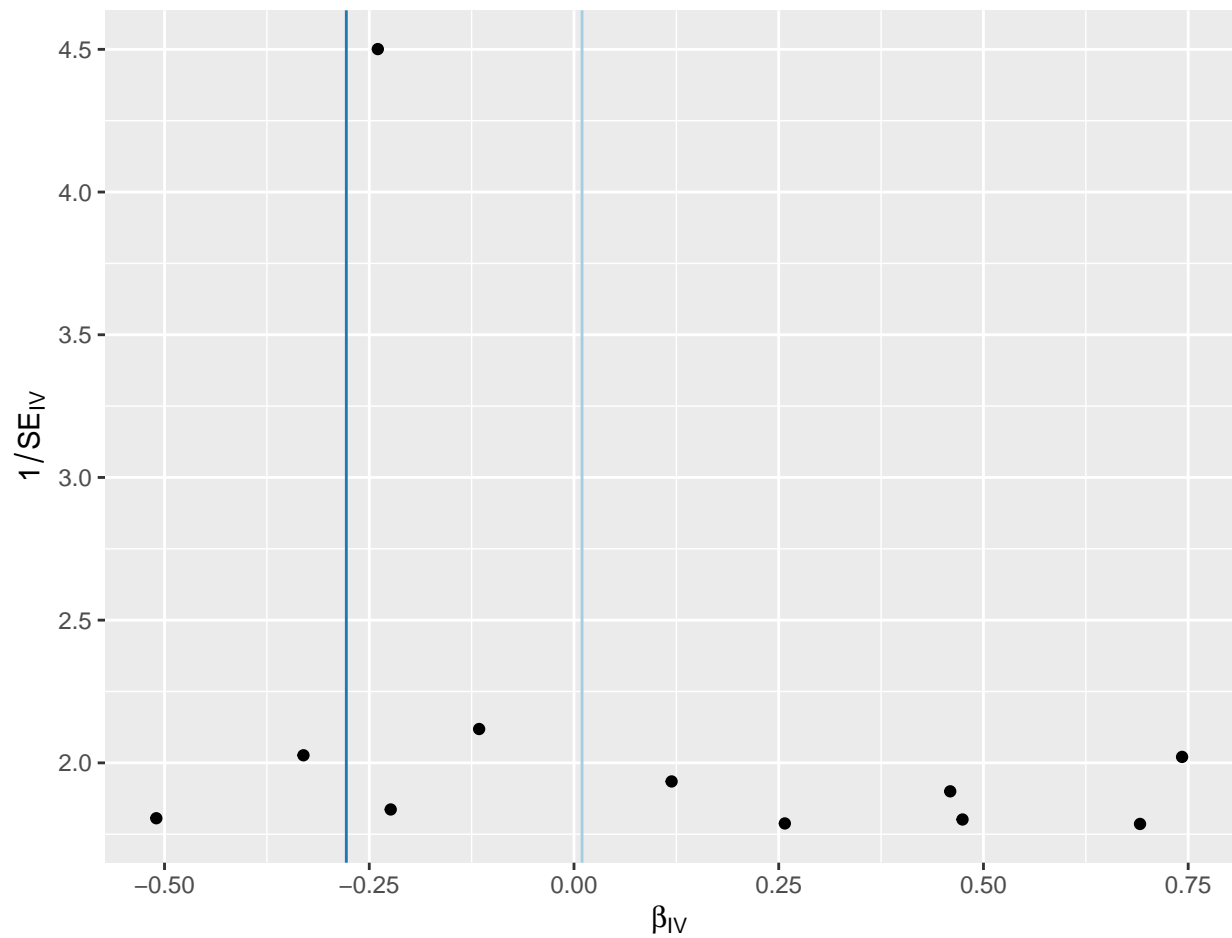

Supplement: Supplementary file 1 [file Data_Sheet_1.ZIP › data/GCST90274763_5e6/763.funnel_plot.pdf]

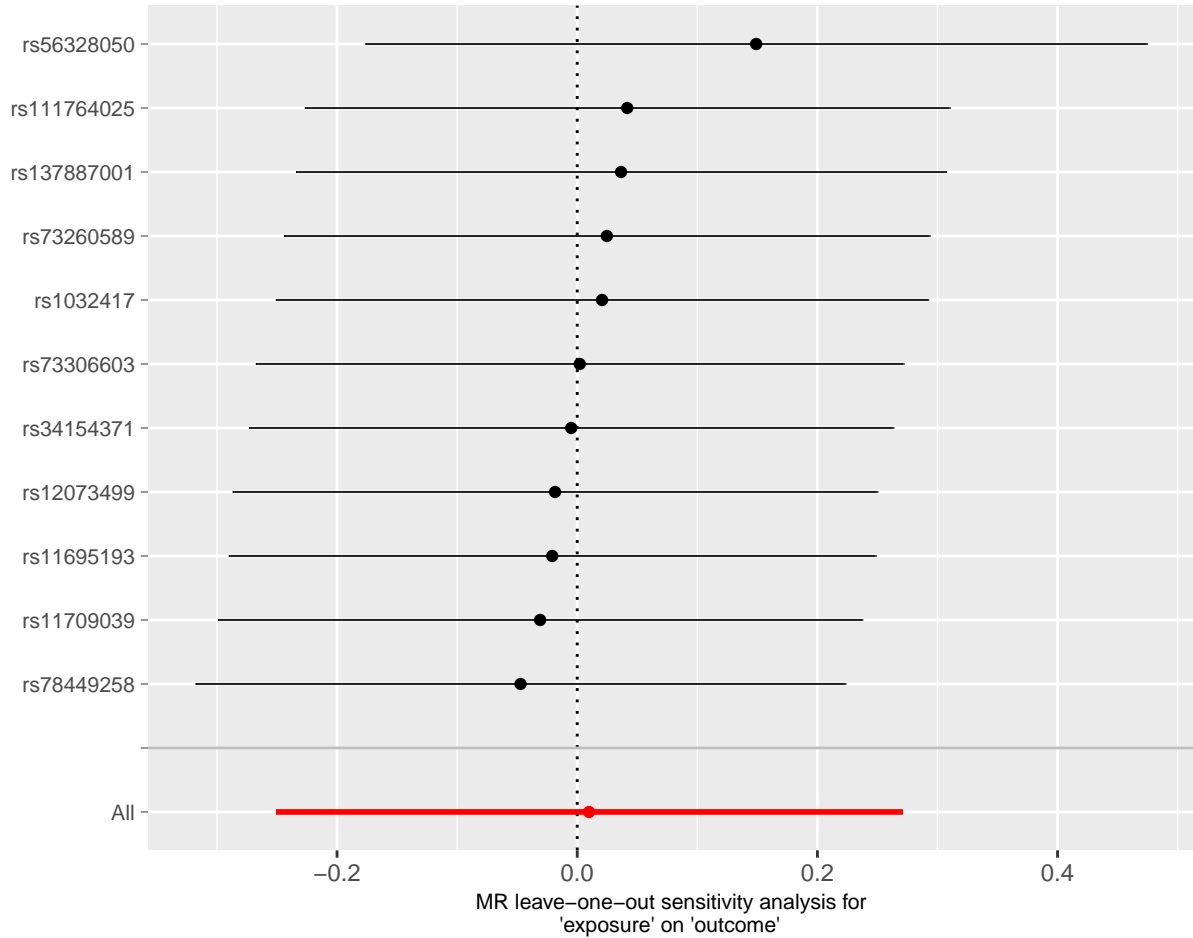

Supplement: Supplementary file 1 [file Data_Sheet_1.ZIP › data/GCST90274763_5e6/763.leaveoneout.pdf]

# MR Test

- Inverse variance weighted
- MR Egger
- Simple mode
- Weighted median
- Weighted mode

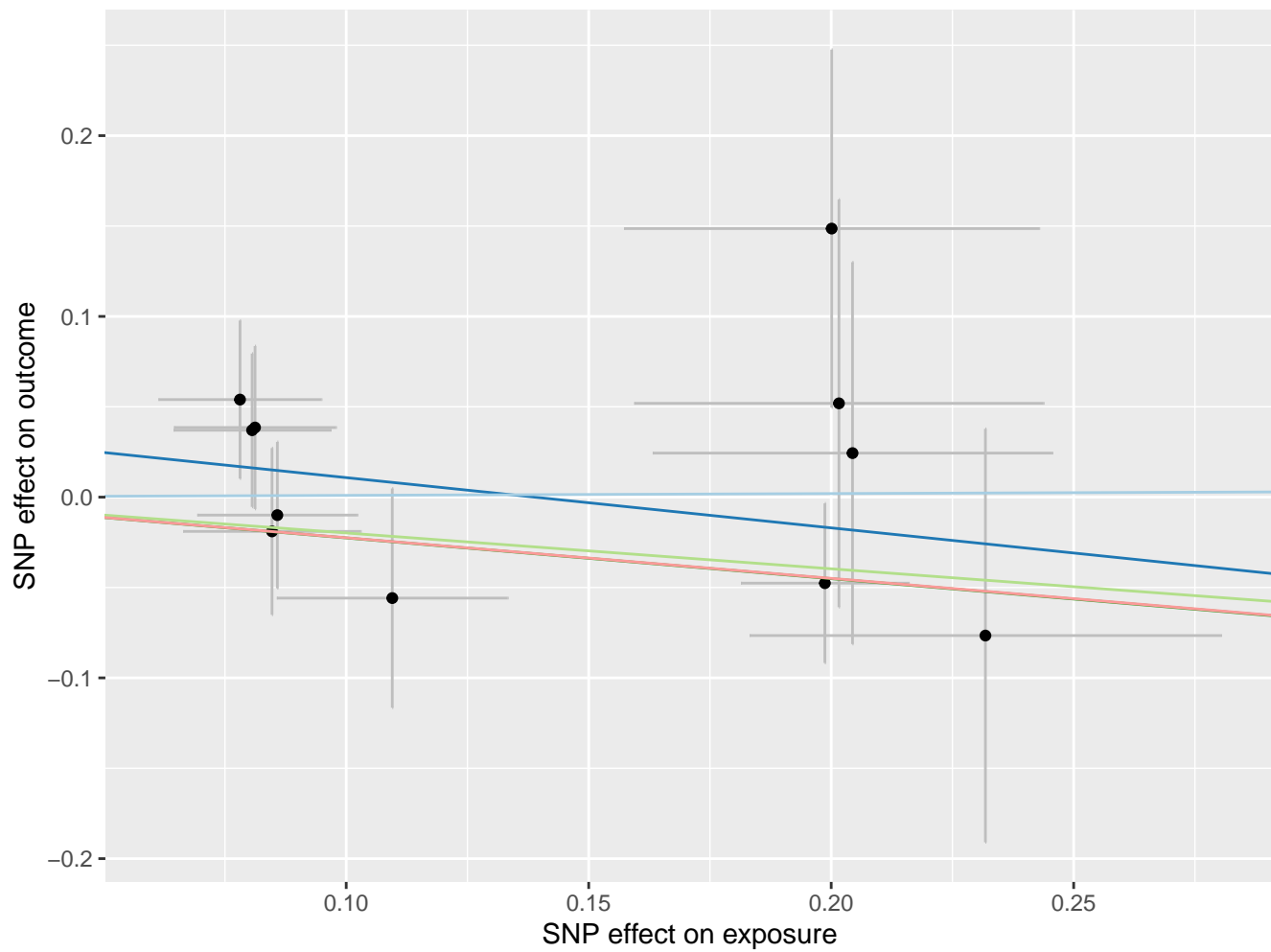

Supplement: Supplementary file 1 [file Data_Sheet_1.ZIP › data/GCST90274763_5e6/763.scatter_plot.pdf]

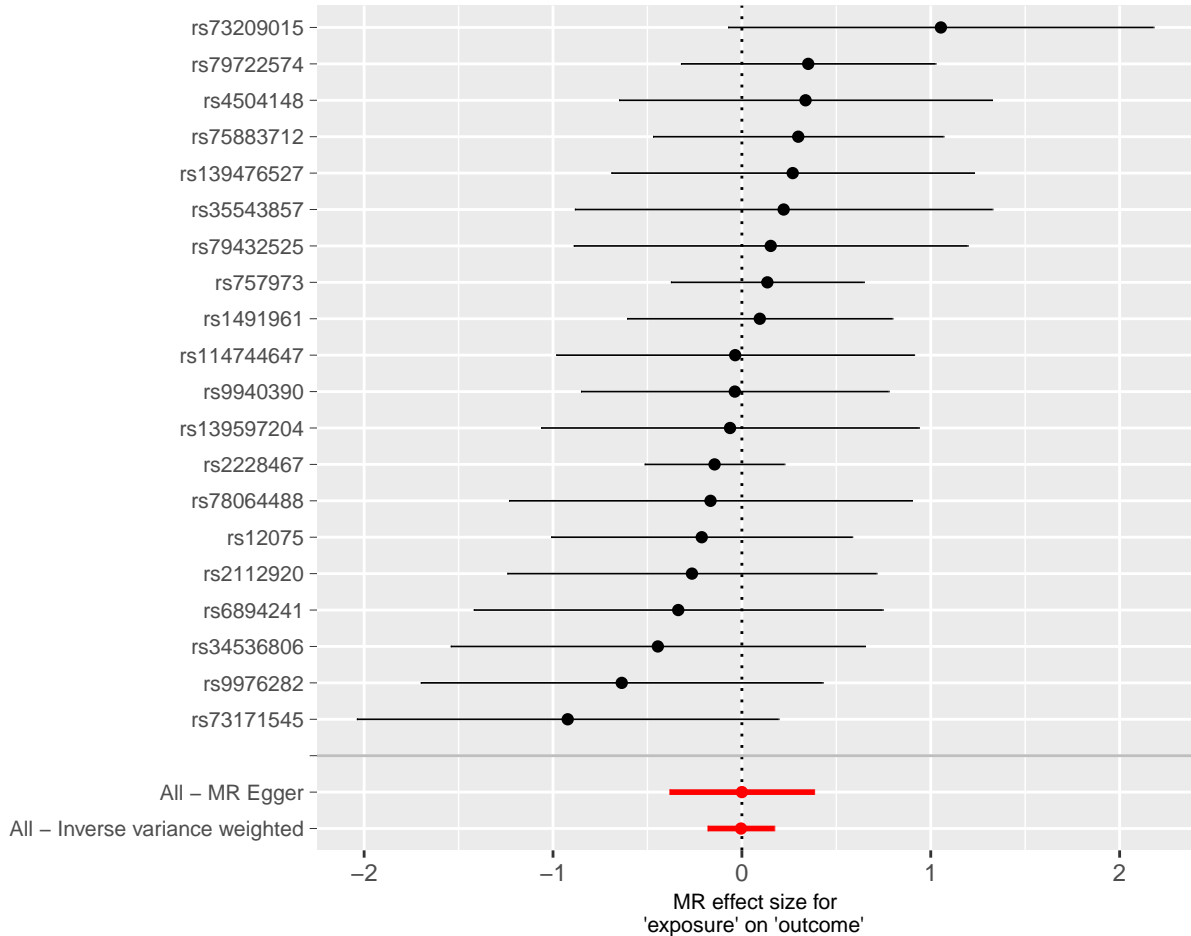

Supplement: Supplementary file 1 [file Data_Sheet_1.ZIP › data/GCST90274764_5e6/764.forest.pdf]

# MR Method

- Inverse variance weighted
- MR Egger

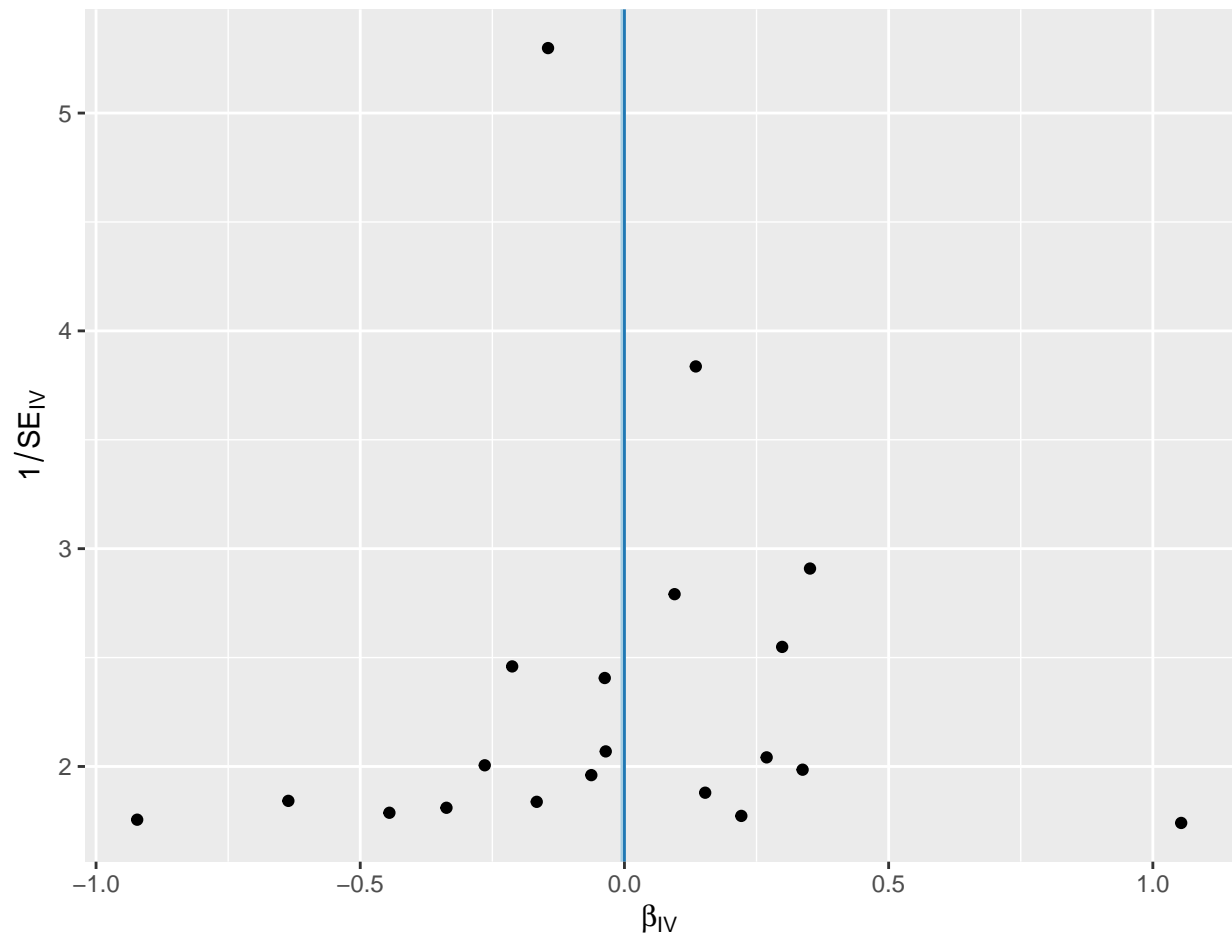

Supplement: Supplementary file 1 [file Data_Sheet_1.ZIP › data/GCST90274764_5e6/764.funnel_plot.pdf]

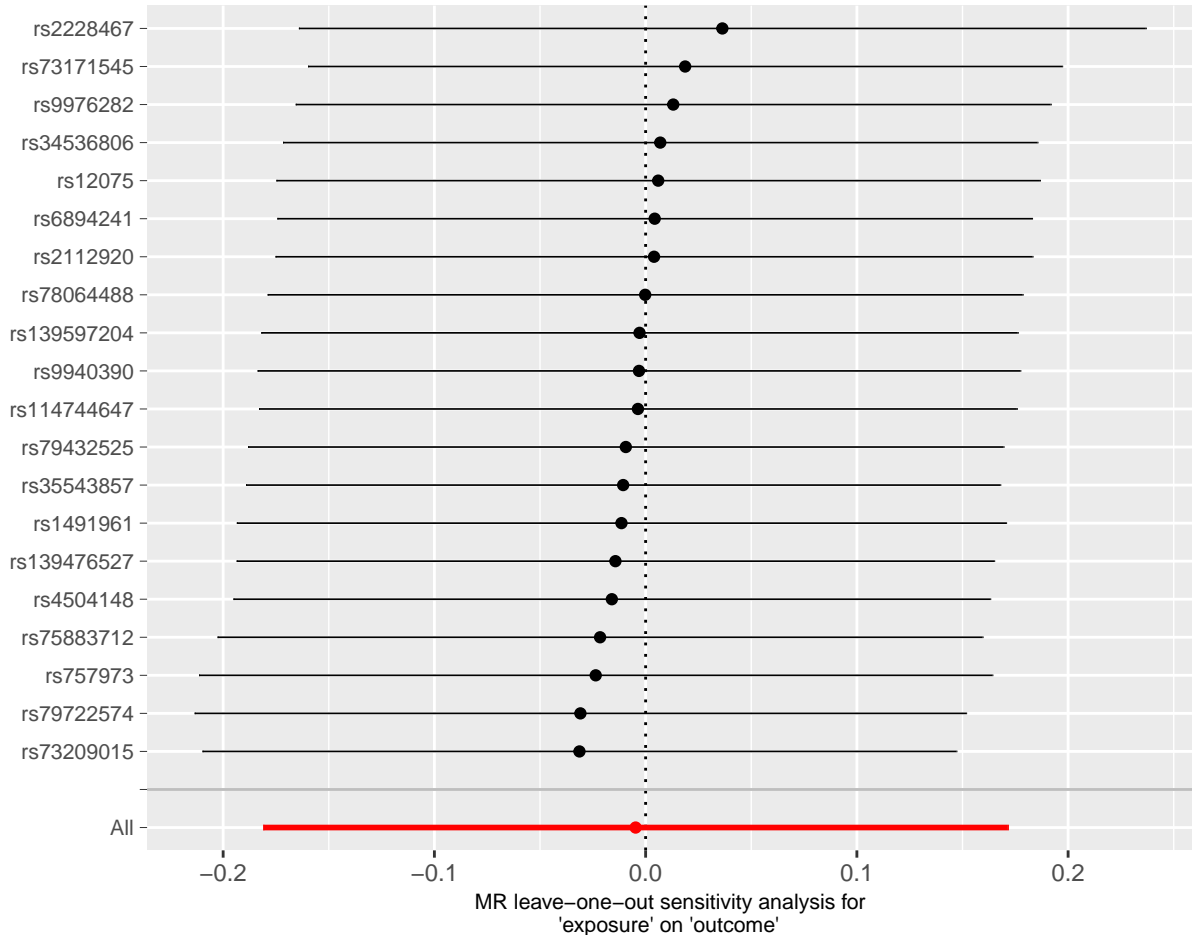

Supplement: Supplementary file 1 [file Data_Sheet_1.ZIP › data/GCST90274764_5e6/764.leaveoneout.pdf]

# MR Test

- Inverse variance weighted
- MR Egger
- Simple mode
- Weighted median
- Weighted mode

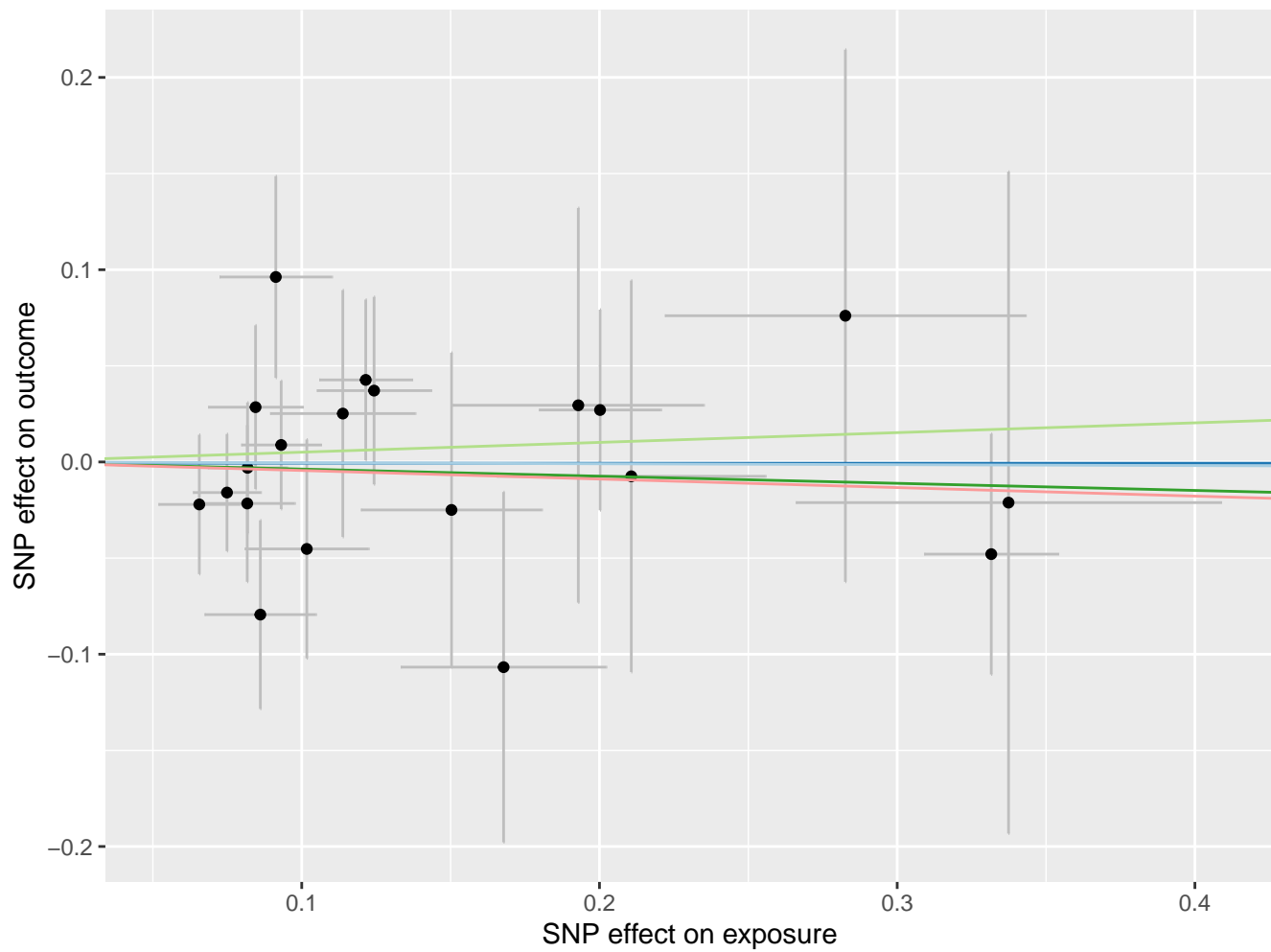

Supplement: Supplementary file 1 [file Data_Sheet_1.ZIP › data/GCST90274764_5e6/764.scatter_plot.pdf]

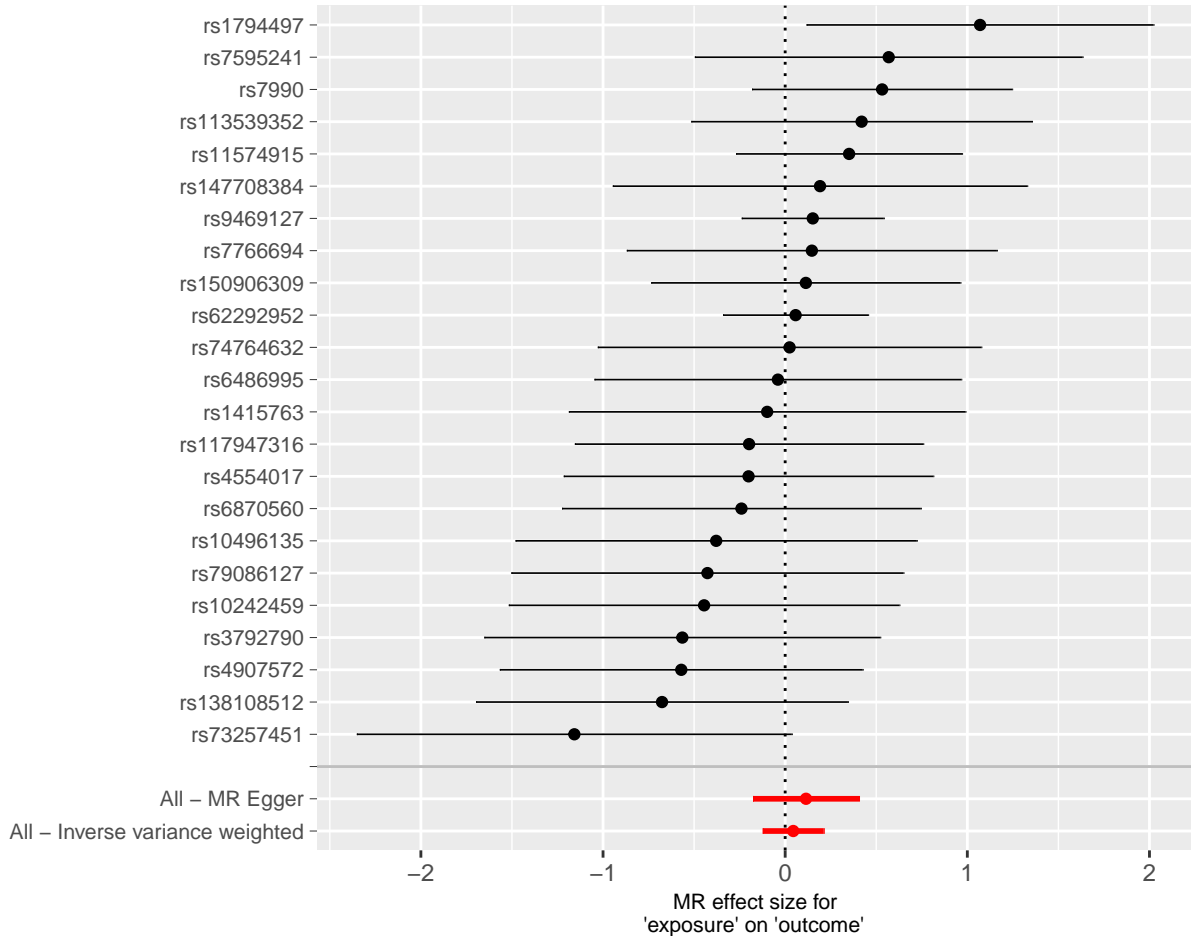

Supplement: Supplementary file 1 [file Data_Sheet_1.ZIP › data/GCST90274765_5e6/765.forest.pdf]

# MR Method

- Inverse variance weighted
- MR Egger

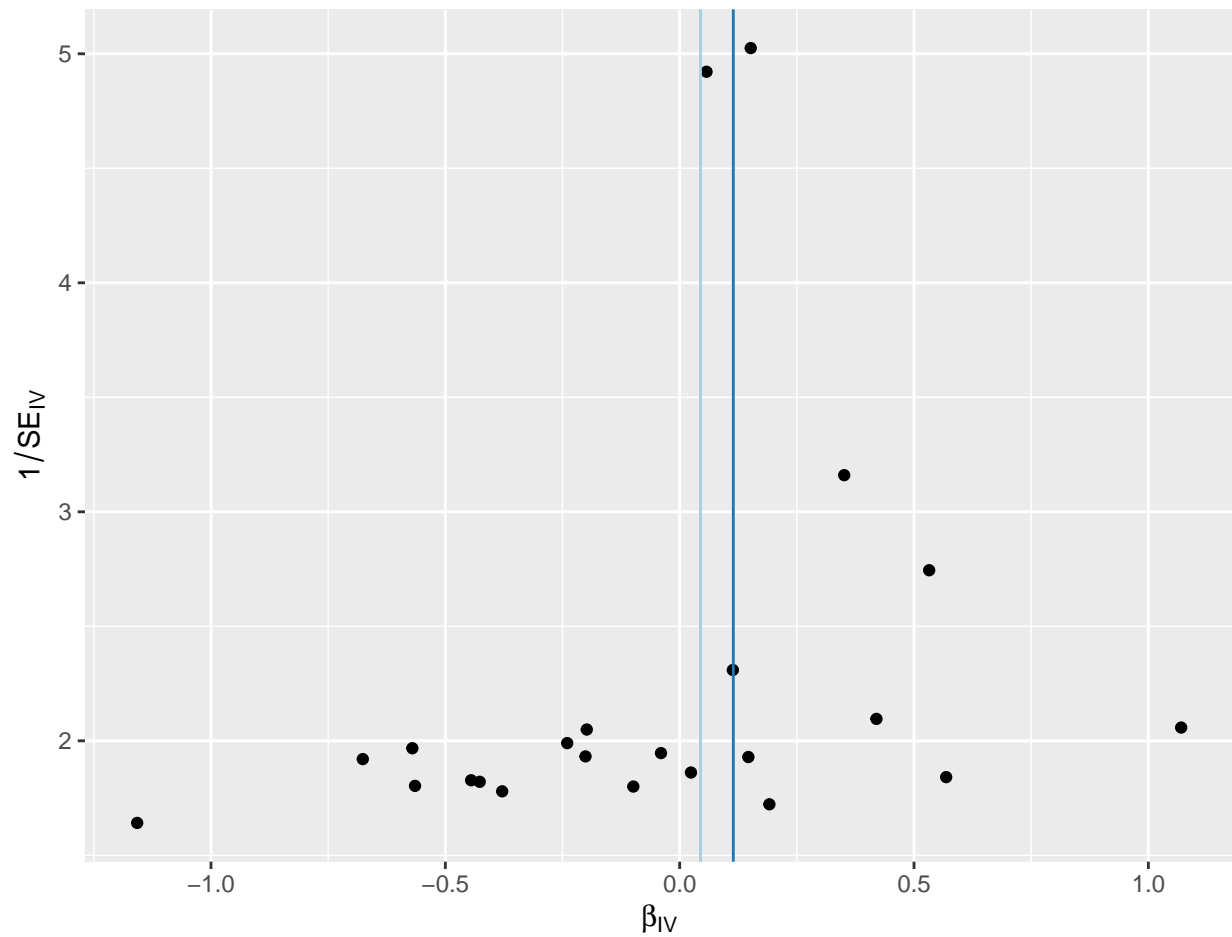

Supplement: Supplementary file 1 [file Data_Sheet_1.ZIP › data/GCST90274765_5e6/765.funnel_plot.pdf]

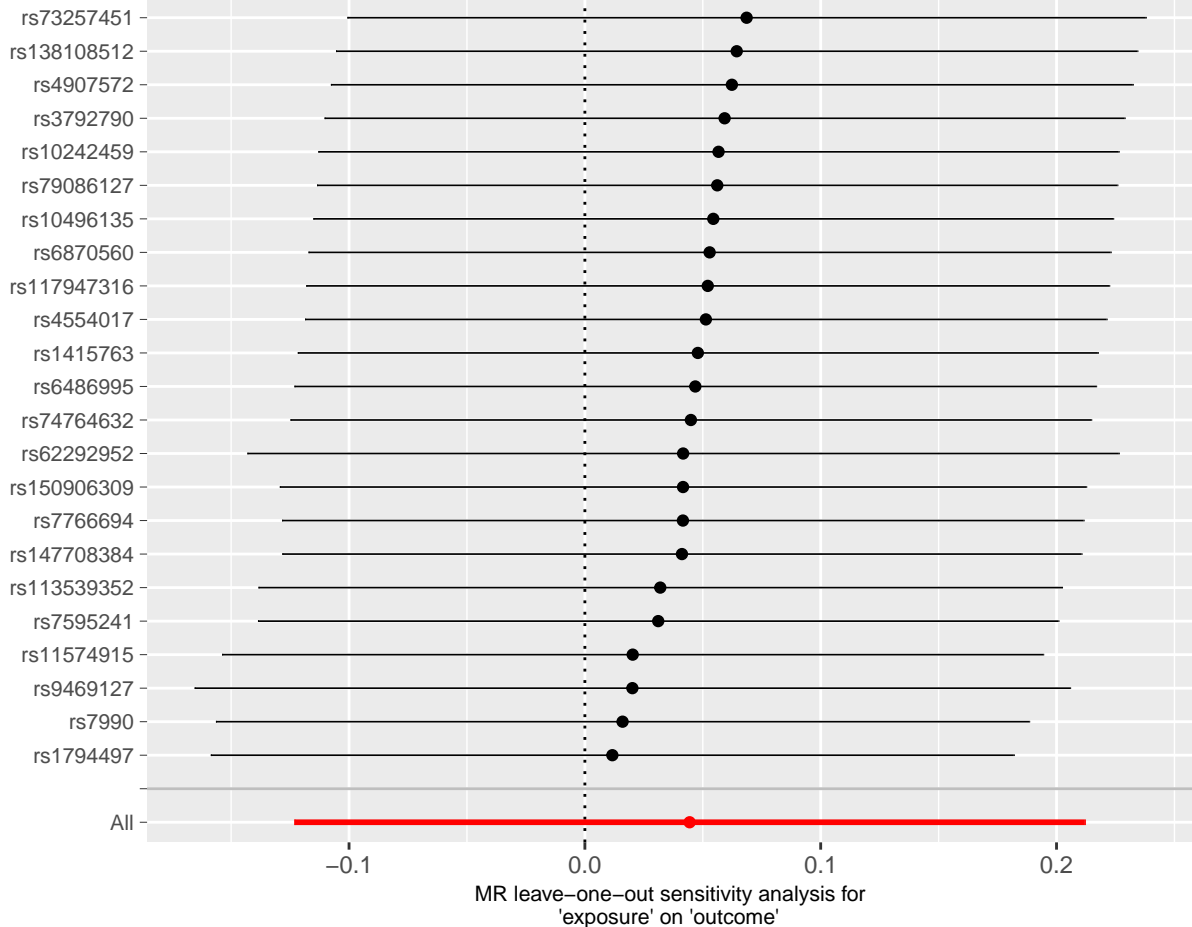

Supplement: Supplementary file 1 [file Data_Sheet_1.ZIP › data/GCST90274765_5e6/765.leaveoneout.pdf]

# MR Test

- Inverse variance weighted
- MR Egger
- Simple mode
- Weighted median
- Weighted mode

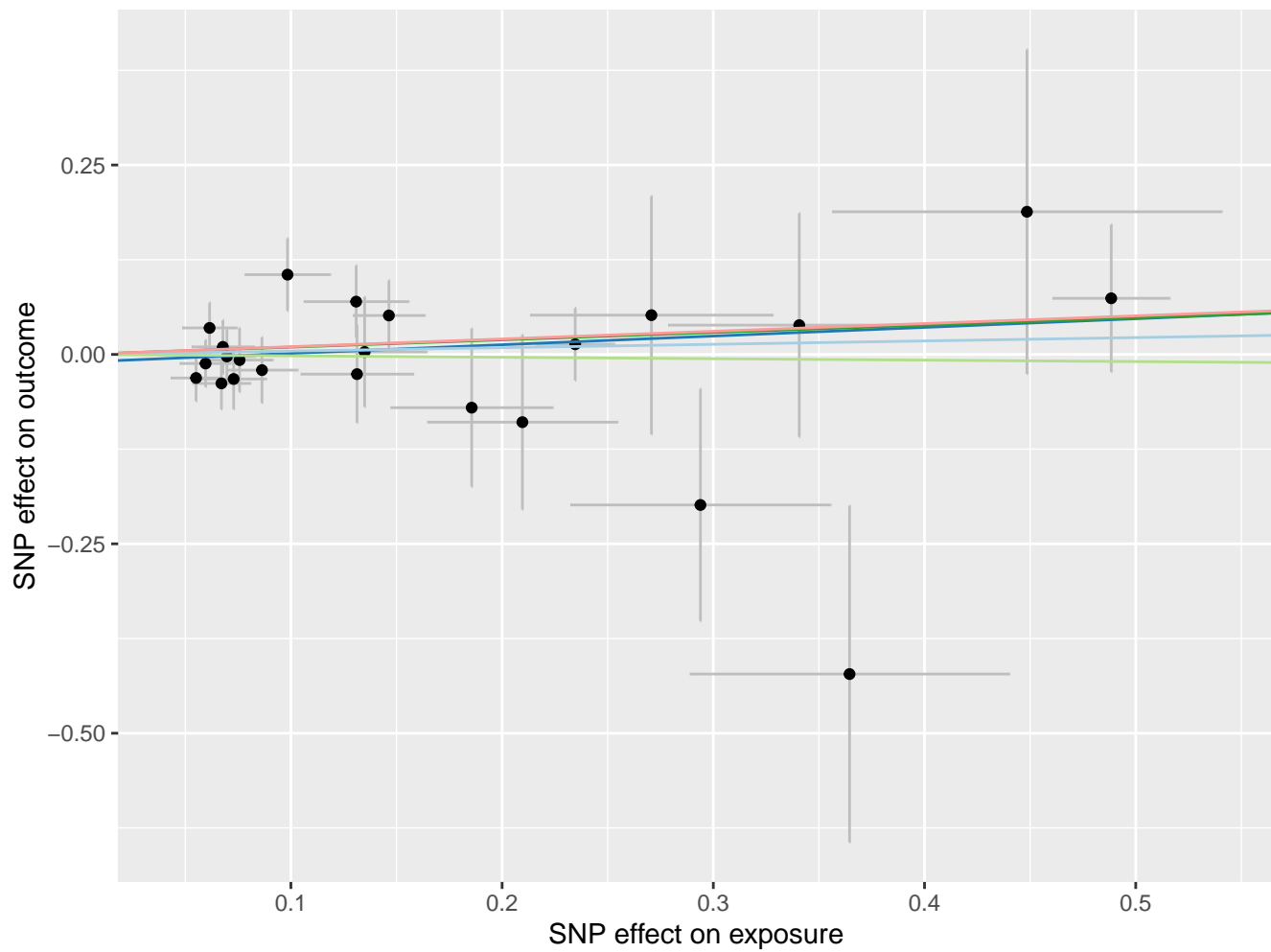

Supplement: Supplementary file 1 [file Data_Sheet_1.ZIP › data/GCST90274765_5e6/765.scatter_plot.pdf]

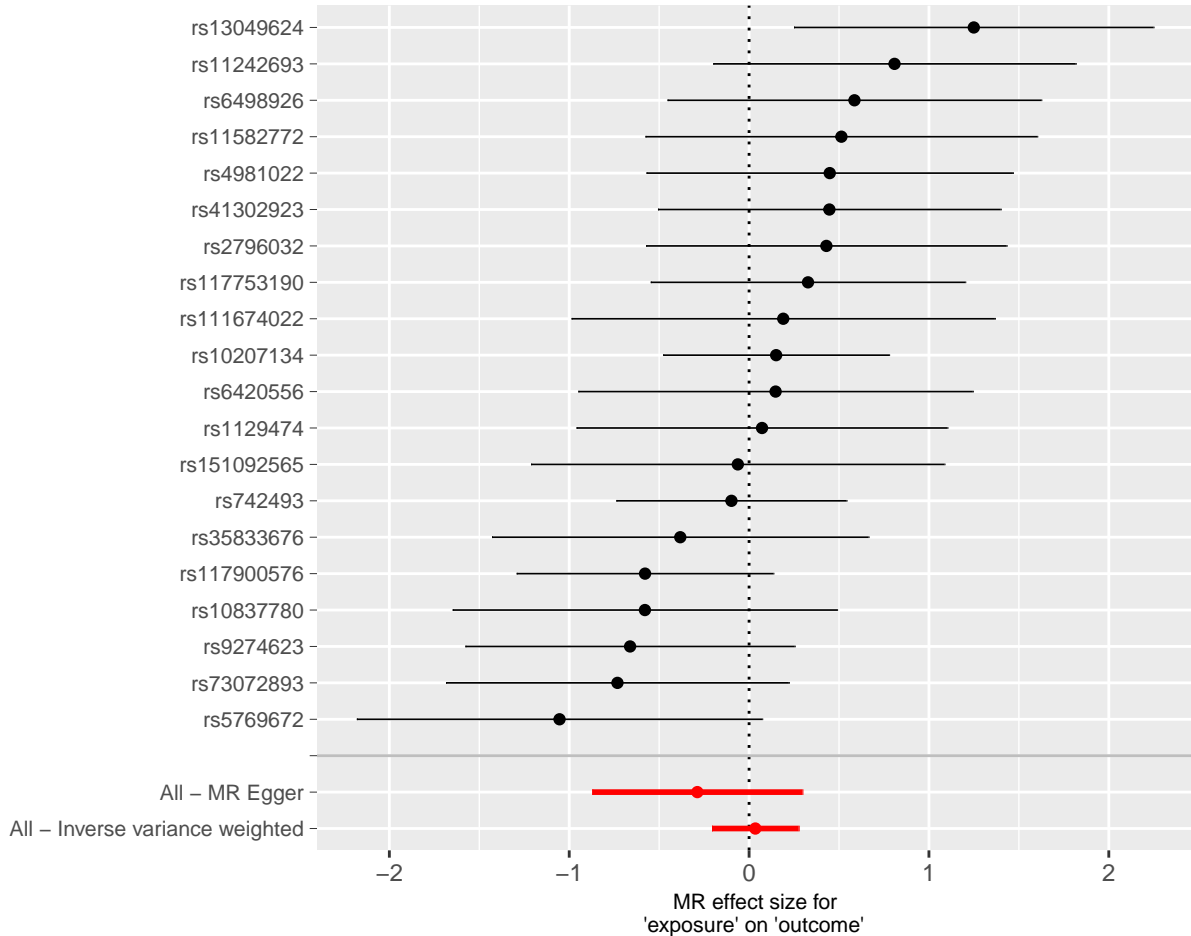

Supplement: Supplementary file 1 [file Data_Sheet_1.ZIP › data/GCST90274766_5e6/766.forest.pdf]

# MR Method

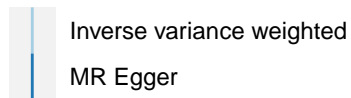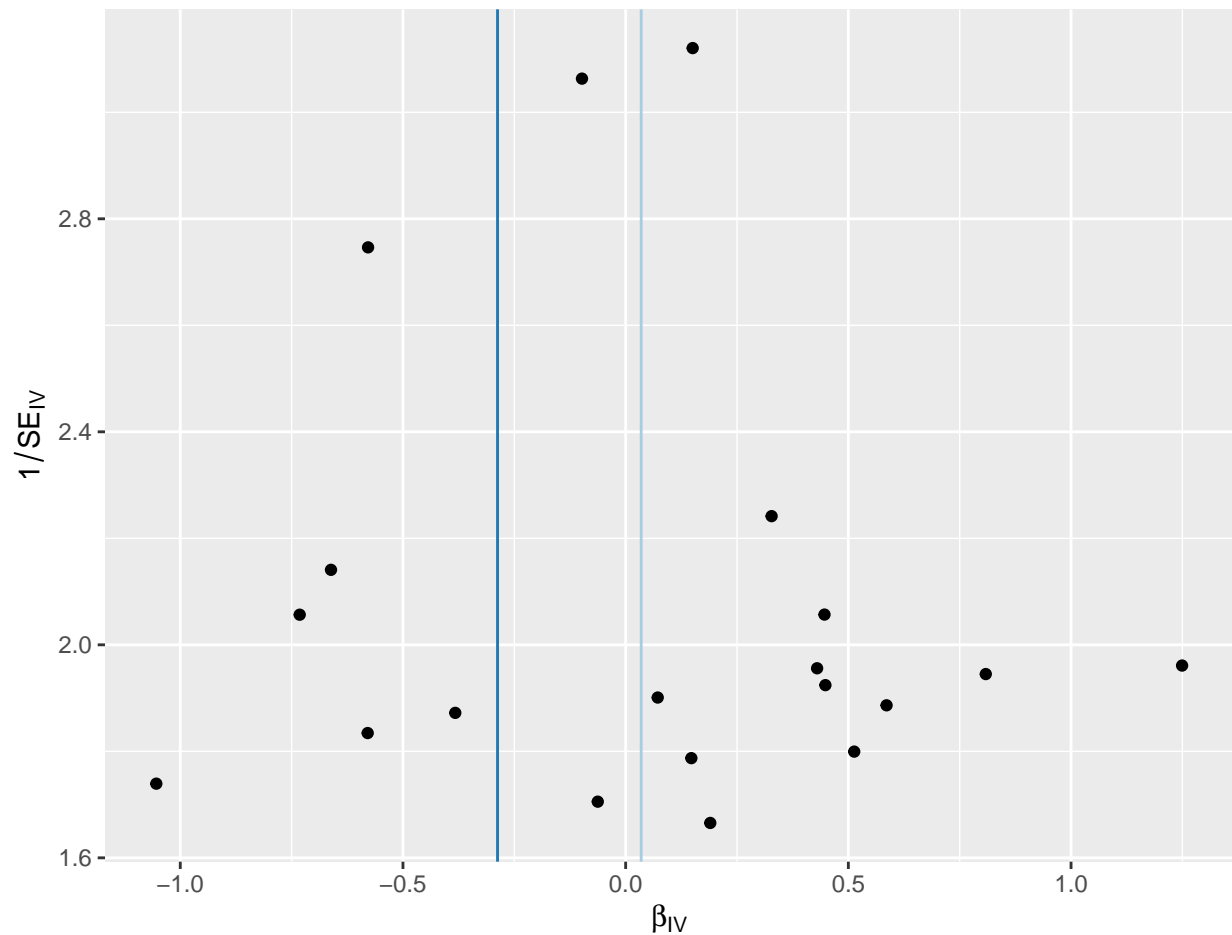

Supplement: Supplementary file 1 [file Data_Sheet_1.ZIP › data/GCST90274766_5e6/766.funnel_plot.pdf]

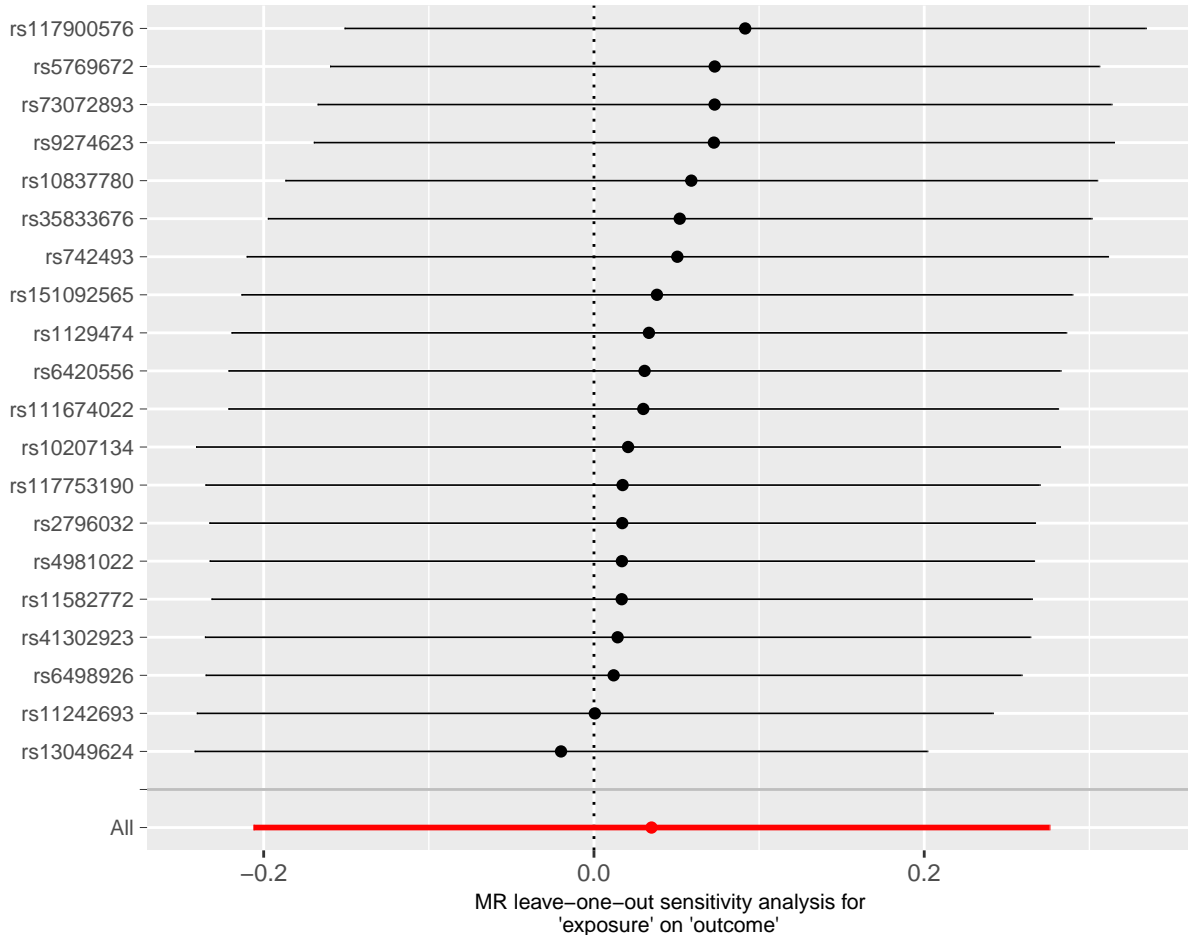

Supplement: Supplementary file 1 [file Data_Sheet_1.ZIP › data/GCST90274766_5e6/766.leaveoneout.pdf]

# MR Test

- Inverse variance weighted
- MR Egger
- Simple mode
- Weighted median
- Weighted mode

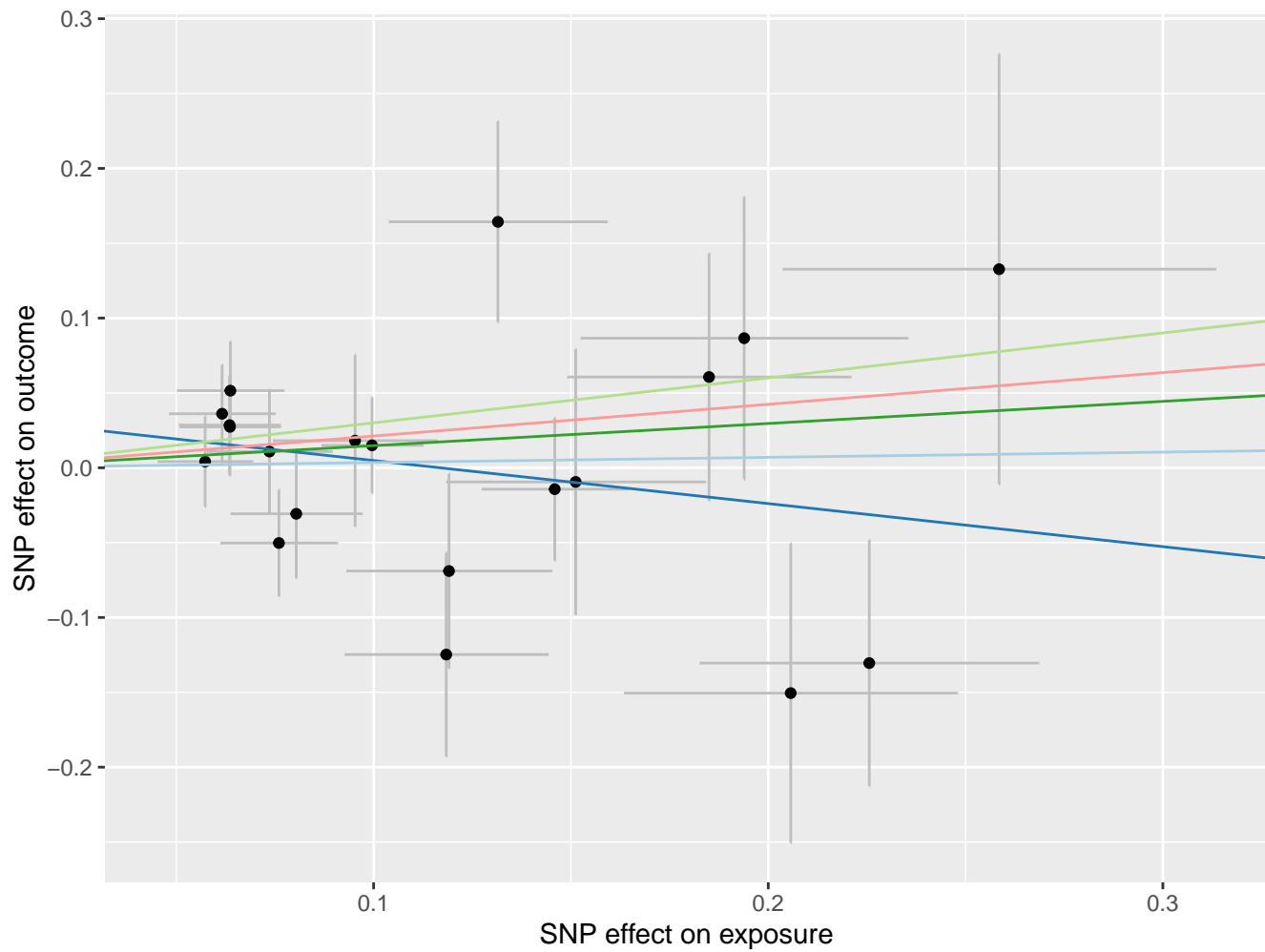

Supplement: Supplementary file 1 [file Data_Sheet_1.ZIP › data/GCST90274766_5e6/766.scatter_plot.pdf]

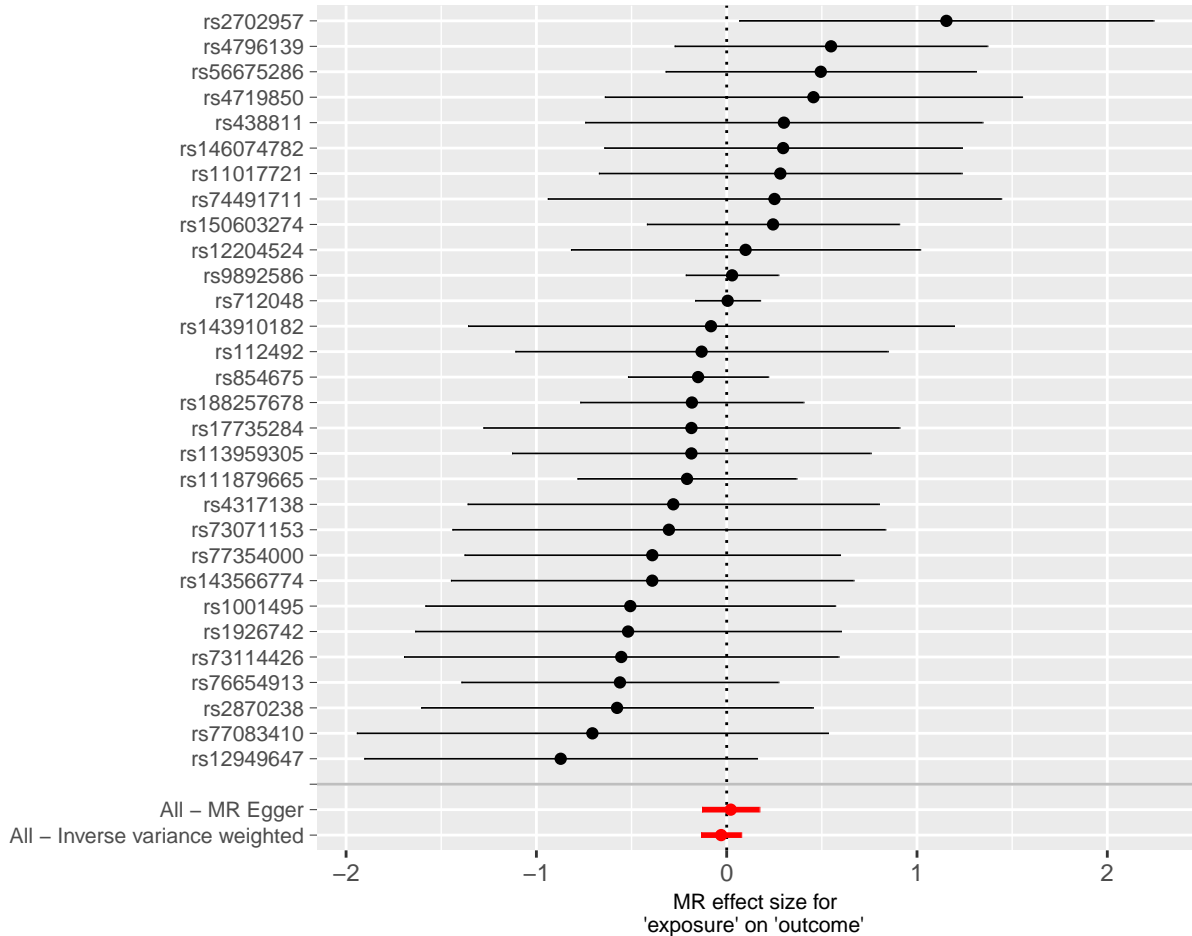

Supplement: Supplementary file 1 [file Data_Sheet_1.ZIP › data/GCST90274767_5e6/767.forest.pdf]

# MR Method

- Inverse variance weighted
- MR Egger

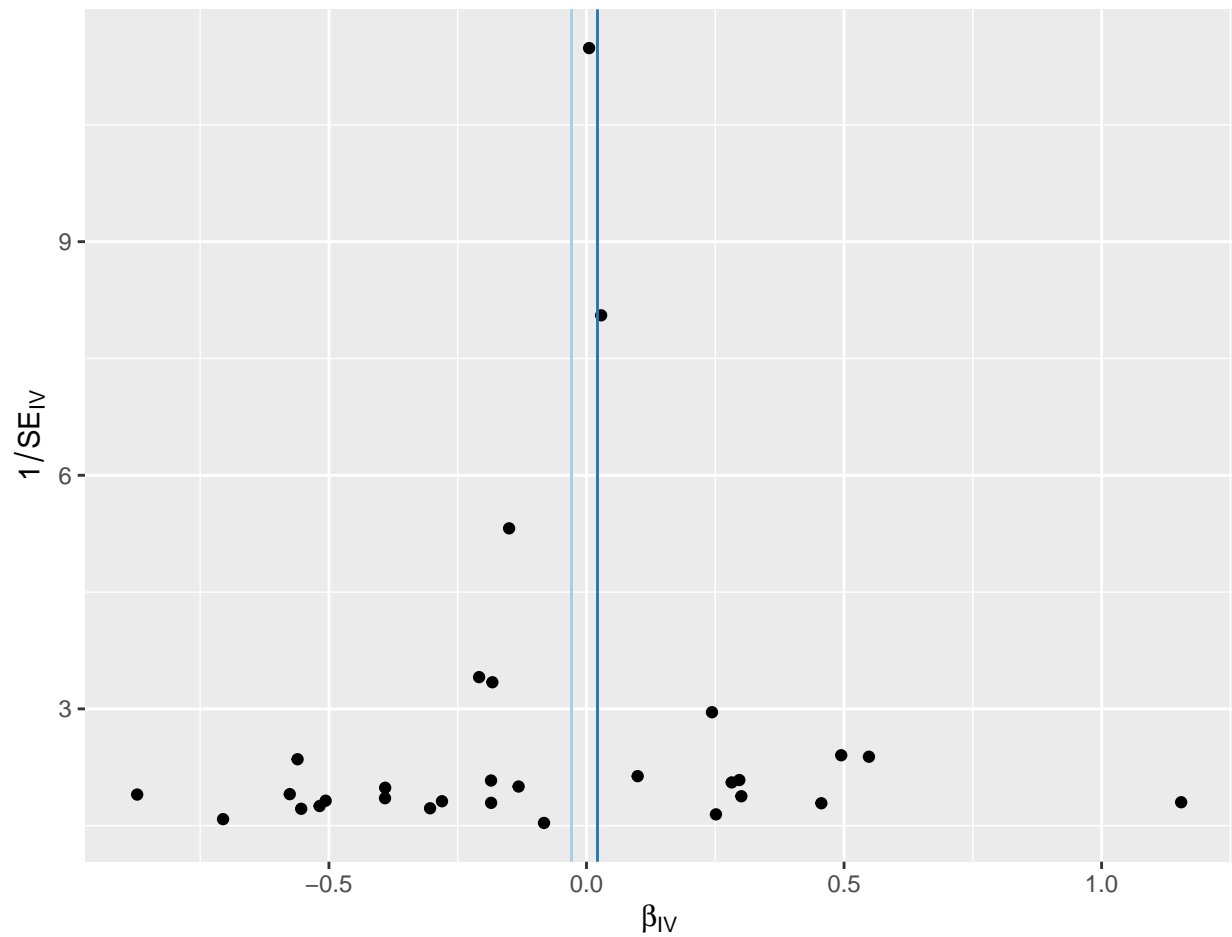

Supplement: Supplementary file 1 [file Data_Sheet_1.ZIP › data/GCST90274767_5e6/767.funnel_plot.pdf]

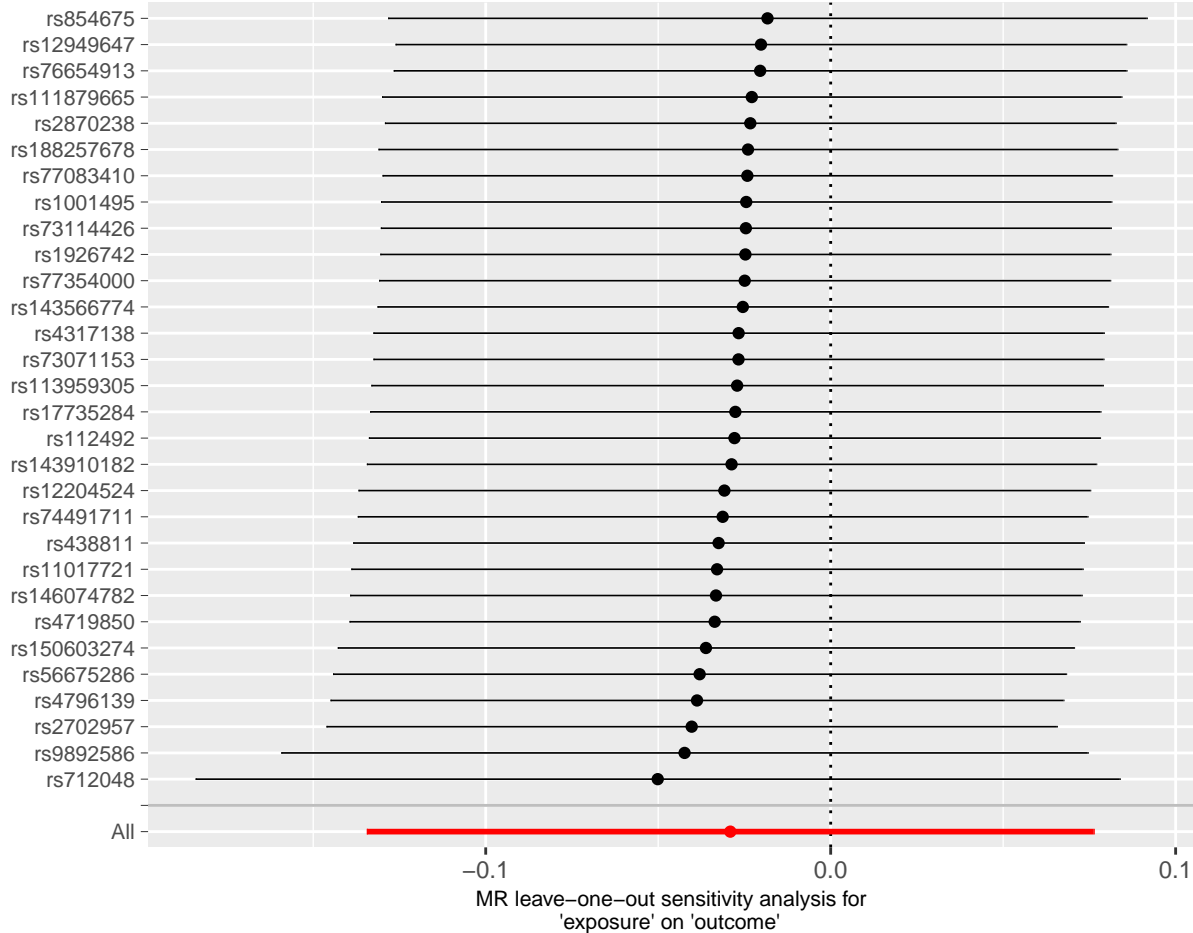

Supplement: Supplementary file 1 [file Data_Sheet_1.ZIP › data/GCST90274767_5e6/767.leaveoneout.pdf]

# MR Test

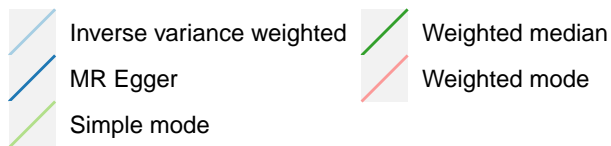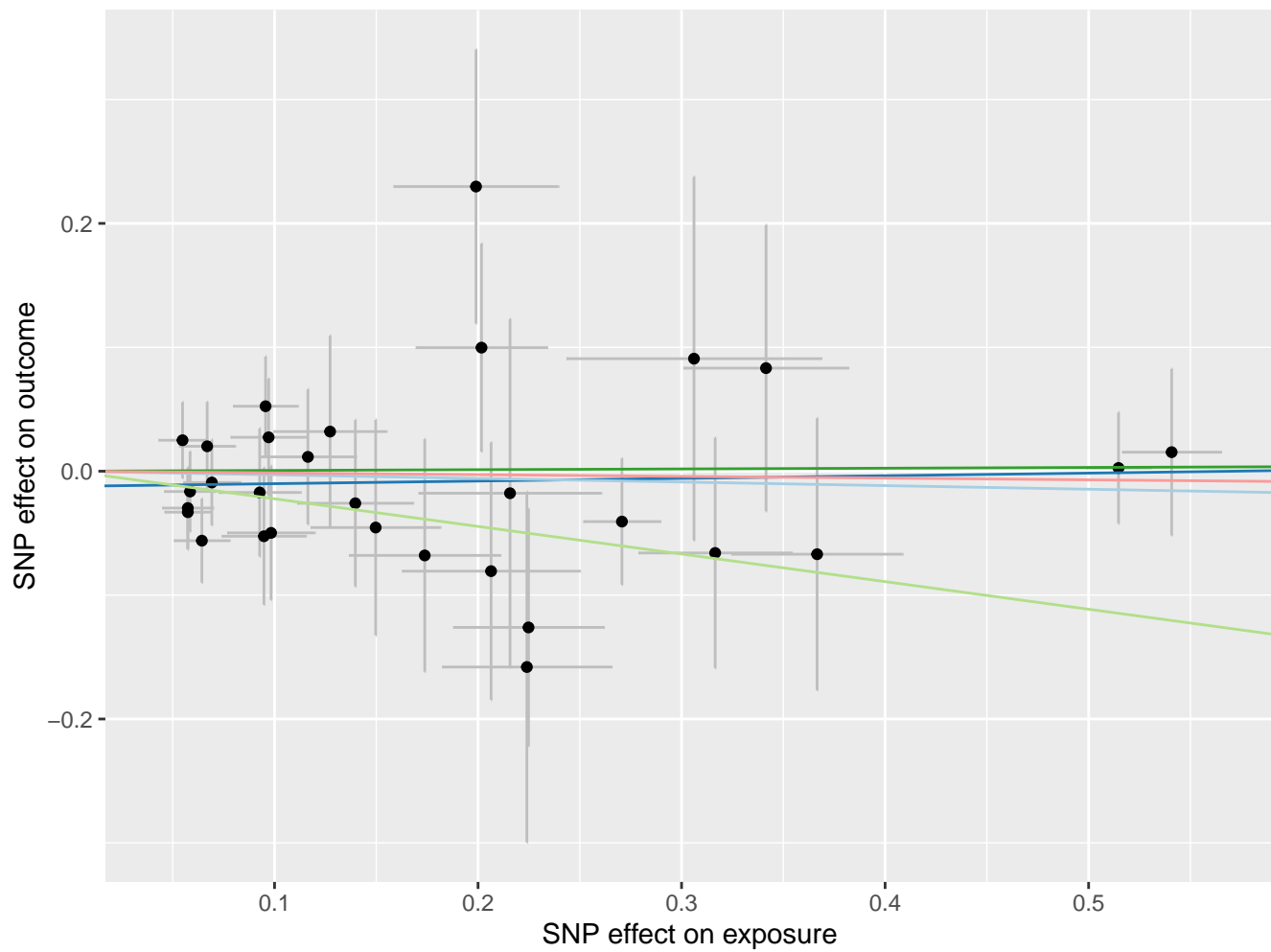

Supplement: Supplementary file 1 [file Data_Sheet_1.ZIP › data/GCST90274767_5e6/767.scatter_plot.pdf]

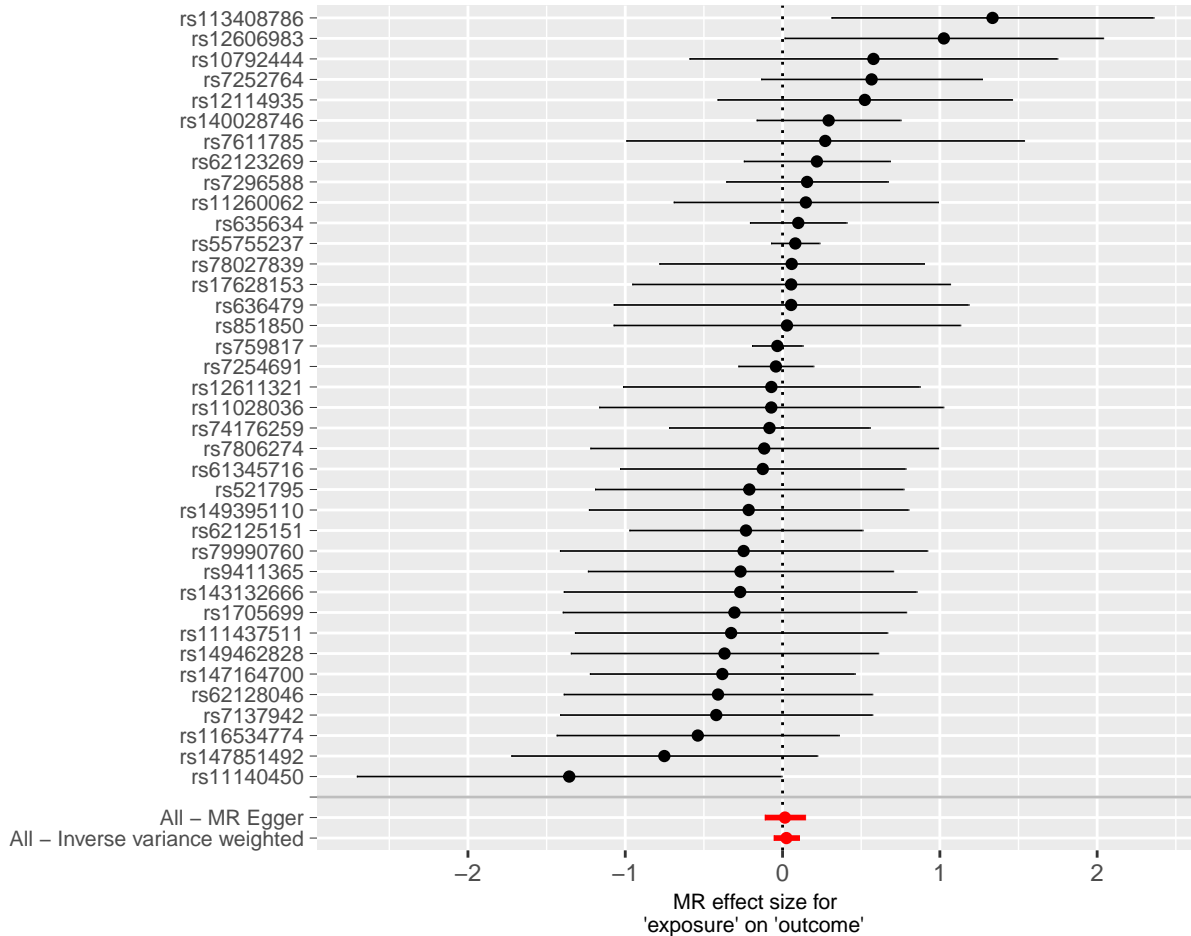

Supplement: Supplementary file 1 [file Data_Sheet_1.ZIP › data/GCST90274768_5e6/768.forest.pdf]

# MR Method

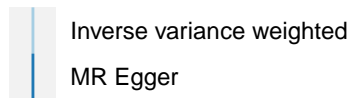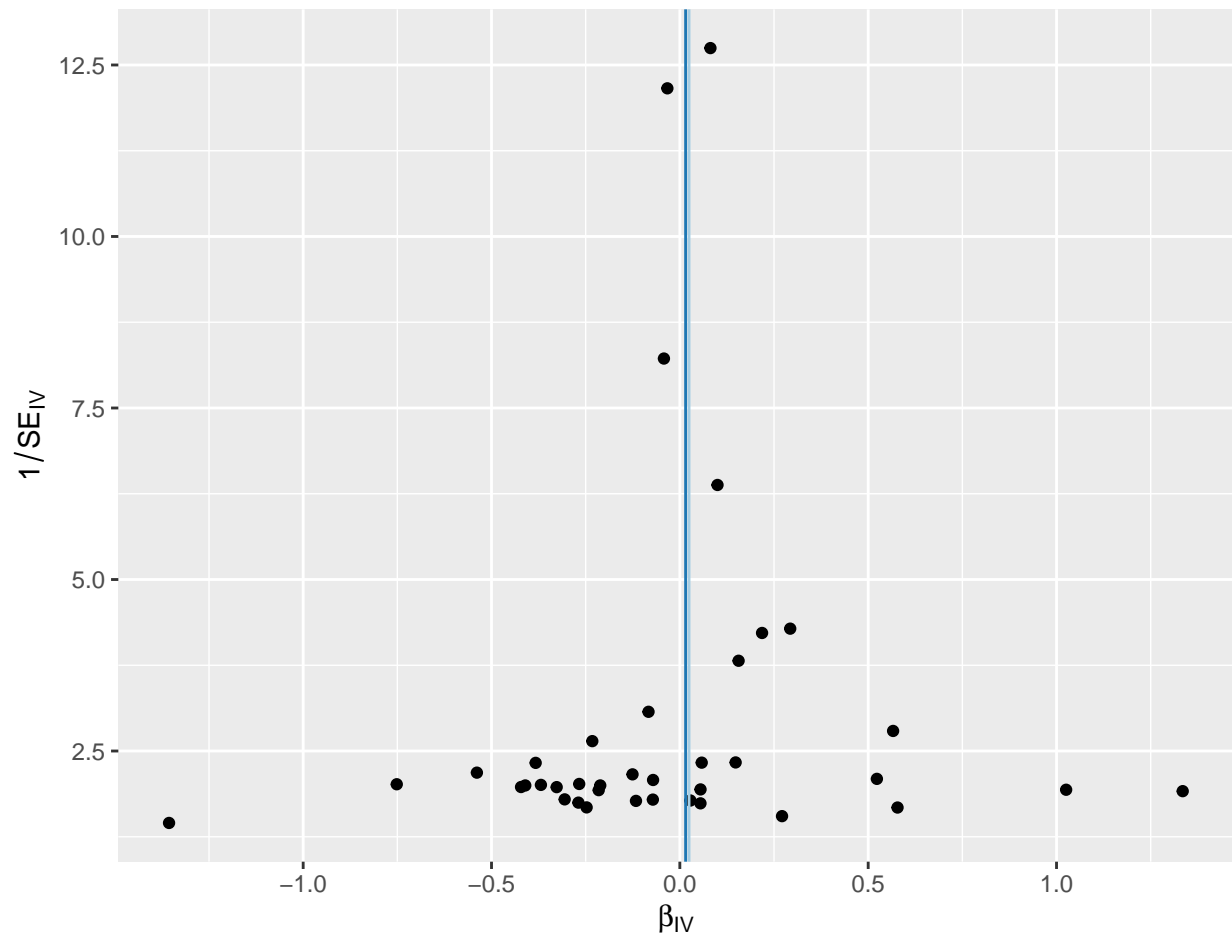

Supplement: Supplementary file 1 [file Data_Sheet_1.ZIP › data/GCST90274768_5e6/768.funnel_plot.pdf]

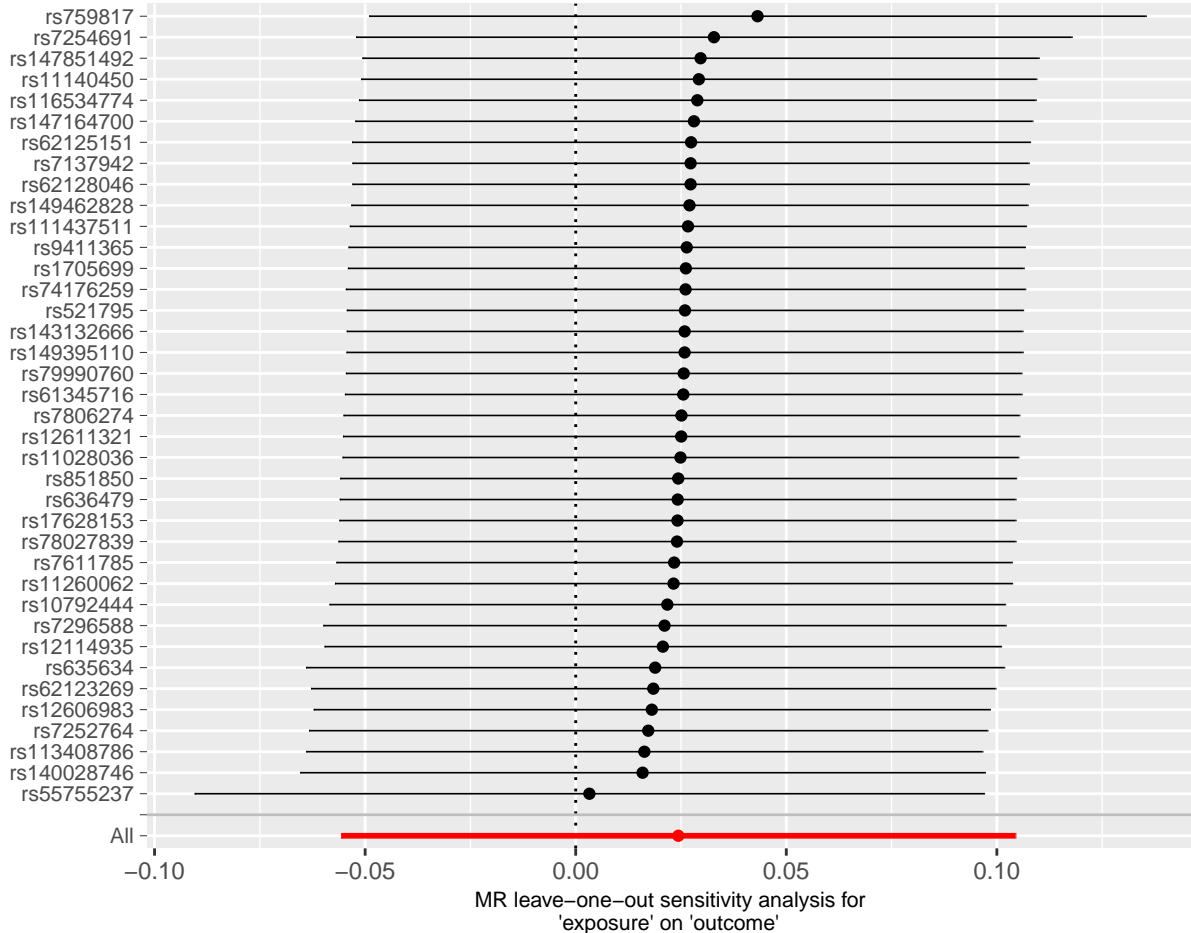

Supplement: Supplementary file 1 [file Data_Sheet_1.ZIP › data/GCST90274768_5e6/768.leaveoneout.pdf]

# MR Test

- Inverse variance weighted
- MR Egger
- Simple mode
- Weighted median
- Weighted mode

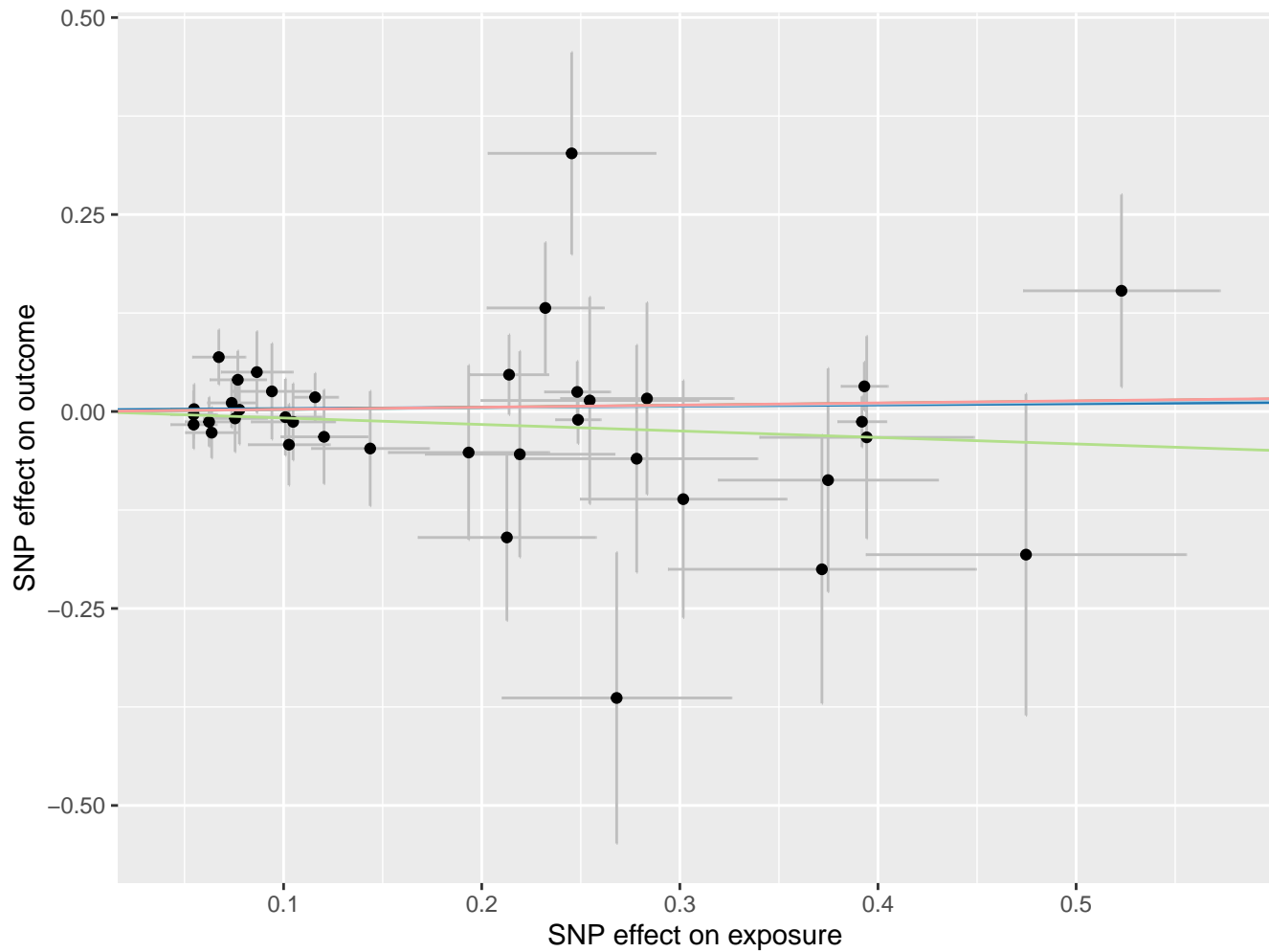

Supplement: Supplementary file 1 [file Data_Sheet_1.ZIP › data/GCST90274768_5e6/768.scatter_plot.pdf]

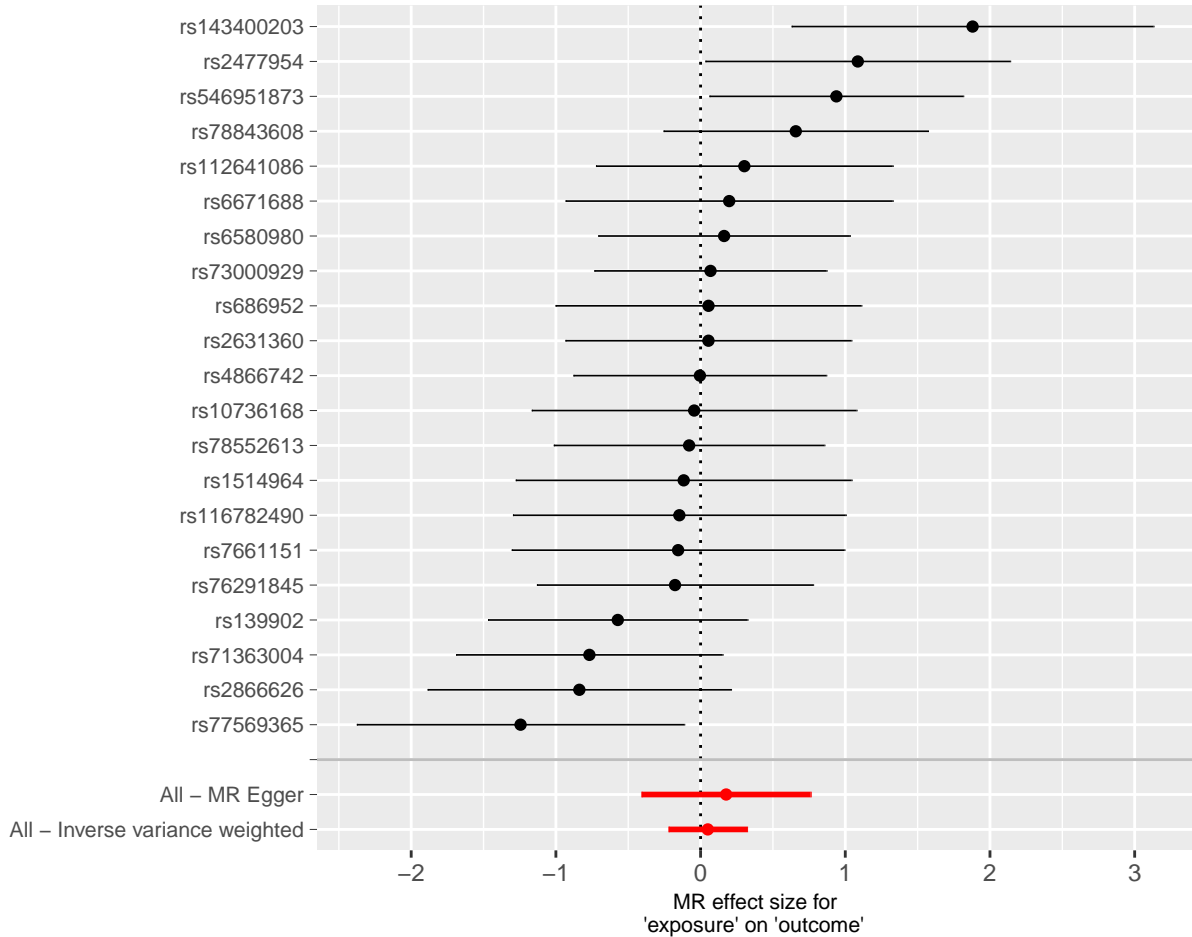

Supplement: Supplementary file 1 [file Data_Sheet_1.ZIP › data/GCST90274769_5e6/769.forest.pdf]

# MR Method

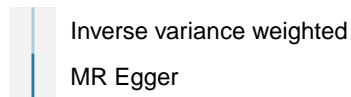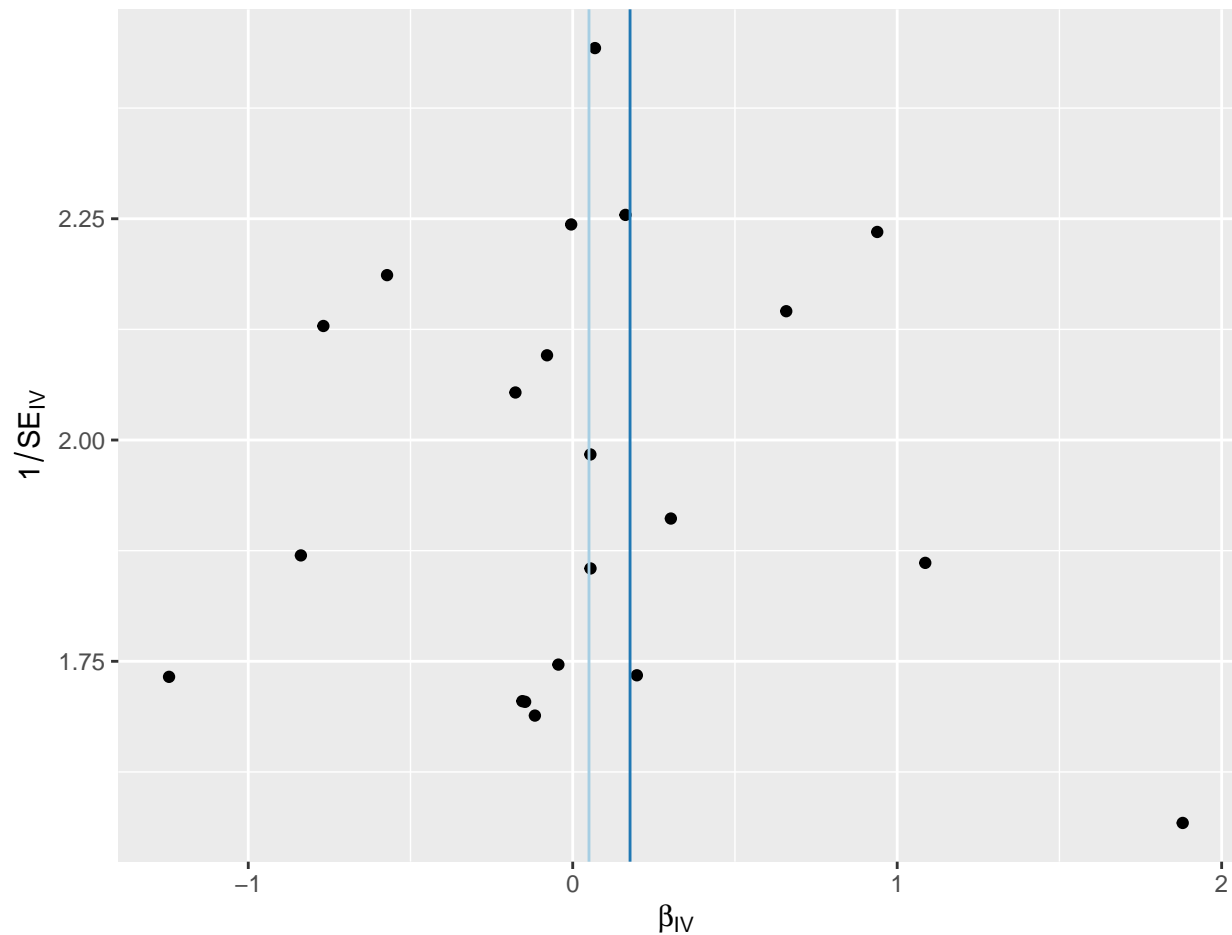

Supplement: Supplementary file 1 [file Data_Sheet_1.ZIP › data/GCST90274769_5e6/769.funnel_plot.pdf]

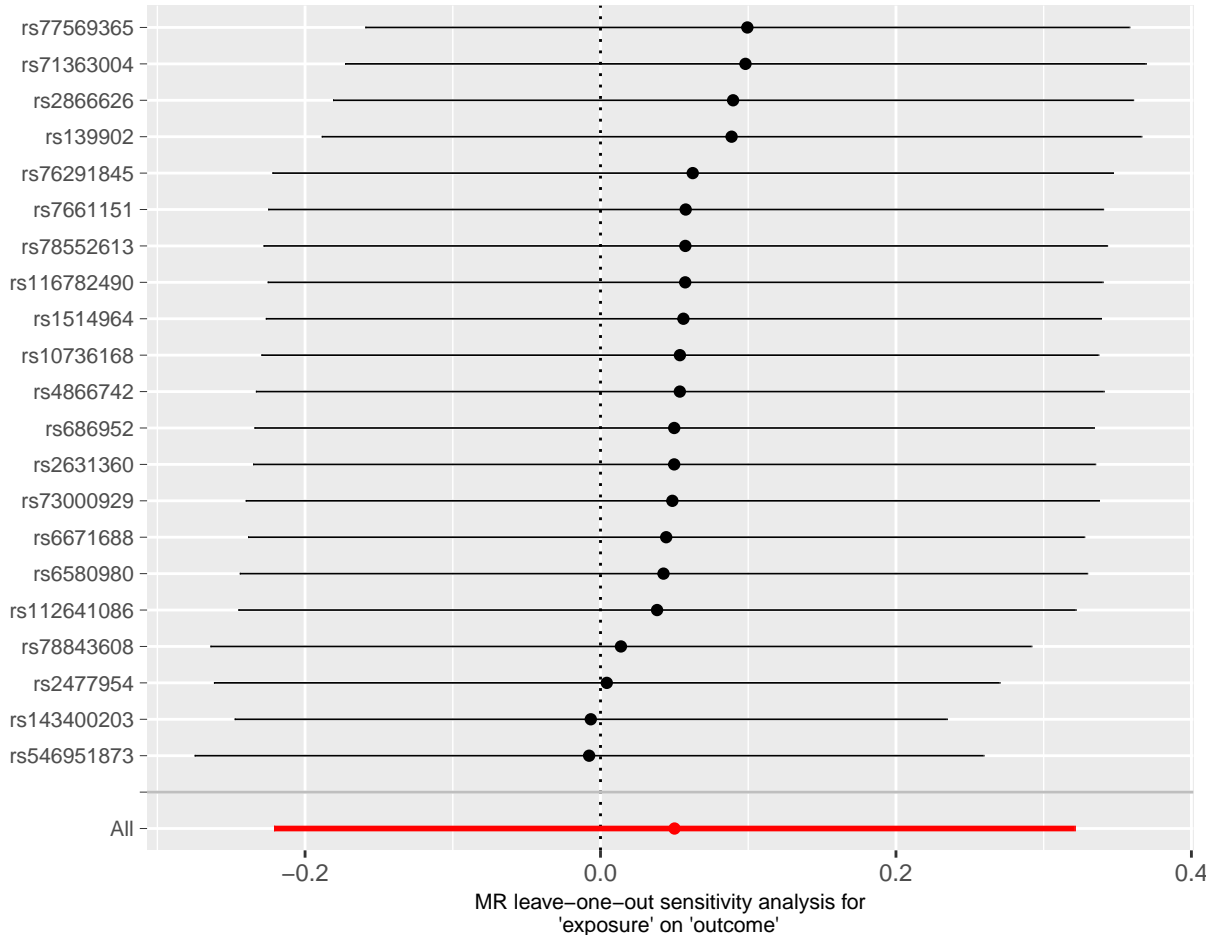

Supplement: Supplementary file 1 [file Data_Sheet_1.ZIP › data/GCST90274769_5e6/769.leaveoneout.pdf]

# MR Test

- Inverse variance weighted
- MR Egger
- Simple mode
- Weighted median
- Weighted mode

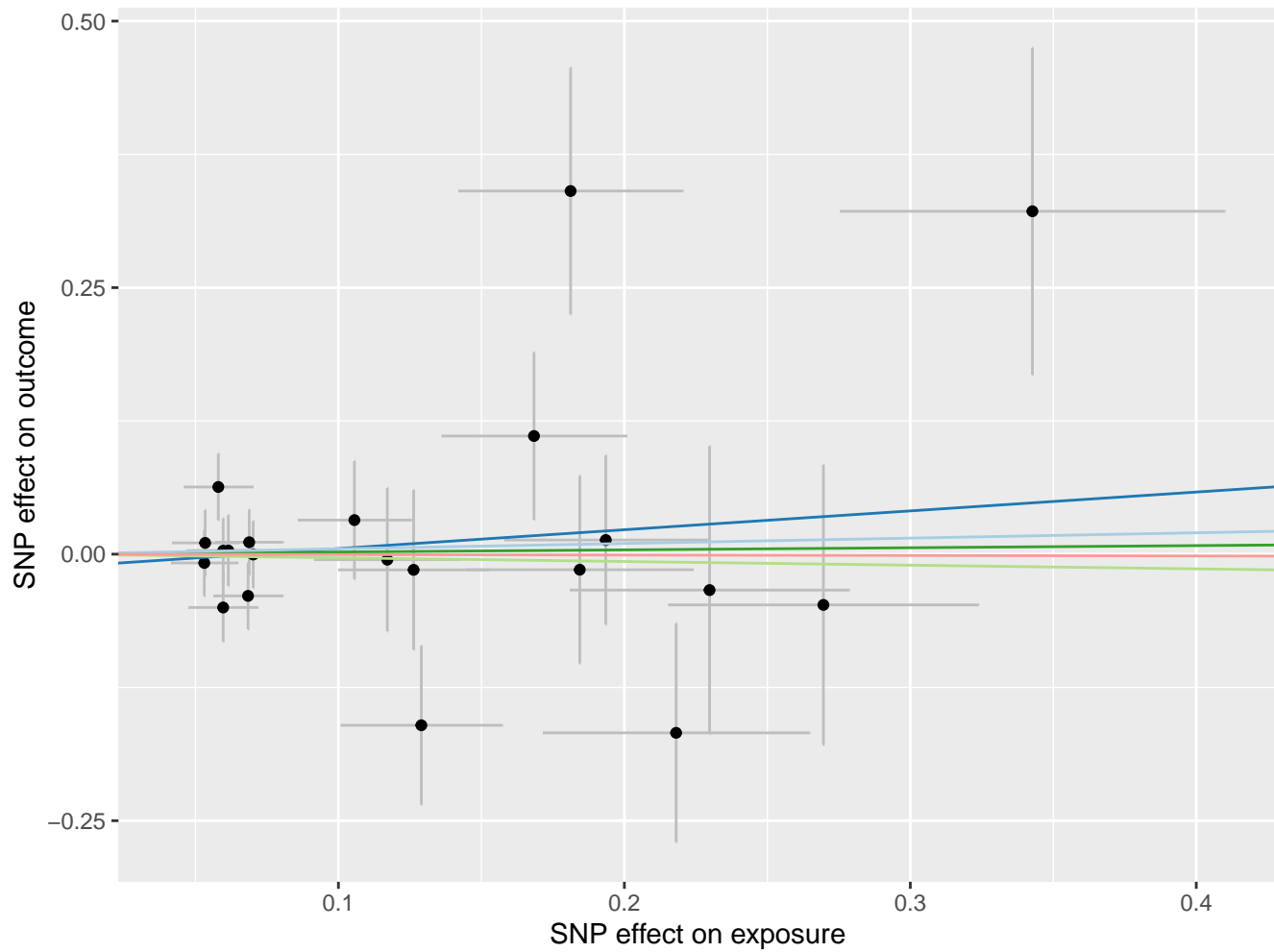

Supplement: Supplementary file 1 [file Data_Sheet_1.ZIP › data/GCST90274769_5e6/769.scatter_plot.pdf]

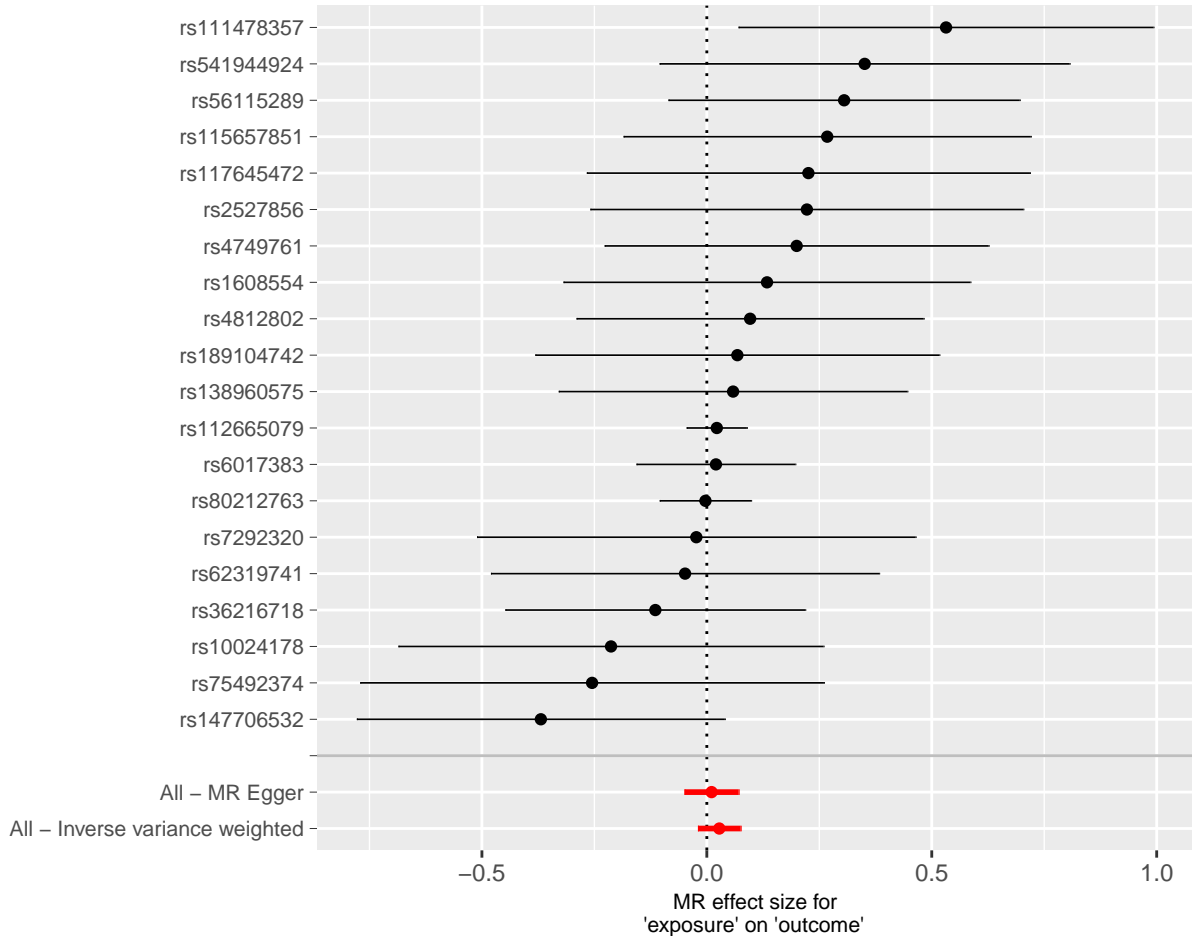

Supplement: Supplementary file 2 [file Data_Sheet_2.ZIP › ADA_TNF_TSMR/ada_tnf.forest.pdf]

# MR Method

- Inverse variance weighted
- MR Egger

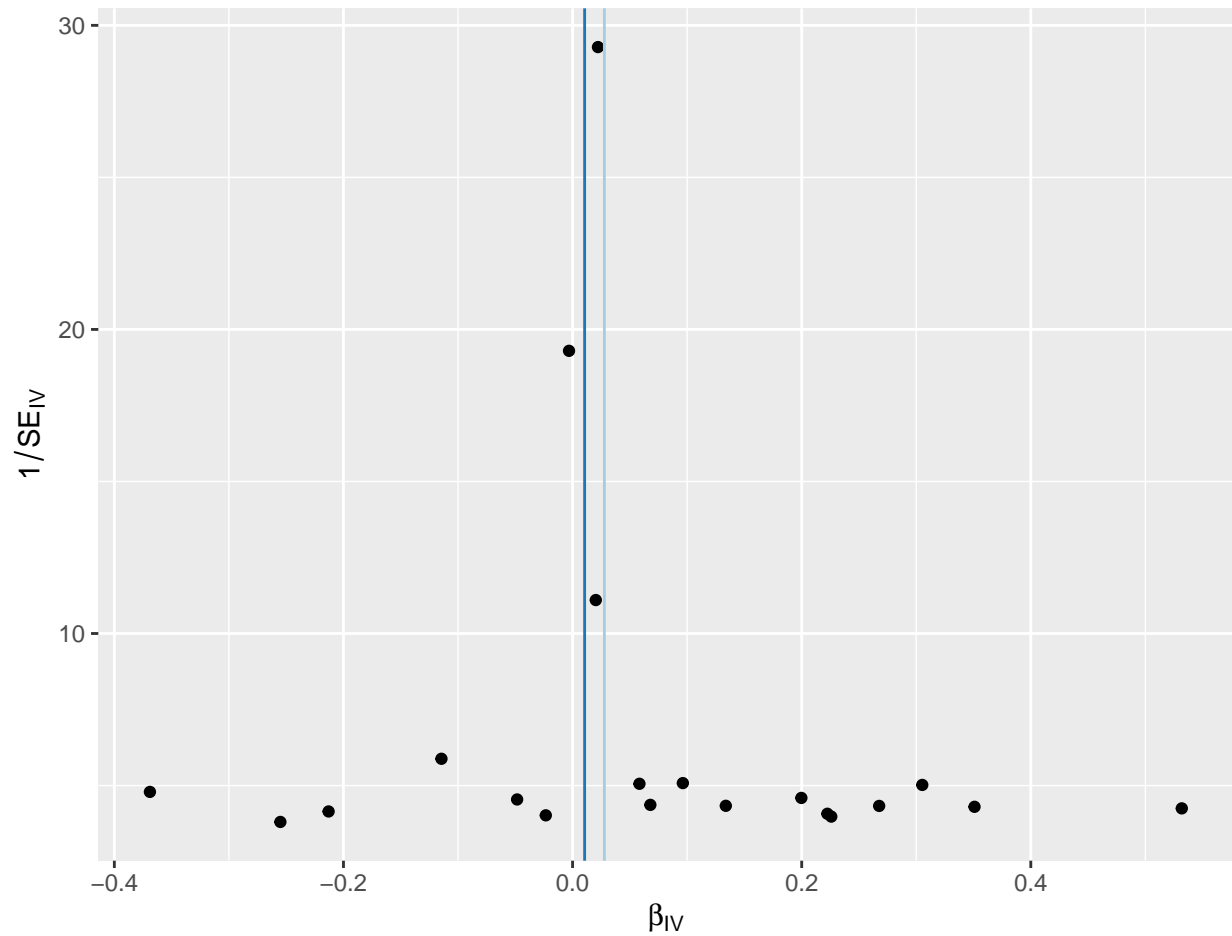

Supplement: Supplementary file 2 [file Data_Sheet_2.ZIP › ADA_TNF_TSMR/ada_tnf.funnel_plot.pdf]

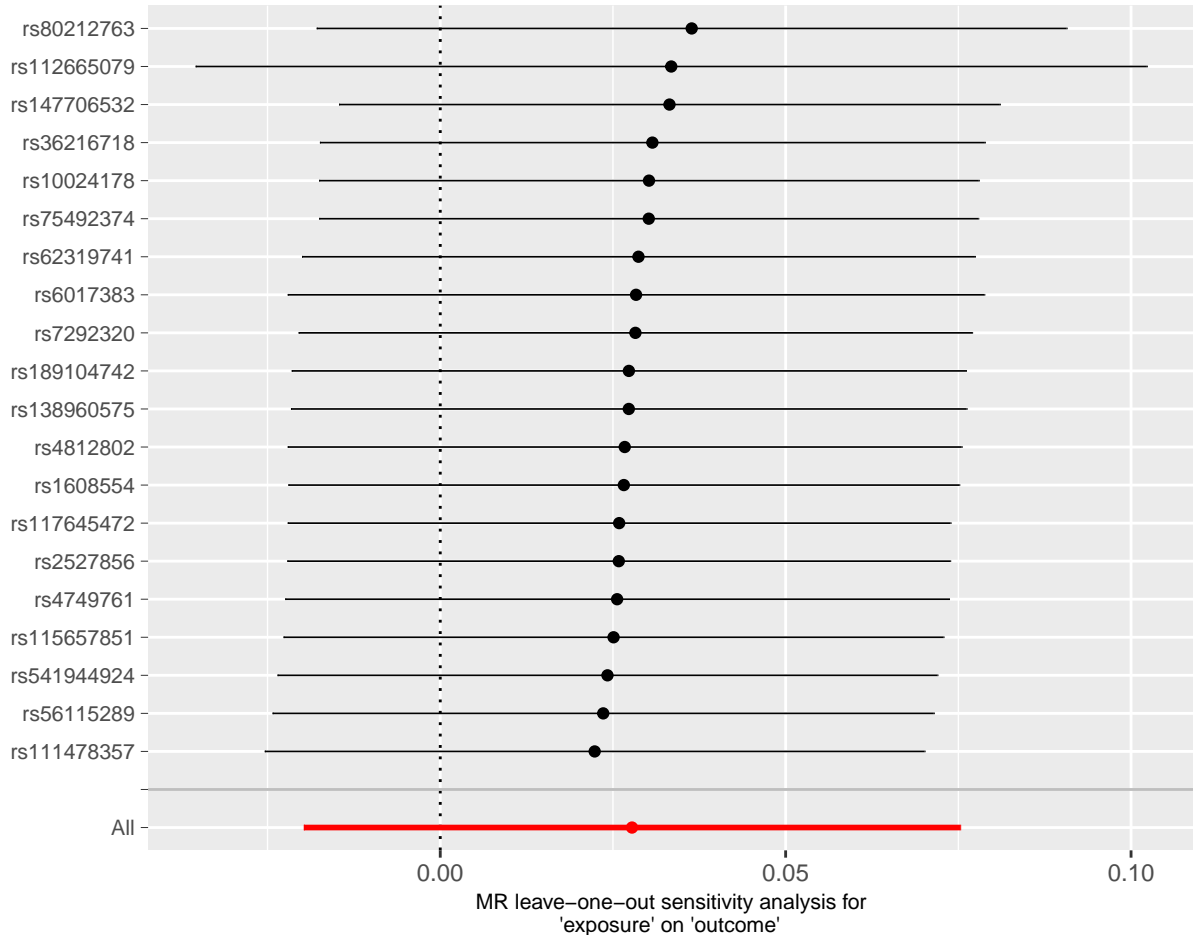

Supplement: Supplementary file 2 [file Data_Sheet_2.ZIP › ADA_TNF_TSMR/ada_tnf.leaveoneout.pdf]

# MR Test

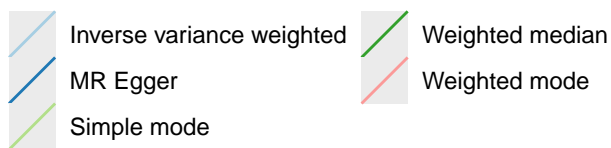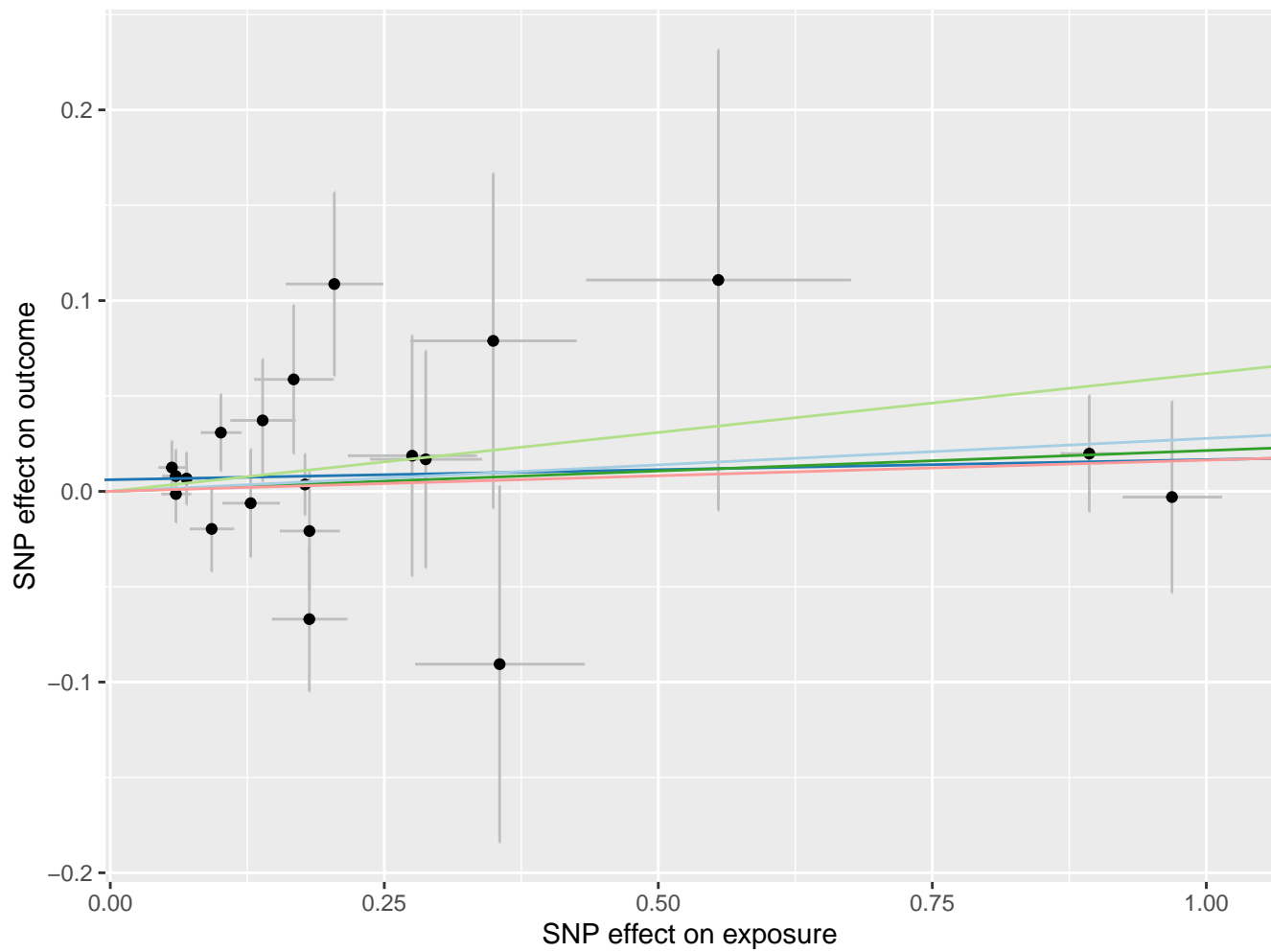

Supplement: Supplementary file 2 [file Data_Sheet_2.ZIP › ADA_TNF_TSMR/ada_tnf.scatter_plot.pdf]

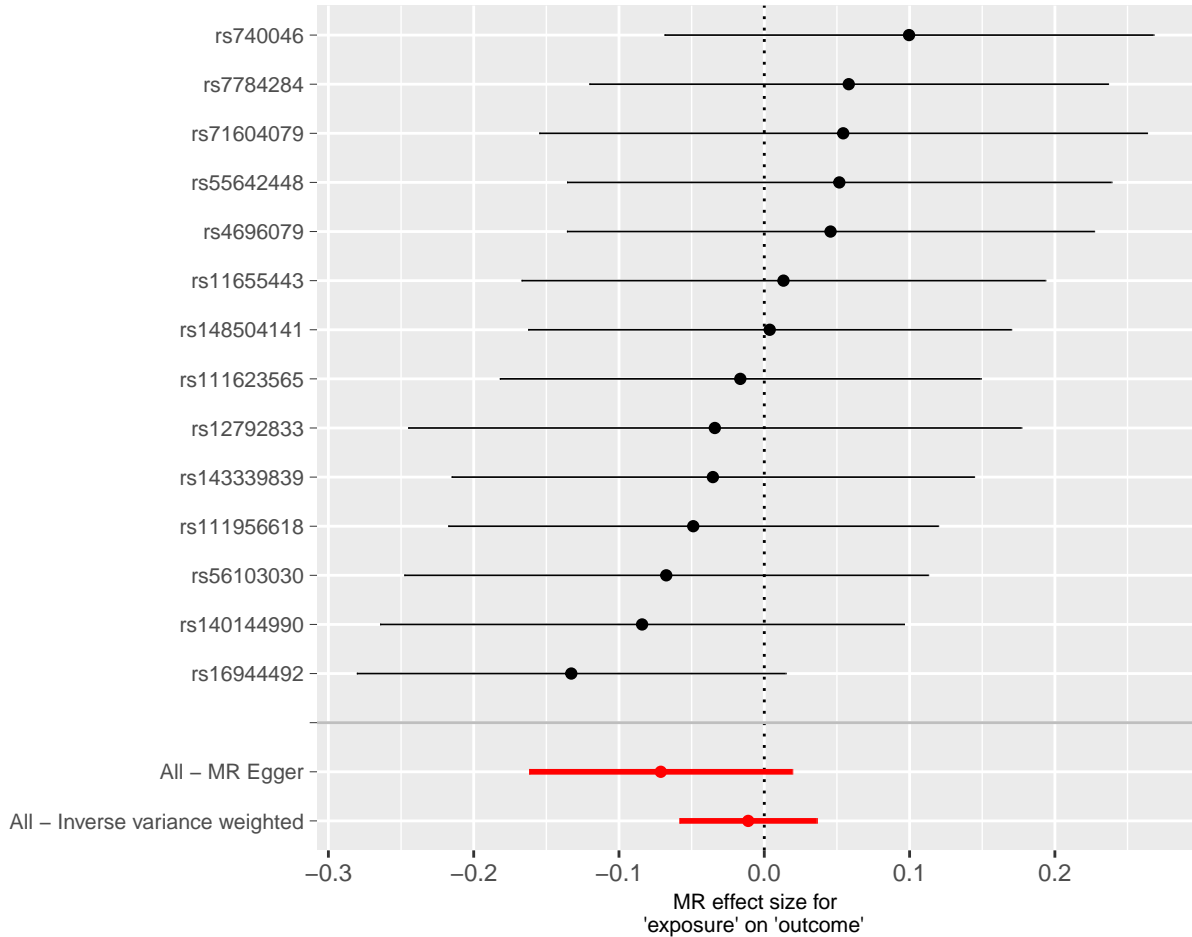

Supplement: Supplementary file 2 [file Data_Sheet_2.ZIP › KOA_TNF_TSMR/koa_tnf.forest.pdf]

# MR Method

- Inverse variance weighted
- MR Egger

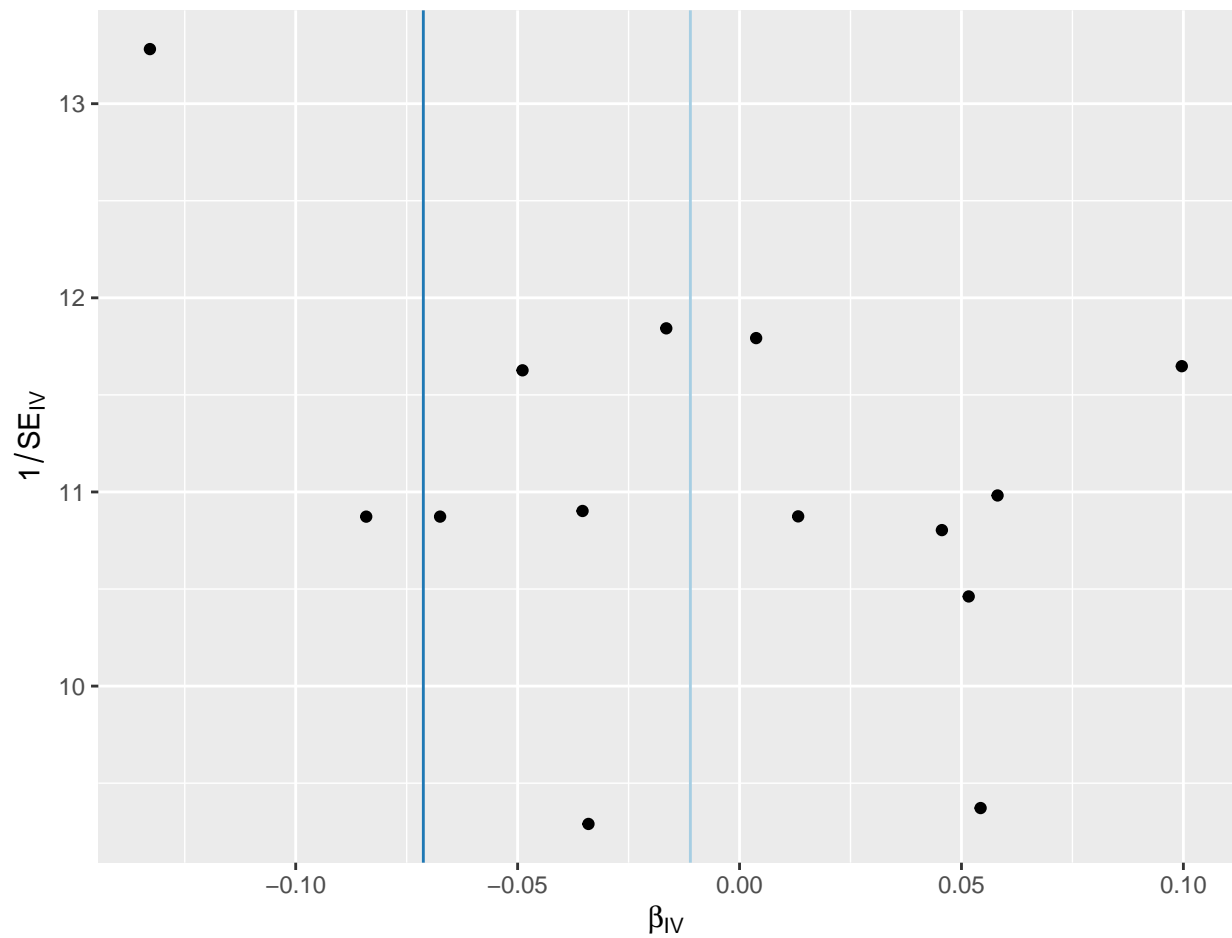

Supplement: Supplementary file 2 [file Data_Sheet_2.ZIP › KOA_TNF_TSMR/koa_tnf.funnel_plot.pdf]

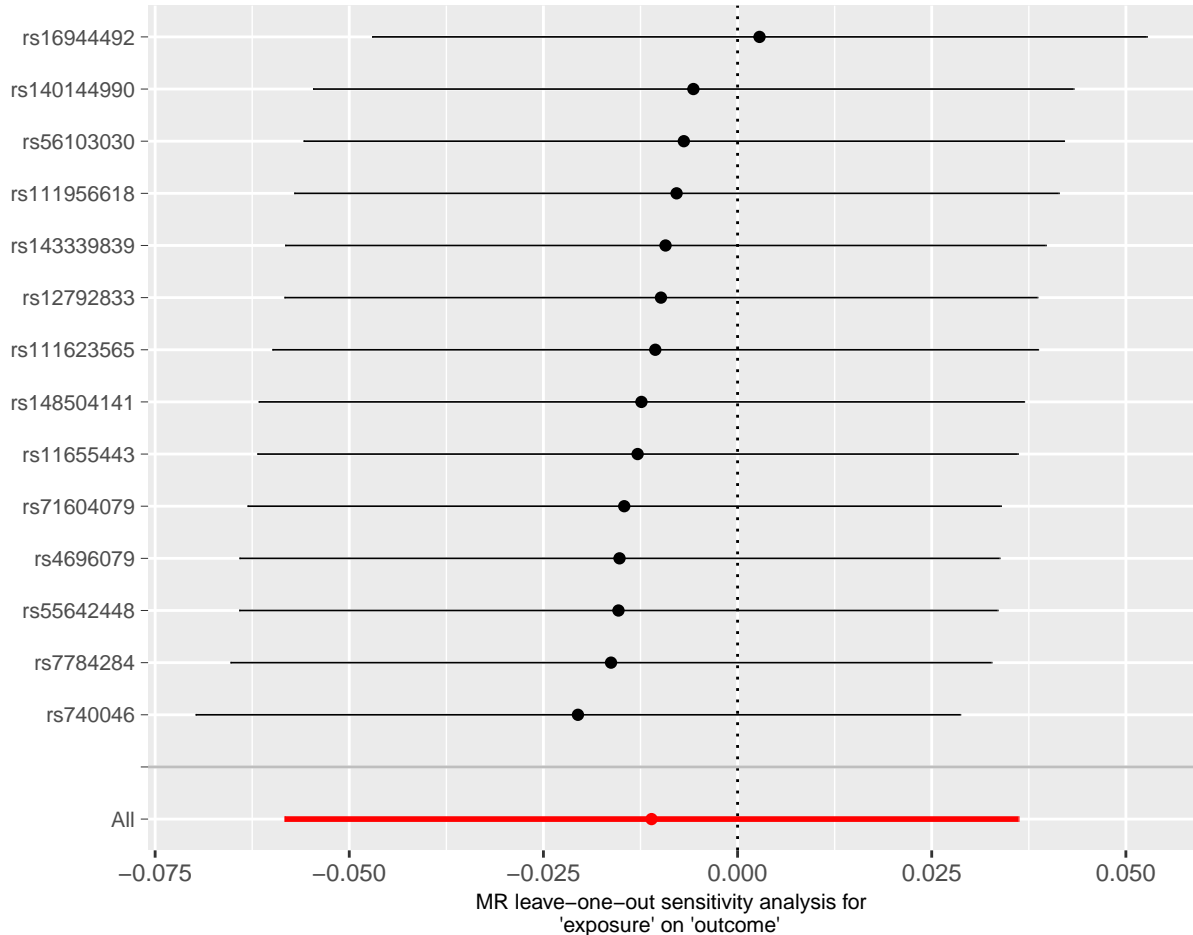

Supplement: Supplementary file 2 [file Data_Sheet_2.ZIP › KOA_TNF_TSMR/koa_tnf.leaveoneout.pdf]

# MR Test

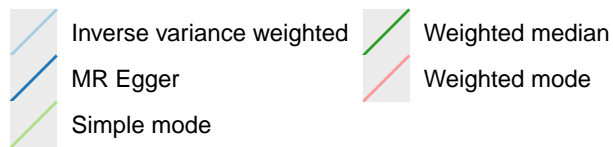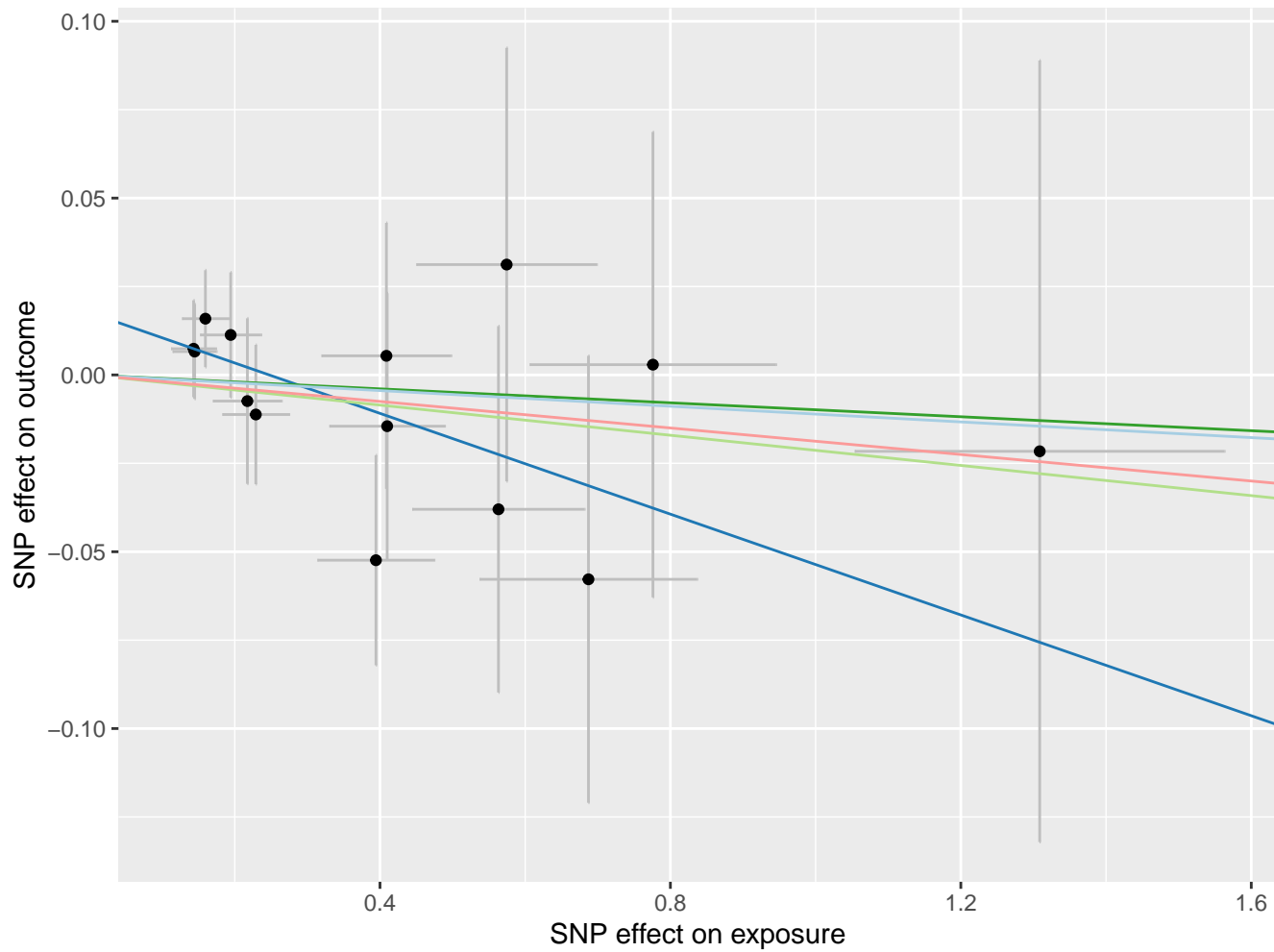

Supplement: Supplementary file 2 [file Data_Sheet_2.ZIP › KOA_TNF_TSMR/koa_tnf.scatter_plot.pdf]

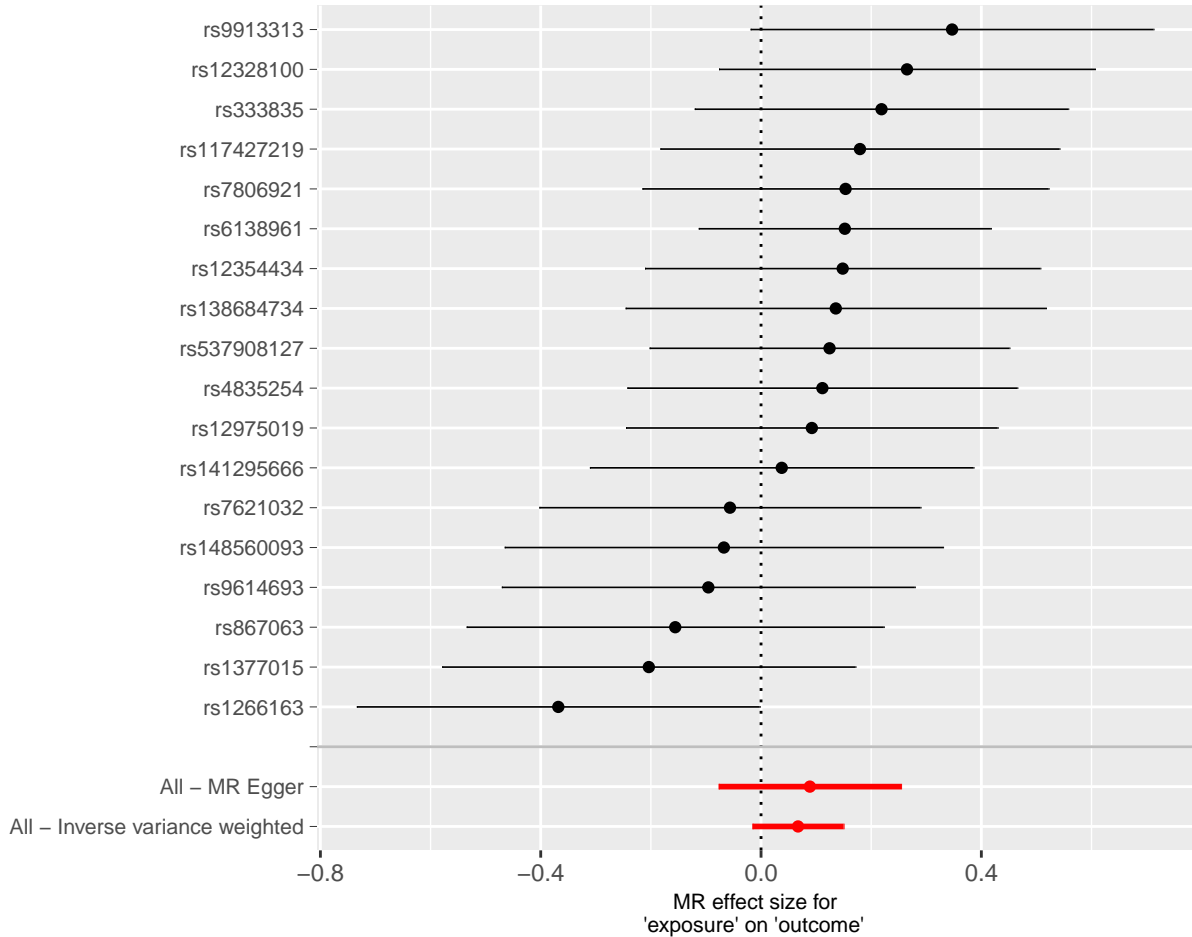

Supplement: Supplementary file 2 [file Data_Sheet_2.ZIP › TNF_CSF_TSMR/tnf_csf.forest.pdf]

# MR Method

- Inverse variance weighted
- MR Egger

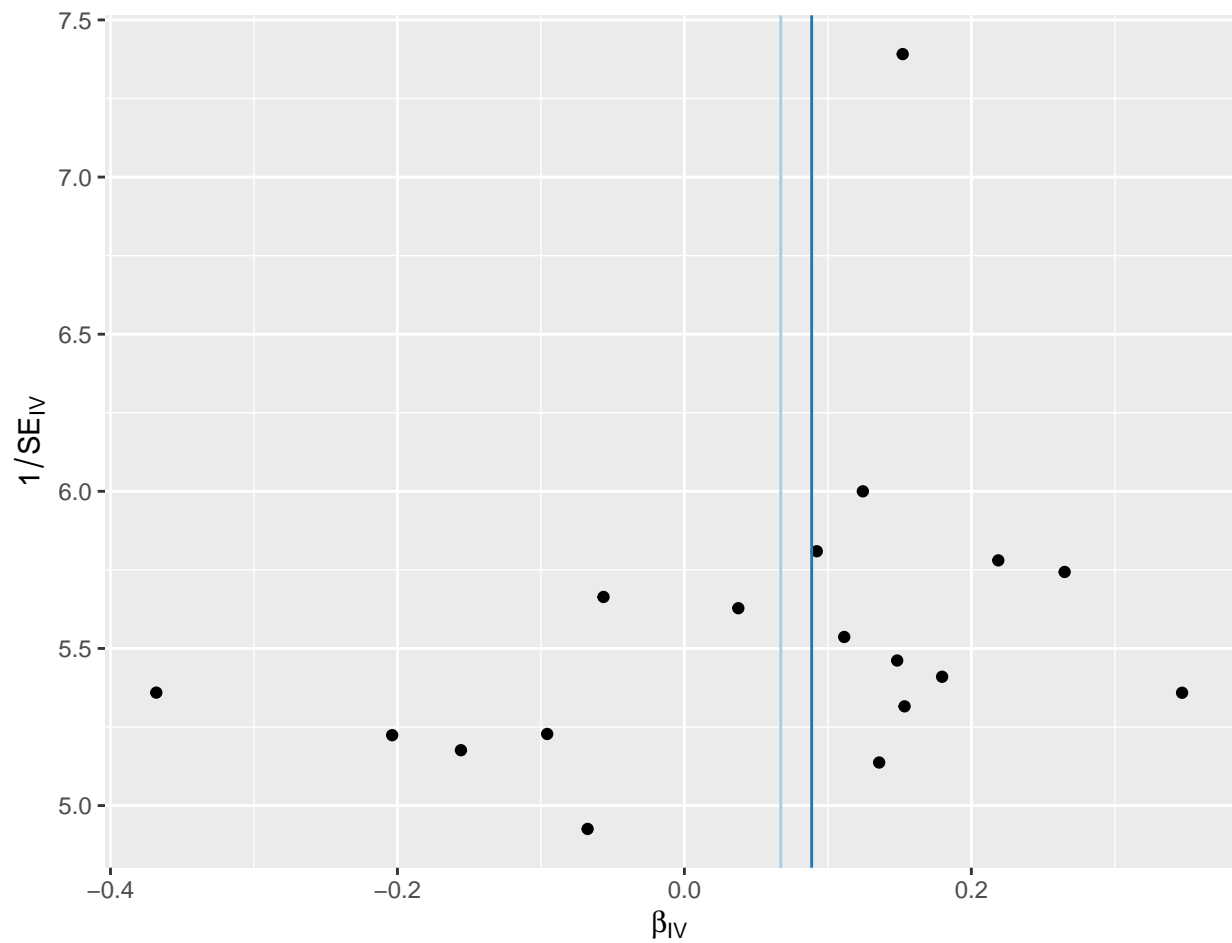

Supplement: Supplementary file 2 [file Data_Sheet_2.ZIP › TNF_CSF_TSMR/tnf_csf.funnel_plot.pdf]

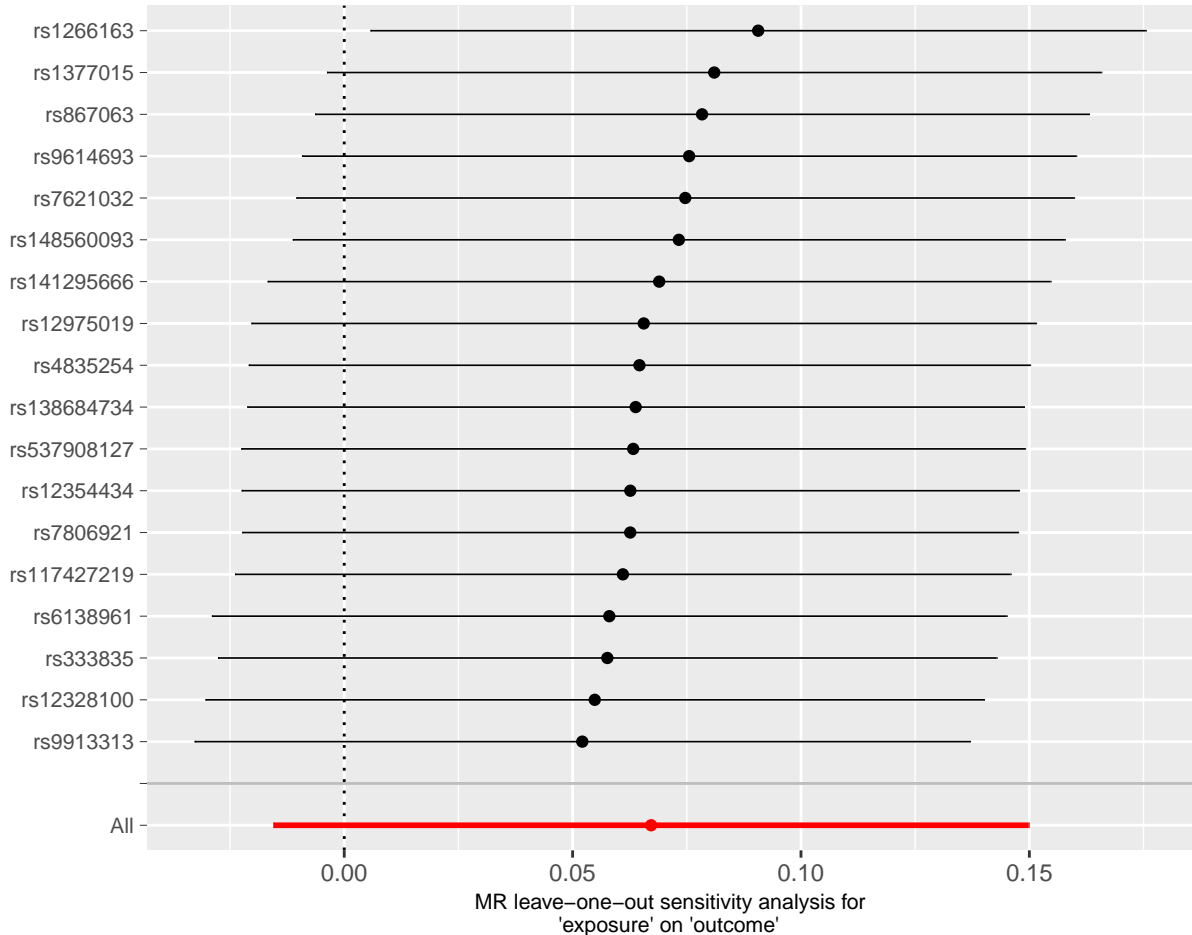

Supplement: Supplementary file 2 [file Data_Sheet_2.ZIP › TNF_CSF_TSMR/tnf_csf.leaveoneout.pdf]

# MR Test

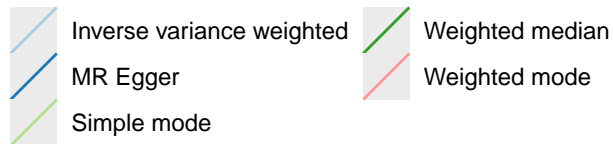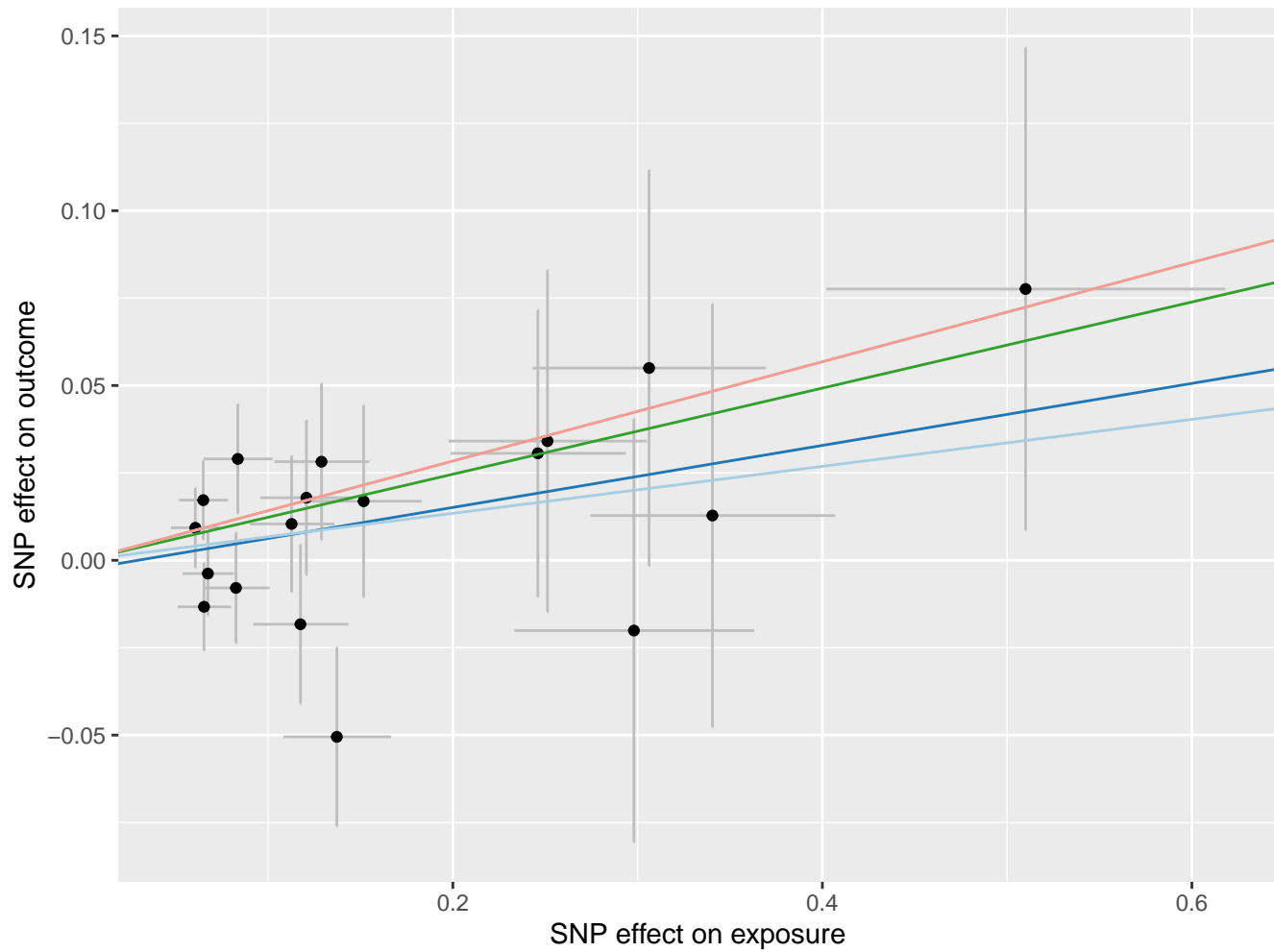

Supplement: Supplementary file 2 [file Data_Sheet_2.ZIP › TNF_CSF_TSMR/tnf_csf.scatter_plot.pdf]
