# Supplementary material for: Evidence supported by Mendelian randomization: impact on inflammatory factors in knee osteoarthritis
Source: Front Med (Lausanne). 2024 May 28;11:1382836. doi: 10.3389/fmed.2024.1382836 (PMC11165061; doi:10.3389/fmed.2024.1382836)
Supplement: Supplementary file 3 [file Data_Sheet_3.PDF]

Instrumental variable selection

	Significance Threshold	Clumping
ADA<br>TNFα<br>CSF1<br>KOA	5×10^{-6}	Kb = 5000;<br>R2 = 0.01

Reverse MR

Exposure	Outcome	SNPs	Beta_ivw	P_ivw
KOA	ADA	14	-0.020	0.456
KOA	TNFα	14	-0.011	0.646

After clarifying that there is no reverse causality interference between ADA, TNFα, and KOA, we supplemented our analysis with two-sample MR and MVMR.

Two-sample MR

Exposure	Outcome	SNPs	Beta_ivw	SE	P_ivw
**Primary analysis**					
ADA	KOA	19	-0.149	0.057	0.008
CSF1	KOA	17	0.329	0.107	0.002
TNFα	KOA	18	0.277	0.108	0.011
**Supplementary analysis**					
ADA	TNFα	20	0.028	0.024	0.252
TNFα	CSF1	18	0.067	0.042	0.111

Multivariate MR

Exposure	Outcome	SNPs	Beta_ivw	P_ivw	SE	Heter.P
ADA	KOA	37	-0.103	0.068	0.057	0.665
TNFα			0.379	< 0.001	0.106	
TNFα	KOA	35	0.312	0.005	0.109	0.405
CSF1			0.148	0.176	0.110	

Result and Conclusion:

Two-sample Mendelian randomization (MR) results indicate no causal relationship between ADA and TNFα (β_{IWV} = 0.028; p = 0.252). Similarly, there was no association found between TNFα and CSF1 (β = 0.067; p = 0.111). The multivariable Mendelian randomization (MVMR) analysis revealed a significant causal relationship between TNFα and KOA (β_{IWV} = 0.379; $p < 0.001$). After adjusting for the influence of TNFα, the analysis results between ADA and KOA did not support ADA as a risk factor (β_{IWV} = -0.103; p = 0.068). When CSF1 was considered as a potential confounder to adjust TNFα's effect, the MV-IVW results still showed TNFα's effect as significant (β = 0.312; p = 0.005), consistent with our initial two-sample MR analysis results. Conversely, correcting CSF1's effect for TNFα as a potential confounder contradicted the reported mechanism in the literature, which might explain the lack of association for CSF1 in the MV-IVW results.

Due to the lack of support for a correlation between ADA and TNFα in our results, we reasonably speculate that the impact of TNFα on ADA may not be significant. Similarly, the impact of CSF1 on TNFα may also not be significant.

Our research results show discrepancies with existing literature, indicating that further analysis with larger datasets may be necessary. In conclusion, we continue to support ADA as a potential protective factor and TNFα as a potential risk factor.
